# Prospects and challenges of implementing DNA metabarcoding for high-throughput insect surveillance

**DOI:** 10.1093/gigascience/giz092

**Published:** 2019-07-30

**Authors:** Alexander M Piper, Jana Batovska, Noel O I Cogan, John Weiss, John Paul Cunningham, Brendan C Rodoni, Mark J Blacket

**Affiliations:** 1 Agriculture Victoria Research, AgriBio Centre, 5 Ring Road, Bundoora 3083, VIC, Australia; 2 School of Applied Systems Biology, La Trobe University, Bundoora 3083, VIC, Australia

**Keywords:** biosecurity, alien species, biosurveillance, early detection, bioinformatics, reference database, quality assurance, controls, validation, non-destructive

## Abstract

Trap-based surveillance strategies are widely used for monitoring of invasive insect species, aiming to detect newly arrived exotic taxa as well as track the population levels of established or endemic pests. Where these surveillance traps have low specificity and capture non-target endemic species in excess of the target pests, the need for extensive specimen sorting and identification creates a major diagnostic bottleneck. While the recent development of standardized molecular diagnostics has partly alleviated this requirement, the single specimen per reaction nature of these methods does not readily scale to the sheer number of insects trapped in surveillance programmes. Consequently, target lists are often restricted to a few high-priority pests, allowing unanticipated species to avoid detection and potentially establish populations.

DNA metabarcoding has recently emerged as a method for conducting simultaneous, multi-species identification of complex mixed communities and may lend itself ideally to rapid diagnostics of bulk insect trap samples. Moreover, the high-throughput nature of recent sequencing platforms could enable the multiplexing of hundreds of diverse trap samples on a single flow cell, thereby providing the means to dramatically scale up insect surveillance in terms of both the quantity of traps that can be processed concurrently and number of pest species that can be targeted. In this review of the metabarcoding literature, we explore how DNA metabarcoding could be tailored to the detection of invasive insects in a surveillance context and highlight the unique technical and regulatory challenges that must be considered when implementing high-throughput sequencing technologies into sensitive diagnostic applications.

## Background

Increasing globalization of trade and tourism, along with changing climates, is expected to further increase the rate of biological invasions over coming decades [1–3]. Insects form a dominant component of this global spread of invasive species [4], posing a major threat to agroecosystems [5], the environment [6], and human health [7] through disruption of ecological networks, plant herbivory, and the transmission of pathogens and disease [8]. Once established in a new environment, ongoing containment and control of invasive insect pests imposes substantial costs to industry, government, and private landowners [8], and consequently major efforts are made to forecast incursion risk [9–11] and implement quarantine of entry pathways [12–14]. Despite these measures, the exponential increase in global movement of food, commerce, and humans complicates traceability and makes quarantine inspection of more than a fraction of arriving cargo an impossible task [15, 16]. Therefore, proactive post-border surveillance within agricultural and natural landscapes is becoming an increasingly important component of effective biosecurity programmes, aiming to detect invasive species early before populations escalate or spread and eradication becomes unfeasible [17–19].

Insect invasions can initiate and disperse across vast and highly heterogenous landscapes [20], and therefore surveillance programmes often involve extensive trapping conducted across a range of spatial scales, from large geographic areas to precise crop-monitoring activities within agricultural properties [21]. Because it is generally unclear whether a new introduction has occurred or what species it may be, surveillance programmes can extend over many years and target diverse taxonomic groups [22, 23]. In many cases surveillance traps will capture non-target endemic species in vast excess of the target pests and the sheer number of specimens that need to be sorted through and identified by highly trained entomologists forms a major diagnostic bottleneck. While insect diagnostics still largely relies on traditional morphological examination [24], in recent years this has been supplemented by a range of molecular techniques that allow standardized identification of a wide range of taxa without specialist taxonomic expertise (Table 1). DNA barcoding in particular has become a central component of the modern diagnostic toolbox, owing to the ability to compare a single unknown specimen against many potential species in a single assay, and standardized protocols that allow transparent and objective comparison of specimen identifications between laboratories, regulatory agencies, and trading partners [24–26]. Despite these advantages, the time-consuming process of conducting single PCR and sequencing reactions on individual specimens has restricted the use of DNA barcoding to confirming the identity of specimens already deemed suspect by prior morphological sorting, or for identification of taxa or life stages where a taxonomic key may not be available or key diagnostic structures are degraded or missing [24, 27]. Without access to a scalable and cost-effective diagnostic method for large trap catches, current surveillance programmes generally do not identify all specimens to species level [23, 28]. Instead, target lists are confined to relatively few priority pest species identified by previous risk assessment [9] or statistical methods are used to select only a subset of specimens for species-level identification [29]. These restrictions can result in the non-detection of unanticipated or cryptic invasive species that are not being actively monitored for [30].

**Table 1:** Methods used for insect identification, with suitability assessed according to accuracy, expertise, general applicability, time, and throughput criteria

Identification method	Taxonomic expertise	Identify specific taxa	Identify broad range of taxa	Throughput level	Time per identification
Morphological					
Microscopic examination	High	High*	High*	Low	Moderate
Molecular					
PCR–restriction fragment length polymorphism	Low	Moderate	Low	Moderate	Moderate
DNA barcoding	Low	High	High	Low	Moderate
Quantitative PCR/droplet digital PCR	Low	High	Low	High	Low
Loop-mediated isothermal amplification	Low	High	Low	Low	Low
Metabarcoding	Low	High	High	Very high	Low

^*^This morphological identification score assumes a high level of taxonomic knowledge and a low human error rate.

**Figure 1:**
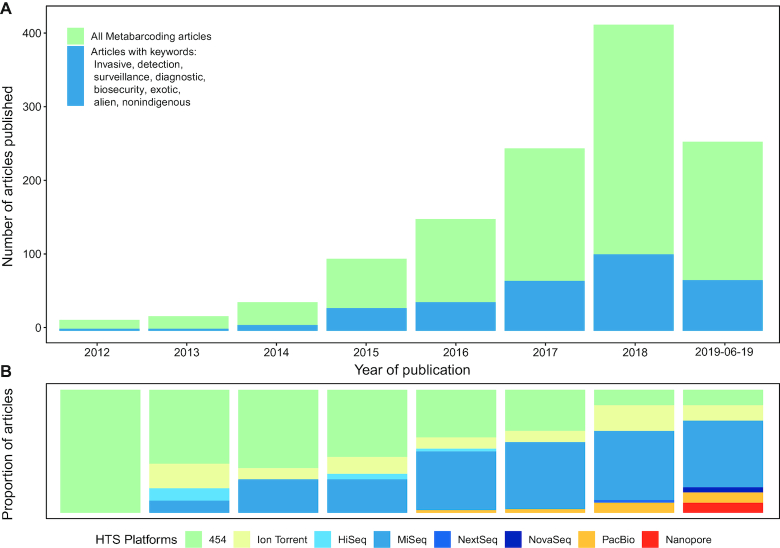
Metabarcoding in the literature. (A) Published articles obtained from Scopus, Crossref, and PubMed searches on 6 June 2019 for all metabarcoding studies, and those containing keywords in title or abstract relevant to invasive insect surveillance. (B) Sequencing platforms used in the above metabarcoding studies displayed as a proportion for each year.

In order to overcome the limitations of current identification methods for processing large numbers of specimens, recent studies have looked to high-throughput sequencing (HTS) technologies to allow DNA barcode-based identification to be conducted in a massively parallel manner. This process, termed “metabarcoding” [31] or “marker gene sequencing” [32], generates a large number of individual barcode sequences in a single reaction, enabling the simultaneous identification of individuals in large mixed communities [33, 34], such as a trap sample containing many different insect species. The ability to rapidly and cost-effectively survey biodiversity has led to metabarcoding being taken up across numerous fields of applied ecology [34–37], including the identification of invasive species (Fig. 1A) [33, 38–40]. By identifying both endemic and potential exotic species in a bulk DNA analysis approach, metabarcoding can obviate the time-consuming specimen sorting required by previous molecular and morphological diagnostic methods, and allow detection of not just key pests but also other unanticipated species that are not being actively searched for [38, 41, 42]. This aspect is particularly advantageous for the detection of environmental threats because when one considers impacts beyond just agriculture and the time lag that can occur between introduction of a new species and perceptible damage to the environment [43], it becomes clear that there are far more invasive species of threat than can be identified by risk assessment and incorporated into target lists [23, 44]. A further advantage arises from the ability of HTS to count occurrences of specific sequences in a mixed sample [45], potentially allowing simultaneous pest identification and population size estimation. Finally, the rapidly increasing output of HTS technologies enables multiplexing of hundreds of trap samples in a single sequencing run, providing an avenue to dramatically scale up insect surveillance to the level required for effective, affordable, and proactive management response.

**Figure 2:**
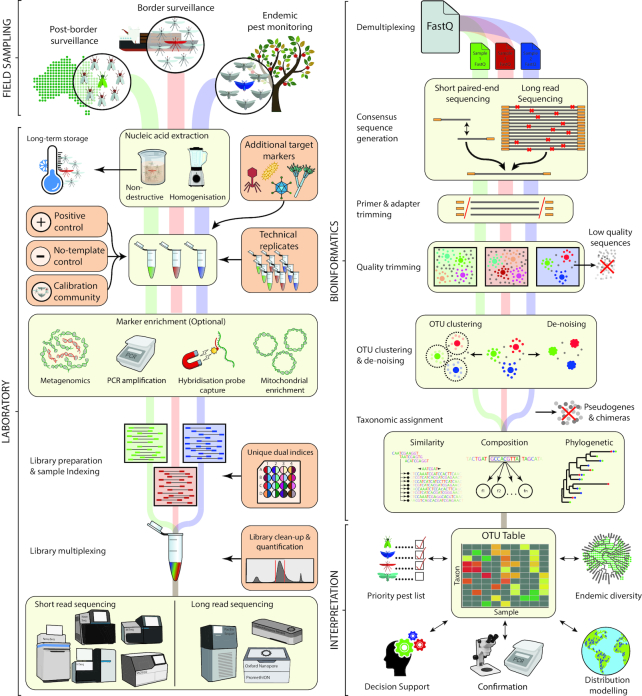
Overview of common metabarcoding workflows for identification of trapped insect species

Despite the advantages that metabarcoding may offer to insect surveillance programs, uptake of new diagnostic tools into operational use depends on more than just the cost-effectiveness of the tool, but also on factors such as ease of use, accuracy, reproducibility, and perceived usefulness to the end users, as well as compatibility with existing policy frameworks [46, 47]. With the introduction of the World Trade Organisation Agreement on the Application of Sanitary and Phytosanitary measures (SPS) came new obligations for exporting nations to demonstrate freedom of a geographic area from particular pests using scientifically rigorous surveillance practices [48]. This agreement has in turn led to harmonization of routine diagnostic procedures into internationally standardized protocols to ensure that all end users are aware of the particulars involved and therefore committed to accepting any risk management actions that arise through its use [46, 49]. The SPS agreement recognizes the International Plant Protection Convention (IPPC) and the World Organisation of Animal Health (OIE) as the international standard-setting bodies for plant and animal health, respectively [48], and adoption of new standards stems from exhaustive workgroup efforts by these agencies [13, 50]. While the opportunities that HTS approaches could offer have been widely recognized by the diagnostics community [51, 52], because of the relative infancy of the technology, standards and guidelines around their use is a rapidly evolving space and validated protocols do not yet exist. Despite this, there is flexibility within the SPS framework for trading partners to introduce novel sanitary or surveillance procedures if it can be demonstrated that they are equivalent to or better than previous methods [49] and both the IPPC and OIE have now released guidelines for those laboratories preparing to implement HTS approaches in routine diagnostics applications. These guidelines highlight the need for robust experimental designs, assay validation, and quality assurance [51, 53, 54], reflecting recent discussions in the wider metabarcoding community [55]. In this review we explore the application of metabarcoding for high-throughput species-level identification of insects, providing an overview of common metabarcoding workflows (Fig. 2) and considerations required at each step to ensure reliable detection and quantification of taxa within complex mixed communities. We further discuss the unique technical and regulatory challenges of integrating broad-spectrum HTS assays into diagnostic laboratories and offer a perspective on the future adoption of high-throughput insect surveillance within international biosecurity frameworks.

## Review

### Selecting a taxonomic marker

Appropriate selection of a taxonomic marker or barcode locus is a critical first step in design of a metabarcoding assay because all downstream species detection and identification will rely on how conserved this marker is across taxa, and the discriminatory power of the nucleotide variation contained within it [56]. The markers most commonly used in metabarcoding studies are those already widely adopted for conventional DNA barcoding, and therefore the mitochondrial cytochrome oxidase I (COI) locus has been the most widely used marker for metabarcoding of insects to date. The 658-bp region of COI [57] used for conventional DNA barcoding has a strong track record of delivering species-level identification of insect pests [58]; however, many HTS platforms impose strict limitations in molecule length that can be sequenced (Table 2) and therefore smaller stretches of the conventional barcode loci or “mini-barcodes” must be used [59]. Nevertheless, research into degraded DNA samples has shown that singular COI barcode of sizes between 135 [60] and 250 bp [61] can reliably distinguish most animal species; however, appropriate placement within the larger barcode region is essential [62]. Despite the excellent taxonomic resolution provided by COI, since its application to metabarcoding a number of further limitations have become particularly apparent. Because COI is a protein-coding gene, the third position of codons can be variable, leaving no strictly conserved nucleotide sites for design of universal PCR primers [63]. This mismatch inevitably leads to primers having variable affinity for different template molecules, biasing the amplification towards well-matched taxa and failing to amplify others [64]. Unlike conventional DNA barcoding where a failed amplification will result in a noticeably absent PCR product, in a bulk sample failed amplification of a particular taxon will be masked by the recovery of sequences from other taxa and therefore will go unnoticed [63]. A further issue inherent to mitochondrial loci such as COI is the proliferation of nuclear mitochondrial pseudogenes (numts) in many insect orders [65–67], the result of historical recombination between the mitochondrial and nuclear genomes [68]. Co-amplification or preferential amplification of these pseudogenes instead of the true mitochondrial locus can complicate species identification [67] and result in overestimation of taxonomic diversity in the sample [69].

**Table 2:** Comparison of sequence throughputs, error rate, and associated costs among high-throughput sequencing platforms

Short-read platforms								Long-read platforms			
	Illumina MiSeq	Illumina NextSeq	Illumina HiSeq 3000/4000	Illumina NovaSeq	MGISeq-200	MGISeq-2000	MGISeq-T7	PacBio Sequel	PacBio Sequel II	ONT MinION	ONT PromethION
Maximum throughput (Gb)	15	120	750/1,500 (8/16 lanes)	6,000 (8 lanes)	60	1,080	6,000	20	160	20	150 per flow cell (up to 48)
Maximum read length	2 × 300 bp	2 × 150 bp	2 × 150 bp	2 × 150 bp	2 × 100 bp	2 × 150 bp	2 × 150 bp	∼100 kb	∼100 kb	∼2 Mb	∼2 Mb
Error rate	Low	Low	Low	Low	Low	Low	Low	Low (consensus error)	Low (consensus error)	High	High
Instrument cost	Low	Medium	High	High	Low	Medium	High	High	High	Extremely low	Low
Set-up time (labour)	Medium	Medium	Medium	Medium	Medium	Medium	Medium	High	High	Low	Low
Run time (hours)	56	30	84	40	<48	<48	24	15	15	1–72	1–72
Sequencing cost per sample*,^†^	<$50	<$15	<$10	<$5	<$50	<$10	<$5	<$25	<$15	<$25	<$5

^*^Costs are presented in Australian Dollars (AUD) and consider chemistry cost, depreciation, servicing, and computational cost over the lifespan of the instrument; however, total costs and read lengths will further depend on target enrichment and library preparation methods used.

^†^Assuming pooled sequencing of many traps with 250-Mb sequencing effort per sample.

**Figure 3:**
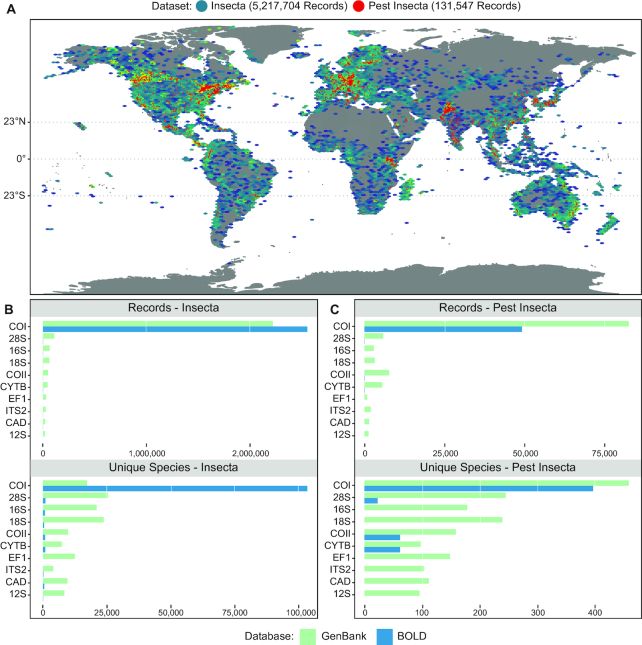
DNA barcodes in public reference databases. (A) Global distribution of all sufficiently annotated DNA barcode records from BOLD and GenBank for all barcode loci; records for all Insecta are displayed as a density map, while those species present on international pest lists are overlaid in red. (B) Distribution of records and unique species within major public databases for the 10 barcode markers with the most reference information for entire Insecta and for (C) Insecta species present on international pest lists.

As a result of the aforementioned issues, as well as the inability for COI to differentiate certain pest groups [70], a range of alternative universal barcode markers have been proposed (reviewed by Freeland [56]). Ribosomal RNA (rRNA) genes are particularly appealing owing to their high copy number and stem-loop structure that consists of highly conserved core sequences for primer binding, interspaced with variable regions providing taxonomic resolution [71, 72]. Despite this, rRNA regions are on average more conserved than COI and therefore while appropriate for reconstructing higher level relationships they require longer spans of nucleotides to be informative at the species level. For single-specimen barcoding this can be overcome by concatenating several markers to increase phylogenetic resolution [73]; however, this presents a challenge for metabarcoding of mixed communities because there is no way of knowing whether 2 non-overlapping markers are from the same individual [74]. Therefore, while multi-locus approaches can be useful for expanding the taxonomic diversity an assay can recover [75–77], in particular cross-kingdom diversity (Box 2), they do not necessarily provide greater resolution [45]. Consequently, closely related and difficult-to-diagnose pest taxa may require further studies to identify appropriate diagnostic loci [78], or the development of novel analytical methods to integrate taxonomic assignments from multiple independent barcode loci. Finally, the application of alternative markers to insect diagnostics will suffer from a lack of reference sequence data because many taxa, including those of economic importance, currently only have COI sequence data publicly available (Fig. 3B, 3C). Therefore, because species-level resolution is a requirement of many diagnostic standards [24, 49, 79], for the taxa in which it has sufficient resolution, the high mutation rate and extensive reference information obtainable for COI will maximize the utility of metabarcoding within a broad-spectrum surveillance programme [80].

Box 1: **Reference sequence databases**

As with conventional DNA barcoding, accurate taxonomic assignment in metabarcoding studies relies on a well-curated reference database of DNA marker sequences tied to vouchered morphological specimens to compare query sequences against [81]. The primary public nucleotide databases of relevance to insect metabarcoding are the Barcode of Life Data System (BOLD) [82] and the NCBI GenBank database [83]. While GenBank hosts greater overall sequence data, BOLD represents a curated DNA barcoding database that aims to maintain consistent links between sequences, validated morphological specimens, and associated specimen collection metadata [84]. Concerted efforts to generate mitochondrial COI barcodes for major insect orders have led to broad coverage of insects of biosecurity concern in both major public databases [58]; however, many geographic regions are still under-sampled (Fig. 3A) and reference sequences for alternative loci are mostly unavailable (Fig. 3B and C). While continued public submission and high-throughput reference sequence generation [85] will increase the representation of missing taxa and loci over time, ensuring the quality of submitted sequences from correctly identified specimens is crucial [24]. There are numerous examples of barcode sequences being either insufficiently annotated [34], annotated with the incorrect species in public databases [81, 86–89], or multiple morpho-species assigned to the same DNA barcode, which may reflect misidentifications or the existence of species complexes [58]. These issues highlight the importance of engaging taxonomic experts to ensure a priori identification of a specimen before submitting a reference barcode to a public database [90, 91]. Furthermore, the use of non-destructive DNA extraction methods when generating barcode sequences would allow the retention of voucher specimens to ensure traceability between the molecular and morphological features, especially in the case of taxonomic reassignments [92].

While some metabarcoding studies have responded to the aforementioned issues by exclusively using in-house reference databases for taxonomic assignment [90, 93–95], because many insect surveillance programmes aim to detect species that are not locally present, the reliance on public data to supplement in-house sequences may be unavoidable. Some taxonomic classifiers used in metabarcoding studies provide the option to weight classifications towards certain reference sequences [96, 97], which could be beneficial when combining high-confidence in-house sequences with public sequences of more variable quality, or when the endemic diversity for the target region is well characterized [74, 98]. Regardless of source, barcode sequences will be compiled together and formatted appropriately for use with automatic taxonomic classification software [99–101], and this presents an ideal point where automated or semiautomated curation methods can be used to identify and remove any taxonomically mislabelled sequences or non-homologous regions such as pseudogenes [74, 102]. Finally, curated databases used in an active surveillance program should only be updated after rigorous testing with standardized datasets to ensure that assay results remain accurate and reproducible following addition of new sequences [103].

### Marker enrichment

Similar to conventional DNA barcoding, most metabarcoding studies use a set of universal oligonucleotide primers to exponentially amplify a target barcode marker until it reaches a concentration appropriate for sequencing. This “amplicon sequencing” methodology has proven reliable and sensitive for detection of low-abundance taxa in bulk samples [40]. However, differential PCR amplification efficiencies between taxa generally result in a biased depiction of relative abundances of community members [104]. This bias is thought to mainly arise from primer-template mismatches, particularly at the 3′ end of the primer where extension takes place [64, 105] and therefore comprehensive *in silico* evaluation should be conducted at the beginning of a project to ensure that primer sequences are appropriate for the underlying target community [106–108]. Where mismatches with certain taxa are predicted to occur, inclusion of degenerate bases can overcome taxonomic bias inherent to a specific primer sequence [109, 110]; however, high levels of degeneracy can also lead to undesirable off-target amplification or formation of dimers [87, 111], which will require further laboratory validation to detect [71, 109, 112]. In addition to the effects of PCR primers, a range of template-specific factors including copy number of the loci [113], nucleotide composition and secondary structure [114], variable amplicon lengths [115], specimen biomass [116], and complexity of the species mixture [105, 117] can further contribute bias. While the cumulative bias from all these factors may suggest that amplicon sequencing can only be used for presence-absence data, importantly, sequencing reads are still correlated with DNA input in a predictable way, and biases should only affect the slope of that correlation [113]. Therefore the calculation of taxon-specific correction factors shows great promise for improving abundance estimates from metabarcoding data [113, 118–120], particularly for simpler communities such as those trapped using targeted attractant lures [17]. Nevertheless, if accurate quantification is essential for the surveillance programme, removing the PCR amplification process altogether should also be considered for improving taxon abundance estimates from metabarcoding data.

### PCR-free approaches

The major alternative to amplicon sequencing–based metabarcoding involves simply fragmenting the genomic DNA extract to lengths appropriate for the sequencing platform and directly sequencing it without any prior bias-inducing enrichment step. This methodology, termed “shotgun metagenomics,” generates sequence reads comprising a random subsample of the mixed community DNA and relies on the higher representation of taxonomically informative multi-copy mitochondria and nuclear rRNA in this subsample to identify community members [121–123]. In addition, these high-copy regions can be assembled into long contigs and even full-length mitochondrial genomes for further phylogenetic inference and systematics applications [124, 125]. Despite this, restricting taxonomic analysis to just mitochondrial and nuclear rRNA regions still leaves the vast majority of reads corresponding to DNA that is not taxonomically informative or easily assembled from a bulk sample to be discarded [121] and deep sequencing will be required to reliably detect rare specimens in the community [125, 126]. While the rapid growth in sequencing capabilities is making this brute force approach to community identification increasingly possible, for routine surveillance a cost-effective method for enriching taxonomically informative loci should be used prior to sequencing. A range of potential methods for PCR-free sequence enrichment have been reviewed elsewhere (see Mamanova et al. [127] and Jones and Good [128]), but some examples that have been successfully used for metabarcoding include differential centrifugation to enrich for mitochondria [129] or baiting target barcode markers and whole mitochondria using hybridization probe capture [130–133]. Hybridization capture relies on the use of thousands of synthetic oligonucleotide probes, each with strict complementarity to a target sequence, and therefore should ideally be designed with a priori knowledge of every target sequence [128]. Although this may be a limiting factor for recovery of previously unsequenced diversity, the flexibility to include essentially infinite numbers of probes provides further advantages for building bespoke metabarcoding assays that capture diverse loci for purposes beyond taxonomic inference (Box 2). Nevertheless, while PCR-free approaches have shown improved correlations between sequencing reads and input DNA [123, 134], it is important to remember that HTS counts molecules not individual specimens [45] and therefore biases are likely to still remain due to variation in biomass and copy number between organisms and tissues [131, 134]. Furthermore, the process of PCR amplification is already widely accepted within diagnostics protocols [49], and implementation of alternative PCR-free sequence enrichment methods may require overcoming additional regulatory hurdles.

Box 2: **Modular metabarcoding assays**

Many of the insect pests actively monitored by surveillance programs are not targeted because of direct damage they do to animals, plants, or the environment but instead the associated fungi, bacteria, viruses, and viroids for which they can be vectors [52, 135, 136]. Similar to identification of insects, detection of host-associated pathogens has previously required screening of trapped samples on a specimen-by-specimen basis using target-specific assays or culturing and morphological analysis [33]; however, this is rapidly being augmented with metabarcoding and metagenomic approaches [33, 103, 137, 138]. The ability of HTS platforms to sequence a heterogenous mix of loci opens up the opportunity for combining both the identification of insects and the screening of a diverse range of host-associated microbiota within a single multiplexed metabarcoding assay [40, 139]. Nonetheless, developing an integrated assay that allows detection and identification of biologically diverse organisms in a diagnostics context presents a number of challenges. Extraction techniques will need to be optimized to account for the pathogen association with its insect host (i.e., intracellular [140], external [141], gut-borne [142]), and specific microbial life histories may make this incompatible with non-destructive DNA extraction. Furthermore, PCR protocols will need to be optimized to account for the large differences in template quantity between abundant host DNA and low-titre vectored organisms [143].

In contrast with the high resolution that COI provides for identification of insects, the commonly used universal markers for bacterial and fungal barcoding struggle to identify organisms to the species or strain level, which is necessary to separate pathovars from common innocuous environmental organisms [33, 136]. Therefore, diagnostic assays that aim to be universal for identification of both host and vectored organisms will require analysis of a range of group-specific markers in multiplex, or make use of long-read HTS platforms for increased taxonomic resolution [144, 145]. While multiplexing many loci together in single PCR reactions can greatly simplify laboratory protocols and therefore costs involved, for metabarcoding this can be complicated by cross-reactivity between primers and individual primer sensitivities changing depending on community composition [76, 105, 112]. As an alternative, various target loci could be enriched in parallel reactions and then pooled together by sample prior to library preparation in proportions relative to the number of reads desired for each marker [40, 146]. This highly flexible modular approach would then allow group-specific microbial primers or other markers of interest to be added or retracted from the assay depending on the target community and needs of the end user. For example, Swift et al. [147] have demonstrated the ability of modular metabarcoding assays not just to identify cross-kingdom species composition but also to genotype microsatellite loci and sex-specific markers relevant to the community under study. While the field of invasion biology has traditionally been concerned with the transport and movement of species, this doctrine overlooks the intraspecific movement of genetic material such as pesticide resistance alleles [148], transposable elements [149], and genetically modified organisms [150]. The ability to capture essentially any loci in a modular metabarcoding assay may allow integration with a more gene-focused model of biosecurity in the future.

### Library preparation and multiplexing

**Figure 4:**
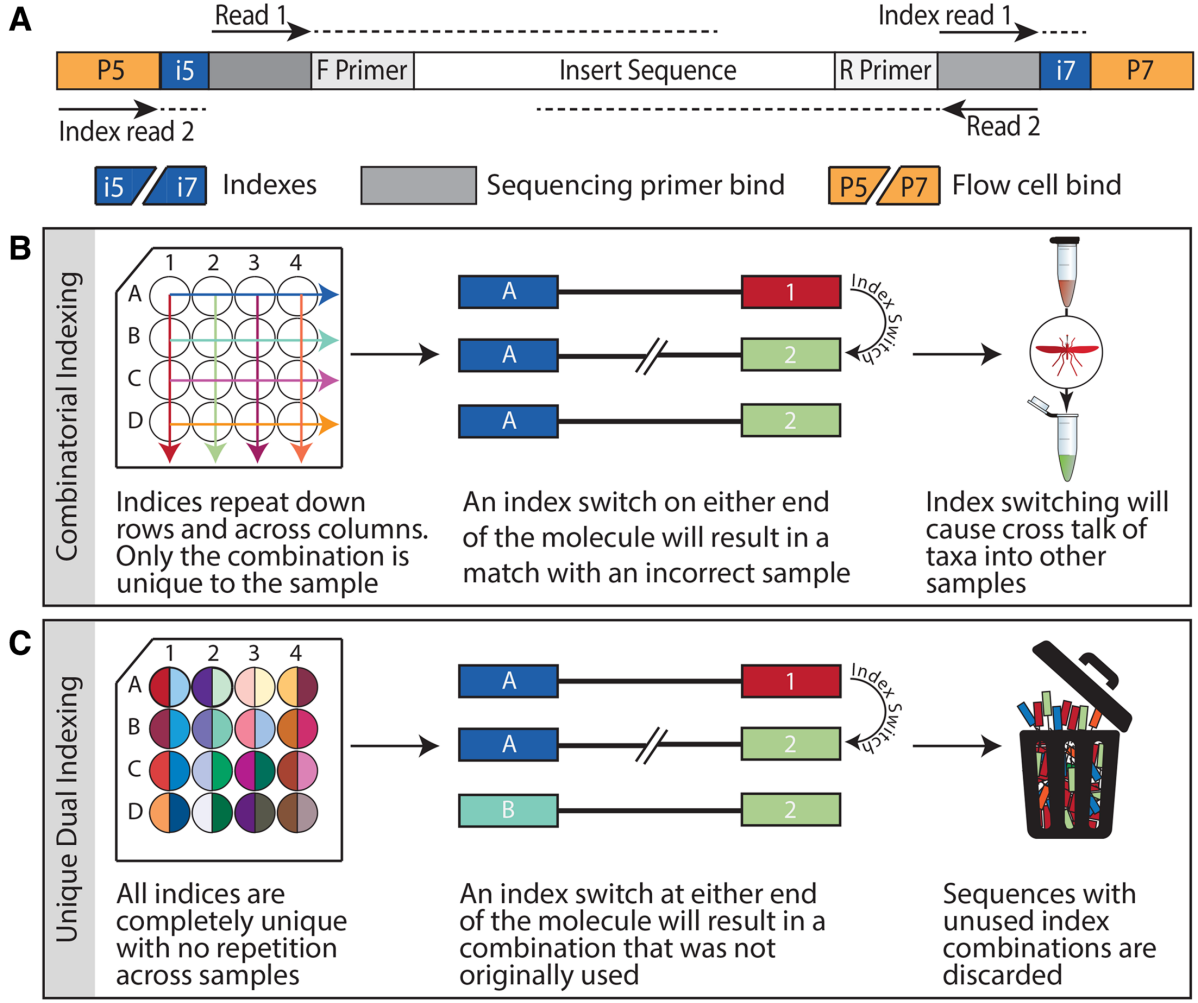
Unique dual indexing overcomes issues of cross-contamination due to index-switching. (A) An amplified barcode locus with sequencing adapters attached; read locations and orientations are indicated for commonly used Illumina MiSeq platform. Reads 1 and 2 are designed to overlap to facilitate assembly into a consensus sequence. Both sequencing adapters incorporate a unique oligonucleotide index sequence to allow differentiation of multiplexed samples. Strategies for indexing include (B) combinatorial indexing, where indices on either end of the molecule are shared with other samples but the combination of the two is unique, and (C) unique dual indexing, where adapter indices at both ends of the molecule are completely unique to the sample.

Regardless of whether an enrichment or metagenomics approach was used, platform-specific sequencing adapters need to be attached to the molecules (via ligation [151] or 1-step [152] or 2-step PCR [40, 106]) to form “libraries” that can then bind to the flow cell for sequencing (Fig. 4A). Because current HTS platforms output sequences far in excess of what is required to identify the taxa in a single community, metabarcoding studies commonly multiplex many samples together on a single flow cell and use oligonucleotide index sequences incorporated into the sequencing adapters to link sequencing reads back to origin sample. While a range of indexing strategies exist for HTS [153], for sensitive diagnostics applications it is critical to choose an approach that can adequately cope with the occasional recombination of these indices between molecules. Index-switching has received particular recent attention due to reports of remarkably high levels on current Illumina platforms [154]; however, similar phenomena can affect multiplexed sequencing across all major platforms to various degrees [155–159] (with the possible exception of recent MGI platforms [160]). Suggested causes include contamination from residual adapter/primer oligonucleotides [161], chimera formation during adapter PCR [162], mixed clusters on the flow cell [157], or physical contamination during library preparation or oligo synthesis by the vendor [159, 163, 164]. Regardless of mechanism, when not properly controlled for, index-switching can cause taxa from one sample to “bleed” into others, and while this will only produce false-positive results for a taxon of concern when a true-positive result is present in ≥1 of the samples, the spreading of positive signal across samples can imply that the taxon of interest has a larger geographic distribution than exists in reality. Recent studies have demonstrated that the most effective method for controlling for index-switching is through the use of unique dual indices (Fig. 4C) rather than the commonly used combinatorial indexing (Fig. 4B). When unique dual indices are used, switching events at either end of the molecule will generate an index combination that was not originally applied and, during de-multiplexing, the reads with mismatched indices to the sample sheet will be filtered into an unassigned-reads file and excluded from analysis [159, 162, 165]. Furthermore, sets of indices should be alternated for each sequencing run [51] because carryover of molecules between runs on an HTS machine can be a further cause of false-positive results in high-sensitivity sequencing applications [166]. Finally, it is important that index sequences used are designed with sufficient edit distance between them so that substitution or insertion/deletion errors within the index do not cause further sequence misassignment [131, 167], particularly for higher error rate platforms such as nanopore [115].

### High-throughput sequencing platforms

While the rapid growth of HTS over the past decade has produced a variety of techniques and chemistries for discerning the nucleotide sequence of a DNA molecule [168], modern platforms can largely be divided into those producing short-but-accurate sequences or long-but-error-prone sequences (Table 2). To date, the majority of metabarcoding studies have been conducted using the former, with the Illumina “MiSeq” dominating the recent metabarcoding literature due to its high-quality reads and relatively inexpensive purchase cost (Fig. 1B). Despite the current popularity of the MiSeq for research studies, the cost per sample may be impractical for the number of specimens produced by large-scale surveillance programmes, and instead the production-scale Illumina “NextSeq,” “HiSeq,” and “NovaSeq” provide progressive increases in throughput and therefore cost reductions (Table 2). Nevertheless this increased sequencing throughput of these platforms must be balanced with diagnostic turnaround times, and effective use of the ultrahigh-capacity HiSeq and NovaSeq flow cells will involve multiplexing of thousands of samples, necessitating substantial logistical efforts in sample collection and processing [103].

Despite the cost-effectiveness of the aforementioned platforms, their restricted read lengths (Table 2) limit the taxonomic resolution achievable with a metabarcoding assay and therefore long-read sequencing platforms such as the Pacific Biosciences (PacBio) “Sequel” and Oxford Nanopore Technologies (ONT) “MinION” and “PromethION” are becoming increasingly attractive alternatives. The ability to sequence barcode regions thousands of bases in length has potential to enable greater recovery of taxonomic diversity with intraspecific resolution [169]; however, in practice the utility of long reads for species identification has been limited by considerably higher per-base error rates that commonly exceed intraspecific distance [115, 170]. Nevertheless, methods for repeatedly sequencing a single molecule to create higher quality consensus sequences [171] are now opening up applications in metabarcoding [144, 158], with natively implemented circular consensus sequencing on the PacBio Sequel producing consensus reads with similar accuracy to traditional Sanger sequencing [172], and third-party protocols mimicking this approach have now been published for the ONT platforms [173, 174]. If similarly robust consensus sequencing can be achieved with nanopore technology, the significantly smaller start-up cost and portability of the handheld MinION platform may in future permit metabarcoding-based diagnostics to be conducted in remote field sites [115], as well as enable lesser resourced laboratories to access these technologies [14].

### Bioinformatics

Computational processing of sequence reads represents a series of steps of equal importance to laboratory protocols for ensuring accurate and sensitive detection of invasive species [175, 176]; however, many of the skills and techniques involved in this process have not historically been required within diagnostic laboratories. While there exist a number of popular end-to-end computational pipelines for analysing marker gene data [177–181], many of these have been designed for measuring diversity rather than detection of low-abundance taxa. Each step in the bioinformatic analysis can present trade-offs between sensitivity to rare taxa, amount of erroneous sequences retained, and overall computing time [77, 175, 182–184], and use of metabarcoding in an invasive species surveillance or other sensitive context presents some unique challenges and regulatory requirements that may be best addressed through the creation of a custom analysis pipeline [146, 176].

#### De-multiplexing and sequence quality trimming

A metabarcoding assay typically involves multiplexing many samples into a single pooled sequencing library in order to make optimal use of the high-capacity flow cells of current sequencing platforms. Therefore, the first step following sequencing (typically automated by the HTS platform's software) is to assign sequences back to their origin sample using unique oligonucleotide sample indices incorporated into the sequencing adapters (Fig. 4). Following de-multiplexing, sequencing adapters and any other non-biological information such as PCR primer sequences are removed, and reads are assembled into consensus sequences using their overlapping bases. While improvements in underlying sequencing chemistries and aforementioned consensus approaches means that the majority of platforms now provide per base accuracies >99.99% (with the notable exception of nanopore platforms) [168, 173, 185], when put in context of the billions of bases sequenced on modern flow cells, tens of thousands of sequences will still contain errors [186]. Raw sequence reads are generated in conjunction with a predicted error profile based on signal intensity and background noise, and these data are generally presented to the user in the form of a FASTQ file [187]. An initial quality-trimming stage uses this profile to truncate or remove sequences that contain excessive ambiguous or low-confidence base calls [186, 188]; this is, however, a coarse filtering process where parameters should be carefully considered, particularly for higher error platforms such as nanopore. While strict quality trimming will more effectively remove sequencing artefacts and erroneous reads that can affect downstream diversity and abundance estimates, overly conservative parameters can result in removal of too many reads and therefore loss of sensitivity to low-abundance taxa [146, 176].

#### OTU clustering and denoising

While quality trimming can improve accuracy by removing sequencing errors, the PCR amplification process used in the majority of metabarcoding studies can further introduce single-base substitutions [158, 189] and length variation [190] that will not necessarily be associated with low quality scores [191]. Because these noisy sequences can cause spurious results and substantially increase downstream computation, many studies cluster together all sequences within an arbitrary similarity threshold (commonly 97%) into representative bins called “operational taxonomic units” (OTUs). While the 97% similarity threshold is thought to represent a broadly generalizable compromise between interspecific and intraspecific variation and is commonly used to indicate distinct taxa [192, 193], actual coalescent depths between species can differ greatly across taxonomic groups [91]. Therefore when a single global threshold is applied to diverse communities it can result in both the splitting of a single species across multiple OTUs, as well as the lumping of multiple species into the same OTU, resulting in false-negative results [176, 194]. Furthermore, aggregating all similar sequences into a single OTU loses all information on intraspecific diversity, restricting the ability to trace the geographic origin of invasive populations [39, 79]. In addition, the OTUs generated by clustering are dependent on the particular dataset, reference database, and parameters selected [194, 195], and as such they do not lend themselves to ongoing comparison with the constantly evolving data produced by a longitudinal surveillance programme. To overcome the aforementioned limitations, newly developed “denoising” algorithms instead use statistical models to infer true biological sequences from sequencing noise and correct for single-nucleotide differences, without imposing the arbitrary similarity threshold that defines OTUs [196–198]. This single-nucleotide resolution enables binning sequences into “amplicon sequence variants” (ASVs) [196] (also termed “exact sequence variants” [194], sub-OTUs [197], or zero-radius OTUs [zOTUs] [198]) that retain precise haplotype information that can be necessary for diagnostics of closely related taxa or tracking an invasion [199], and act as a consistent label between analyses [194].

#### OTU quality control

While the above measures account for the majority of low-abundance errors, they are not designed to deal with high-abundance artefacts such as PCR-generated chimeras and non-specific amplification products. Chimeric sequences are the result of incompletely extended PCR products acting as primers for a different closely related sequence [189], and therefore appear as concatenated products of 2 parent sequences. Assuming that parent sequences will be more abundant having undergone more rounds of amplification, chimeras can be algorithmically removed through comparison with other sequences in the sample [196, 200] or with a chimera-free reference database [201]. On the other hand, removing products of non-specific amplification such as intragenomic variants and pseudogenes presents more of a challenge and will generally require manual curation [151, 202]. When targeting protein-coding mitochondrial genes such as COI, the presence of stop codons and frameshifts that disrupt the open reading frame are common indicators of pseudogenes [80], and for rRNA markers secondary structure prediction could be used to ensure that sequences do not contain substantial variation in highly conserved regions [203]. Because it is inefficient to include a manual curation process as part of a high-throughput bioinformatics pipeline, it would be beneficial for future denoising algorithms to incorporate patterns of sequence evolution to allow more precise and automated filtering of barcode loci from erroneous and pseudogenic sequences [80, 204, 205].

#### Taxonomic assignment

In order to process the large diversity of sequences that a metabarcoding assay typically produces, the assignment of Linnaean taxonomy (e.g., species, genus) is typically conducted in an automated manner. While a large range of software tools exist for this purpose [206], the approaches used can generally be delineated into either sequence similarity searches (i.e., BLAST alignment), sequence composition methods (i.e., hidden Markov models and *k*-mer counts), phylogenetic methods, or a hybrid of the above (see Bazinet and Cummings [207] for an in-depth comparison). To date, the most widely used approach for taxonomic classification in metabarcoding studies has been best-hit classification using alignment based tools such as BLAST [208], which assume that the taxonomy of the query sequence will be identical to the taxonomy of the most similar sequence in a reference database. While this approach is simple to implement and can perform effectively when the reference database contains sequence information from conspecifics, when reference data are absent or when the particular loci cannot distinguish between multiple organisms, best-hit classification is prone to over-classifying the sequence to incorrect species-level taxonomy [209]. In the worst case, this over-classification error could lead to false-positive results by classifying a previously unsequenced but probably innocuous organism as a known pest, owing to the pest being the closest taxon with an existing reference sequence [210].

As the above situation demonstrates, for applications where management decisions are to be based on the results of a taxonomic classification, a central question is the reliability of that classification. A number of taxonomic assignment algorithms aim to address this issue by returning a measure of confidence of inclusion in each taxonomic rank, e.g., by using repeated random sampling [97, 211], lowest common ancestor methods [212], or probabilistic models [96, 213]. In an ideal case, only a single possible taxonomic outcome will obtain a high level of confidence, whereas alternate outcomes will obtain probabilities close to zero. In cases where there may be uncertainty at the species or genus level due to imperfect reference data and multiple taxonomic outcomes obtaining similar probabilities, the sequence may still be robustly assigned to a higher taxonomic rank (e.g., family) [101], providing important information about sample composition and possible presence of novel taxa without producing false-positive results [214]. While using measures of confidence can reduce the incidence of over-classification, many of these approaches are impaired by an inherent bias in that they infer the entire scope of possible taxonomic outcomes exclusively from the reference sequences used for training [215, 216], which in reality only represents taxonomic units that have been previously sequenced. In contrast, the Bayesian framework of PROTAX [96] accepts a reference taxonomy tree alongside the reference sequence database in order to account for taxa that are present in Linnaean taxonomy but not represented by reference sequences. Furthermore, PROTAX explicitly models the probability that a sequence belongs to a taxon that is novel to both the reference sequence database and reference taxonomy, which could be particularly important when conducting surveillance in regions with substantial uncharacterized biodiversity [216, 217]. Nevertheless, even the most complex taxonomic assignment algorithms do not model important aspects of species biology that may limit the possible geographical distribution or habitat in which they could reasonably exist, and therefore the results of taxonomic assignment should be vetted with ecological knowledge of the detected species where possible [35].

#### Quality assurance

The ability to simultaneously identify many loci from thousands of specimens in a single diagnostic assay underlies the power of the metabarcoding approach to surveillance; however, the resulting increase in sequence diversity and analytical complexity introduces further risk of cross-contamination and technical error [55]. An important challenge for the use of metabarcoding in a diagnostic context is the rate of false-positive errors (incorrect identification of an insect as the pest of concern) and false-negative errors (not identifying a pest of concern). While many ecological studies prioritize minimizing false-positive errors over false-negative errors [37], generally the precautionary principle applies in biosecurity; i.e., it is better to have a false-positive result that can be followed up with an orthologous confirmation method than to miss a serious pest. This is particularly important if the assay is to provide “evidence of absence” to support pest-free status [218], which can be required to access certain international markets [28]. Therefore, a quality assurance system for metabarcoding diagnostics should aim to reduce the frequency of false-positive results as much as possible through the appropriate use of controls, replication, and validation, without in turn increasing the incidence of false-negative results.

#### Controls and replication

The majority of contamination in next-generation sequencing assays is expected to arise from other samples processed in the same laboratory environment, particularly when PCR is involved [164, 219], and therefore workspaces should be physically or temporally separated for different assay steps, with all surfaces, equipment, and reagents regularly decontaminated [33, 219–221]. Periodic swipe tests of laboratory surfaces can help identify common laboratory contaminants and confirm the absence of environmental DNA from target pests [220, 222]. Despite these precautions, even the cleanest laboratory environment will not account for all possible contaminant sequences and therefore no-template controls should be included throughout the entire laboratory workflow and sequenced alongside the sample libraries to provide a cumulative measure of contamination [162, 223, 224]. When these controls are incorporated sequentially at each step of the laboratory protocol they can further enable partitioning of contamination to the stage in the workflow where it occurred, which can highlight processes that can be improved during assay development [35, 37]. Index-switching is perhaps the most worrisome cause of contaminating sequences in HTS, and while use of unique dual indices (Fig. 4C) can reduce this phenomenon to a level acceptable for most studies, trace levels of index-switching can still persist and cause issues for sensitive diagnostic applications [159]. While index-switching artefacts will be detectable in no-template controls, it can be difficult to discern this phenomenon from sequences arising through physical contamination. Instead, including a positive control library made up of synthetic standard DNA [177, 225, 226] or an “alien” taxon guaranteed to be absent from the sample [88, 227] allows empirical measurement of the index-switch rate. Alternatively, the rate of index-switching can be measured post hoc by comparing read counts between valid and invalid combinations of unique dual indices [131, 228]. Once contaminant sequences have been identified, their presence can be controlled through the application of a minimum abundance filter based on the read counts within negative and/or positive control libraries [35, 229], although choice of an appropriate threshold can be complicated by read depth differences between samples and preferential amplification of contaminants in low-biomass no-template control samples [175, 230]. As an alternative, new statistical methods allow systematic removal of contaminant sequences based on co-occurrence patterns and library quantification data [231–233]; however, if particularly high levels of contamination or abnormally high rates of index-switching are detected in a specific batch of samples, it may be more appropriate to repeat the assay. Finally, including an additional positive control in the form of a well-characterized mock “calibration community” in every sequencing run could further highlight any additional run-specific aberrations or batch effects that may have been introduced during the metabarcoding workflow when taxonomic composition or error rates deviate strongly from expected [205, 234, 235].

In addition to being prone to contamination, library preparation protocols involve a series of molecular bottlenecks where during each subsequent stage of DNA extraction, target enrichment, and binding of molecules onto the flow cell, only a random subsample of molecules are taken forward [37]. Stochasticity in this sampling process is likely to bias the resulting sequences towards more abundant taxa and increase the false-negative rate for rare taxa [236], and this can be further exacerbated by negative primer bias [77]. Potential loss of rare taxa during sample processing can be offset through the use of technical replicates, and these provide a further avenue to identify laboratory cross-contamination in the case that replicates show significant dissimilarities in taxonomic composition [77, 229, 237]. While using higher numbers of replicates can increase the probability of detecting rare taxa [237], this must be weighed against the increased costs of sequencing and library replication as well as the strategy for processing the replicates [37]. Additive processing (i.e., pooling the detections of all replicates) can be most useful for overcoming sampling stochasticity and controlling for false-negative results, while restrictive processing (i.e., only retaining sequences present in several replicates) more effectively controls for cross-contamination. To balance the positives of both approaches, it may be best to include a minimum number of technical replicates to allow a majority-rules approach (e.g., 2/3 replicates count as a detection) [77, 88, 112]. A further aspect to consider is the importance of biological replicates at the sample collection stage [238] because regardless of the effectiveness of the metabarcoding diagnostic assay, if an insect is not caught in a trap, it does not necessarily mean absence in the area. The use of site occupancy models that account for the false-positive– and false-negative–prone nature of metabarcoding surveys could be used to determine the optimal number of both technical and biological replicates to reach the desired statistical power for the survey [239, 240]. Finally, while outside the scope of this review, appropriate trap design [241] and surveillance grid planning [242] must also be adhered to for effective metabarcoding-based surveillance.

#### Validating metabarcoding assays

Because of the relevance of many invasive insects to international trade and human health, laboratories conducting insect diagnostics generally exist within strict regulatory environments. As part of laboratory accreditations, newly developed assays are required to undergo a validation process in order to provide objective evidence to all end users that an assay is fit for purpose [53, 54, 243, 244]. Traditionally, validation first involves defining the scope of the assay and then establishing performance parameters such as analytical sensitivity, analytical specificity, reproducibility and repeatability for every individual target designated in this scope [26, 244, 245]. However, the universal nature of metabarcoding assays and the taxonomic diversity of potential surveillance catch make this impractical [246]. To overcome this inevitable variation between reference samples and reality, a flexible scope validation process should be used to establish performance parameters on representative samples and identify critical steps in the workflow where variation can be introduced [146, 247]. These critical steps can then be monitored run to run using control samples and appropriate quality control checkpoints (Table 3) to ensure that no sample or sequence data continue without meeting minimum quality requirements [51, 221, 247, 248]. In the case of insect metabarcoding, mock communities made up of the taxonomic groups of interest are generally used for validation, which are then spiked with decreasing concentrations of target species in order to establish assay sensitivity and limits of detection [40, 249]. Because DNA extraction efficiency and primer bias can be affected by overall community complexity [105, 250], mock communities should as closely as possible represent the diversity expected to be recovered in different trapping scenarios. Furthermore, the amount of sequencing effort assigned to an individual sample during multiplexed sequencing can vary across runs [224, 251], and the effect of sequencing depth on detection should also be established using rarefaction curves [107, 117]. On the other hand, analytical specificity will generally depend on choices made during assay design, such as the choice of target marker, availability of appropriately annotated reference sequences for the chosen marker, and taxonomic assignment criteria used [220, 246]. Parameters such as precision and reproducibility of a metabarcoding assay can be established similar to other molecular diagnostics, through replication of samples and controls within and across sequencing runs and inter-laboratory comparisons [146]. Finally, stability of specimens and DNA to environmental factors such as temperature, UV radiation, pH of commonly used drowning or attractant solutions (e.g., vinegar traps [252]), and exposure to environmental microorganisms in the field and during storage [253] should be evaluated and may prompt a need for redesign of insect traps to collect and preserve samples in a manner more suited to DNA-based identification.

**Table 3:** Recommended quality control checkpoints for metabarcoding-based diagnostics

Category	Quality control checkpoint	Consequences
Laboratory preparedness	Are all reagents within expiry date and stored properly?	Poor reagent storage can lead to reduced efficiency and false-negative results
	Is equipment appropriately maintained and calibrated?	Poorly calibrated equipment will generate inconstancies and inaccurate data
	Have laboratory surfaces been decontaminated and swipe testing of laboratory surfaces been conducted?	Dirty laboratories can be a source of DNA contamination, leading to lowered sensitivity or false-positive results
Sample acceptance	Have specimens arrived in a condition appropriate for extracting DNA?	Inappropriately stored specimens can lead to false-negative results and a reduction in sensitivity
	Are specimens traceable to origin location?	Misidentification of sample origin can complicate detection response
Nucleic acid extraction	Is DNA of sufficient quantity and quality?	Insufficient DNA quantity or presence of contaminants can inhibit reactions and result in false-negative results
Marker enrichment	Are the correct fragment sizes present for the target barcode marker?	Incorrect fragment sizes could indicate off-target amplification
	Have the positive control samples successfully amplified?	Absence of product in positive controls indicates amplification failure
	Are negative control samples free of DNA fragments?	Visible DNA fragments in negative controls indicates contamination
Library preparation and multiplexing	Are libraries of the appropriate size and concentration?	Libraries of significantly different sizes or concentrations will complicate multiplexing
	Have sets of unique dual indices been used?	Unique dual indexing is necessary to control for index-switching
	Have index sets been alternated since the previous sequencing run?	Cross-contamination of libraries between sequencing runs can cause false-positive results
High-throughput sequencing	Has the pooled library been appropriately sized and quantified?	Inaccurate sizing and quantification can cause overloading of flow cell and failed runs, or underloading and low data output
	Has the sequencer been appropriately cleaned between runs?	Insufficient cleaning of the sequencer can result in cross-contamination between runs
De-multiplexing and quality trimming	Has minimum sequencing depth been achieved for each sample?	Low sequencing depth can cause false-negative results
	Are an appropriate number of reads passing quality filtering?	Low numbers of reads passing quality filters can indicate issues with sequencing run and result in false-negative results
OTU clustering and denoising	How much of the original data are explained by the final OTUs/ASVs	Lower-than-expected sequences can indicate overly restrictive bioinformatics parameters
	Have chimeras and sequences with disrupted open reading frames been checked for? (for protein coding genes)	Chimeras and pseudogenes can inflate taxonomic diversity, leading to false-positive results
Taxonomic assignment	Has the reference database been curated to remove mislabelled taxonomy and pseudogenic sequences?	Mislabelled reference sequences can lead to both false-positive and false-negative results
	Has the taxonomy been applied with appropriate confidence levels?	Low-confidence assignment indicates incomplete or erroneous reference database
Interpretation of results	Have the taxa received an appropriate number of reads to pass detection threshold?	Taxa under detection threshold could represent laboratory or reagent contamination, or erroneous sequences that have not been sufficiently controlled for
	Has a minimum detection threshold been applied to remove index-switching?	Index-switching can cause spreading of taxa to other samples and result in false-positive results
	Are there any taxa that need to be confirmed with alternative methods?	Any high-risk putative detections should be confirmed with alternative method before reporting, if possible
Reporting and sign-off	Have any exceptions to laboratory standard operating procedure been made?	Non-compliances with standard operating procedure should be highlighted, and diagnostic confidence may be reduced
	Have data been stored appropriately?	Archiving of data allows future re-analysis in case of disputed results
	Have results been signed off by competent individual?	Incorrect reporting or interpretation of significant taxa can lead to incorrect managment response

#### Reporting and confirming detections

Even when primers are designed around a specific taxonomic group, metabarcoding can amplify and detect many more taxa outside the scope of the original validated target list [254]. How these incidental detections are reported and eventually acted upon will present a major challenge to diagnostic laboratories and end users, due to the increased number of previously undocumented taxa being discovered for which knowledge of distribution or ecological significance may be missing [51, 53]. Many of these incidental detections will be taxa that simply have not previously been searched for, and when an appropriate management response is considered, it will be important not to conflate “first detection” in an invasion biology sense, where there was prior evidence of absence, with merely the first time a species has been formally identified in a region [255]. Hence a greater emphasis needs to be placed on conducting baseline surveys to establish comprehensive species checklists of endemic diversity and resolve synonymous taxa at the beginning of a surveillance programme to avoid creating sudden market access and trade issues [256]. Furthermore, a decision framework should be developed for evaluating incidental detections that sets out steps for further characterization and risk assessment for the detected organisms in order to establish whether eradication or other management actions are appropriate or achievable [257]. Where necessary, putative detections can be further confirmed using an orthogonal diagnostic method such as quantitative PCR/droplet digital PCR on the original DNA extract [146]; however, these assays require prior development and will therefore not be available for all incidental taxa detected in a metabarcoding assay. Instead, the use of non-destructive DNA extraction methods that use a combination of enzymes, buffers, and heat without mechanical homogenization [227, 258–260], or even amplification of insect DNA from the ethanol used to preserve specimens [261–264], would enable diagnosticians to revisit original samples following metabarcoding to confirm species detections. Development of a non-destructive metabarcoding assay has great potential for bridging the gap between new HTS methods and traditional entomological techniques and may bootstrap the acceptance of metabarcoding into international regulatory frameworks.

### Perspectives and conclusions

The ability to accurately, rapidly, and cost-effectively determine the species composition of bulk insect trap contents using metabarcoding has the potential to revolutionize broad-spectrum surveillance for invasive insect pests. Similar to any novel technology, as metabarcoding transitions from purely research to management applications it faces the growing pains that come with integration into established regulatory structures. While rigorous standardization of both laboratory techniques and data analysis has proven essential for the acceptance of conventional DNA barcoding as a validated diagnostic for insects of regulatory concern [26, 79], the sheer pace of development of HTS technologies and platforms may complicate similar standardization of metabarcoding protocols. Historically, the effective lifespan of many HTS platforms has only amounted to a few years before obsolescence [168], and laboratory protocols and bioinformatic methods are therefore constantly evolving to chase this moving target. In response to this constantly shifting state of the art, harmonization efforts by regulatory bodies should avoid the over-prescription of restrictive standards into law because these will quickly become outdated and risk further widening the gap between research and diagnostics capabilities [46]. Instead, development and distribution of certified reference materials in the form of standard and diverse mock communities or DNA standards (similar to the ZymoBIOMICS microbial mock community standards [265]) as well as computational datasets [266] would enable benchmarking of laboratory and computational methods and begin to characterize the sources of technical variation between laboratories [267, 268]. This could be further developed into an inter-laboratory proficiency testing program where blinded reference samples are periodically distributed for analysis, in order to demonstrate to all stakeholders that an assay is fit for purpose for detecting invasive insect species [248, 269]. The results of these processes would allow further development of best-practice technical guidelines and begin to harmonize approaches across the wider metabarcoding community [270].

Biosecurity and pest management decision making is still largely reliant on the application of a species name to a specimen barcode sequence [81], and issues of mislabelled sequences in public reference databases (Box 1) highlight the importance of maintaining expertise in taxonomy and classical diagnostics to complement high-throughput approaches. Owing to the incomplete nature of reference databases, much of the sequence data currently produced by metabarcoding assays will consist of insufficiently identified sequences [84]. While some of these will no doubt be the result of sequencing errors making it through quality control, many more will represent real taxa and reflect the further work required to more completely describe and acquire reference data for insect biodiversity. Monitoring programs for biological invasions are at their most informative when they are continuous and long term [271, 272], and it would be beneficial for these insufficiently identified sequences to be integrated into reference databases and tracked across analyses and timepoints. Porter and Hajibabaei [84] have highlighted the advantages that ASVs provide over more traditional OTU methods for consistent labelling of insufficiently identified sequences, and embracing non-destructive DNA extraction techniques would further enable taxonomists to verify these sequences using morphological methods and potentially locate previously unbarcoded taxa or novel species, which could then feed back into reference databases [259]. Conventional DNA barcoding and morphological taxonomy currently benefit from a close and reciprocal interaction [273], and we envision a similar relationship for the future of insect metabarcoding. This ability to systematically reanalyse historical datasets with improved reference databases, bioinformatic tools, and biological knowledge presents a major strength of HTS diagnostics [51], and therefore raw datasets should also be archived alongside relevant technical and environmental metadata in a machine-readable format [195]. However the datasets from ongoing longitudinal surveillance quickly amount to terabytes of data [274], the storage, management, and securing of which will require dedicated infrastructure and personnel [53]. Unlike the current drive for open sharing of data in academic research, concerns of misuse harming the international movement of goods means that historically the release of raw diagnostic data to the public has not been common [51]. However, a pathway for declassifying and releasing these data to researchers should be developed because the mass of community-level information generated by metabarcoding bio-surveillance shows great potential for generating new insights into the process and impacts of biological invasion [275].

In an increasingly globalized world, more effective and scalable utilization of surveillance effort will be required to manage the spread and establishment of invasive species. While broad-spectrum approaches to surveillance have historically been limited by the overwhelming amount of diagnostics work generated, metabarcoding-based diagnostics fundamentally change this dynamic by allowing entire communities of diverse organisms containing target pests, endemic species, and unexpected invaders to be simultaneously identified [41]. While present costs of technological investments may currently limit the uptake of HTS tools to only well-funded core diagnostic laboratories, we expect that developments in portable real-time sequencing will further enhance the availability of these tools to a much wider user-base worldwide. Furthermore, it is conceivable that the ongoing miniaturization of sequencers may synergize with advances in microfluidic and lab-on-a-chip technologies [276] to produce a new generation of metabarcoding-based “smart traps” for remote monitoring [277, 278]. Nevertheless, metabarcoding forms just a single component of a larger biosecurity toolbox that contains not only fast, cost-effective, and reliable means of diagnostics but also predictive models, improved risk forecasting, field-tested tools, and an overarching decision support system [46, 52, 135, 137]. The future of biosecurity surveillance and pest management is a distinctly interdisciplinary area, and we encourage future research to involve closer collaboration between academic scientists, diagnosticians, and the end users who rely on effective surveillance data to manage the spread of invasive pests and pathogens.

### Methods

All articles containing "Metabarcoding" in their abstract, title, or keywords were retrieved from the Scopus, PubMed, and Crossref citation databases on 20 June 2019 using the rscopus [279], rentrez [280], and fulltext [281] packages in R 3.5.3 [282]. Duplicated article entries were detected using fuzzy string matching functions from tidystringdist [283], and filtered out using dplyr [284]. All articles containing keywords in their title or abstract indicative of invasive species or sequencing platform used (see supplementary table 1 for full list of keywords) were then represented graphically by year of publication using ggplot2 [285]. A list of global insect pests was then retrieved from Ashfaq et al. [58] and combined with additional pests of concern for Australia [286]. This list was filtered to retain only unique and complete genus species binomials, retaining 558 species, for which all records for these species and the entire Insecta were retrieved from BOLD using the bold package [287]. The list of genes successfully retrieved from BOLD used to query GenBank and all records for species on the pest list and the entire Insecta were retrieved using the Rentrez R package [280]. Records from all databases were combined and specimen collection information was extracted using R and the biofiles package [288]. Of the 5,589,069 records for all loci in the datasets, 4,603,488 were annotated with latitude and longitude information and these were plotted on a world map using ggmap [289]. The number of overall records and unique species within all datasets were then plotted for the top 10 occurring loci.

## Availability of supporting data and materials

A snapshot of the datasets and R markdown documents implementing the analyses contained in this manuscript are available in the Zenodo repository [290].

## Additional files

**Supplementary table 1:**Keywords used to filter articles

**Supplementary information 1:** Reproducable R code used to conduct analyses and produce figure 1

**Supplementary information 2:** Reproducable R code used to conduct analyses and produce figure 3

giz092_GIGA-D-19-00011_Original_Submission

giz092_GIGA-D-19-00011_Revision_1

giz092_Response_to_Reviewer_Comments_Original_Submission

giz092_Reviewer_1_Report_Original_Submission Antton Alberdi Estibaritz -- 3/22/2019 Reviewed

giz092_Reviewer_2_Report_Original_Submission Jose Hleap -- 4/8/2019 Reviewed

giz092_Reviewer_2_Report_Revision_1 Jose Hleap -- 7/8/2019 Reviewed

giz092_Supplemental_File

## Abbreviations

ASV: amplicon sequence variant; BLAST: Basic Local Alignment Search Tool; BOLD: Barcode of Life Data System; bp: base pairs; COI: cytochrome oxidase I; Gb: gigabase pairs; HTS: high-throughput sequencing; IPPC: International Plant Protection Convention; kb: kilobase pairs; Mb: megabase pairs; NCBI: National Center for Biotechnology Information; OIE: World Organisation of Animal Health; ONT: Oxford Nanopore Technologies; OTU: operational taxonomic unit; PacBio: Pacific Biosciences; rRNA: ribosomal RNA; SPS: World Trade Organisation Agreement on the Application of Sanitary and Phytosanitary measures; zOTU: zero-radius operational taxonomic unit.

## Competing interests

The authors declare that they have no competing interests.

## Funding

This work was supported by Horticulture Innovation Australia (ST16010) through funding from the Australian Government Department of Agriculture as part of its Rural R&D for Profit program and Grains Research and Development Corporation. Additional funding was provided by the Plant Biosecurity Cooperative Research Centre(PBCRC No. 2153), and Agriculture Victoria's Improved Market Access for Horticulture programme(CMI105584). A.M.P. and J.B. were further supported by an Australian Government Research Training Program Scholarship.

## Authors’ contributions

A.M.P. and M.J.B. conceptualized the manuscript. A.M.P. drafted the manuscript with contributions from J.B., J.W., J.P.C., N.O.I.C., B.C.R., and M.J.B. All authors read and approved the final manuscript.
