# Supplementary material for: Prospects and challenges of implementing DNA metabarcoding for high-throughput insect surveillance
Source: Gigascience. 2019 Jul 30;8(8):giz092. doi: 10.1093/gigascience/giz092 (PMC6667344; doi:10.1093/gigascience/giz092)
Supplement: giz092_GIGA-D-19-00011_Original_Submission [file giz092_giga-d-19-00011_original_submission.pdf]

GigaScience

Prospects and challenges of implementing DNA metabarcoding for high-throughput insect surveillance

--Manuscript Draft--

**Manuscript Number:**	GIGA-D-19-00011
**Full Title:**	Prospects and challenges of implementing DNA metabarcoding for high-throughput insect surveillance
**Article Type:**	Review
**Funding Information:**	Plant Biosecurity Cooperative Research Centre (2153) — Dr John Paul Cunningham<br>State Government of Victoria (CMI105584) — Dr Mark J. Blacket<br>Horticulture Innovation Australia (ST16010) — Dr Brendan C. Rodoni
**Abstract:**	Trap based surveillance strategies are widely employed for monitoring of invasive insect species, aiming to detect newly arrived exotic taxa as well as track the population levels of established or endemic pests. Where these surveillance traps have low specificity and capture non-target endemic species in excess of the target pests, the need for extensive specimen sorting and identification creates a major diagnostic bottleneck. While the recent development of standardised molecular diagnostics has partly alleviated this requirement; the single specimen-single reaction nature of these methods does not readily scale to the sheer number of insects trapped in surveillance programmes. Consequently, target lists are often restricted to a few high-priority pests, allowing unanticipated species to avoid detection and potentially establish populations.<br><br>DNA metabarcoding has recently emerged as a method for conducting simultaneous, multi-species identification of complex mixed communities, and may lend itself ideally to rapid diagnostics of bulk insect trap samples. Moreover, the high-throughput nature of recent sequencing platforms could enable the multiplexing of hundreds of diverse trap samples on a single flow cell, thereby providing the means to dramatically scale-up insect surveillance in terms of both the quantity of traps that can be processed concurrently, and number of pest species that can be targeted. In this review of the metabarcoding literature, we explore how DNA metabarcoding could be tailored to the detection of invasive insects in a surveillance context and highlight the unique technical and regulatory challenges that must be considered when implementing high-throughput sequencing technologies into sensitive diagnostic applications.
**Corresponding Author:**	Alexander M Piper, B.Sc.<br>La Trobe University<br>Bundoora, VIC AUSTRALIA
**Corresponding Author Secondary Information:**	
**Corresponding Author's Institution:**	La Trobe University
**Corresponding Author's Secondary Institution:**	
**First Author:**	Alexander M Piper, B.Sc.
**First Author Secondary Information:**	
**Order of Authors:**	Alexander M Piper, B.Sc.<br>Jana Batovska, B.Sc. (Hons)<br>Noel O.I. Cogan, PhD<br>John Weiss, PhD<br>John Paul Cunningham, PhD

Powered by Editorial Manager® and ProduXion Manager® from Aries Systems Corporation

	Brendan C. Rodoni, PhD
	Mark J. Blacket, PhD
**Order of Authors Secondary Information:**	
**Additional Information:**	
**Question**	**Response**
Are you submitting this manuscript to a special series or article collection?	No
**Experimental design and statistics**<br><br>Full details of the experimental design and statistical methods used should be given in the Methods section, as detailed in our Minimum Standards Reporting Checklist. Information essential to interpreting the data presented should be made available in the figure legends.<br><br>Have you included all the information requested in your manuscript?	Yes
**Resources**<br><br>A description of all resources used, including antibodies, cell lines, animals and software tools, with enough information to allow them to be uniquely identified, should be included in the Methods section. Authors are strongly encouraged to cite Research Resource Identifiers (RRIDs) for antibodies, model organisms and tools, where possible.<br><br>Have you included the information requested as detailed in our Minimum Standards Reporting Checklist?	Yes
**Availability of data and materials**<br><br>All datasets and code on which the conclusions of the paper rely must be either included in your submission or deposited in publicly available repositories (where available and ethically appropriate), referencing such data using	Yes

a unique identifier in the references and in the “Availability of Data and Materials” section of your manuscript.<br><br>Have you have met the above requirement as detailed in our Minimum Standards Reporting Checklist?	

Manuscript
Click here to download Manuscript DNA_Metabarcoding_Insect_Surveillance.docx

Prospects and challenges of implementing DNA metabarcoding for high-throughput insect surveillance

Alexander M. Piper[1,2], Jana Batovska[1,2], Noel O.I. Cogan[1,2], John Weiss[1], John Paul Cunningham[1], Brendan C. Rodoni[1,2], Mark J. Blacket[1]

[1] Agriculture Victoria Research, AgriBio Centre, Bundoora 3083, Victoria, Australia

[2] School of Applied Systems Biology, La Trobe University, Bundoora 3083, Victoria, Australia

Corresponding author:

Alexander M. Piper

Email: alexander.piper@ecodev.vic.gov.au

Abstract

Trap based surveillance strategies are widely employed for monitoring of invasive insect species, aiming to detect newly arrived exotic taxa as well as track the population levels of established or endemic pests. Where these surveillance traps have low specificity and capture non-target endemic species in excess of the target pests, the need for extensive specimen sorting and identification creates a major diagnostic bottleneck. While the recent development of standardised molecular diagnostics has partly alleviated this requirement; the single specimen-single reaction nature of these methods does not readily scale to the sheer number of insects trapped in surveillance programmes. Consequently, target lists are often restricted to a few high-priority pests, allowing unanticipated species to avoid detection and potentially establish populations.

DNA metabarcoding has recently emerged as a method for conducting simultaneous, multi-species identification of complex mixed communities, and may lend itself ideally to rapid diagnostics of bulk insect trap samples. Moreover, the high-throughput nature of recent sequencing platforms could enable the multiplexing of hundreds of diverse trap samples on a single flow cell, thereby providing the means to dramatically scale-up insect surveillance in terms of both the quantity of traps that can be processed concurrently, and number of pest species that can be targeted. In this review of the metabarcoding literature, we explore how DNA metabarcoding could be tailored to the detection of invasive insects in a surveillance context and highlight the unique technical and regulatory challenges that must be considered when implementing high-throughput sequencing technologies into sensitive diagnostic applications.

Keywords:

Biosecurity, Alien species, Biosurveillance, Early detection, Bioinformatics, Reference database, Quality assurance, Controls, Validation, Non-destructive

BACKGROUND

Increasing globalisation of trade and tourism along with changing climates are expected to further increase the rate of biological invasions over coming decades [1]. Insects form a dominant component of this global spread of invasive species [2], posing a major threat to agroecosystems and the environment through disruption of ecological networks, plant herbivory and the transmission of pathogens and disease [3]. Once established in a new environment, ongoing containment and control of invasive insect pests imposes significant costs to industry and government [4], and consequently major efforts are made in forecasting incursion risk [5–7] in order to implement quarantine of entry pathways [8–10]. Despite these measures, the exponential increase in global movement of food, vectors and humans complicates traceability and makes quarantine inspection of more than a fraction arriving cargo an impossible task [11]. Therefore, proactive post-border surveillance within agricultural and natural landscapes is becoming an increasingly important component of effective biosecurity programmes, aiming to detect invasive species early before populations escalate or spread and eradication becomes unfeasible [12–14].

Insect invasions can initiate and disperse across vast and highly heterogenous landscapes [15], and therefore surveillance programmes often involve extensive trapping conducted across a large spatial scale [15,16]. As it is generally unclear whether a new introduction of has occurred, or what species it may be, surveillance programmes can extend over many years and target diverse taxonomic groups. In many cases these surveillance traps will capture non-target endemic species in vast excess of the target pests and the sheer number of specimens that need to be sorted through and identified by highly-trained entomologists forms a major diagnostic bottleneck. While insect diagnostics still largely relies on traditional morphological examination [17], in recent years this has been supplemented by a range of molecular techniques that allow standardised identification of a wide range of taxa without specialist taxonomic expertise (Table

1). DNA barcoding in particular has become a central component of the modern diagnostic toolbox, due to its near universal applicability and standardised protocols that allow transparent and objective comparison of specimen identifications between laboratories and regulatory agencies [17,18]. The universal nature of DNA barcodes is particularly advantageous for early detection surveillance, as when considering impacts beyond just agriculture [19] and the time lag that can occur between introduction of a new species and perceptible damage to the environment [20], there are far more invasive species of threat than can be identified by risk assessment and incorporated into target lists. Despite these promises, the time-consuming process of conducting single PCR and sequencing reactions on individual specimens has restricted the use of DNA barcoding to confirming the identity of specimens already deemed suspect by prior morphological sorting, or for identification of taxa or life stages where a taxonomic key may not be available or key diagnostic structures are degraded or missing [17,21]. Due to the inadequate scalability of current molecular and morphological diagnost methods to large trap catches, current surveillance programmes do not generally identify all specimens to species level [22]. Instead, target lists are confined to relatively few priority pest species identified by previous risk assessment [5] or statistical methods are used to select only a subset of specimens for species level identification [23]. These restrictions can result in unanticipated or cryptic invasive species that are not being actively monitored for, to go undetected [24].

In order to overcome the limitations of current diagnostic methods for processing large numbers of specimens, recent studies have looked to high-throughput sequencing (HTS) technologies to allow DNA barcode based identification to be conducted in a massively-parallel manner (Fig 1A). This process, termed "metabarcoding" [25], generates a large number of individual barcode sequences in a single reaction, enabling the simultaneous identification of individuals in large mixed communities [26,27], such as a trap sample containing many different insect species. By identifying both endemic and potential exotic insects in a bulk DNA analysis approach,

metabarcoding removes the time-consuming specimen sorting required by previous molecular and morphological diagnostic methods, and allows detection of not just key pests but also other unanticipated species that may not be being actively searched for [28–30]. A further advantage arises from the ability of HTS to count occurrences of specific sequences in a mixed sample [31], potentially allowing simultaneous pest identification and population size estimation. Finally, the rapidly increasing output of HTS technologies enables multiplexing of hundreds of trap samples in a single sequencing run, providing an avenue to dramatically scale up insect surveillance to the level required for effective and proactive management response. In this review, we explore the application of metabarcoding to high-throughput species level identification of insects, providing an overview of common metabarcoding workflows (Fig 2) and considerations required at each step to ensure reliable detection and quantification of taxa within complex mixed communities. We further discuss the unique technical and regulatory challenges of integrating broad-spectrum HTS assays into a diagnostic framework and offer a perspective on the future adoption of high-throughput insect surveillance within international biosecurity frameworks.

Table 1: Methods employed for insect identification, with suitability assessed according to accuracy, expertise, general applicability, time and throughput criteria

Identification method	Taxonomic expertise	Identify specific taxa	Identify broad range of taxa	Throughput level	Time per identification
**Morphological**					
Microscopic examination	High	High*	High*	Low	Moderate
**Molecular**					
PCR-RFLP	Low	Moderate	Low	Moderate	Moderate
DNA barcoding	Low	High	High	Low	Moderate
qPCR/ddPCR	Low	High	Low	High	Low
LAMP	Low	High	Low	Low	Low
Metabarcoding	Low	High	High	Very High	Low

* This morphological identification score assumes a high level of taxonomic knowledge and a low human error rate.

Review

Selecting a taxonomic marker

Appropriate selection of a taxonomic marker or barcode loci is a critical first step in design of a metabarcoding assay, as all downstream species recovery and identification will rely on how conserved this marker is across taxa, and the discriminatory power of the nucleotide variation contained within it. The markers most commonly employed in metabarcoding studies are those already widely adopted for conventional DNA barcoding due to the depth of existing reference data available, and therefore the mitochondrial cytochrome oxidase I (COI) locus has been the most widely used marker for metabarcoding of insects to date. Whereas conventional DNA barcoding commonly amplifies a 710bp region of COI [32], many HTS platforms impose strict limitations in molecule length that can be sequenced (Table 2) and therefore smaller stretches of the conventional barcode loci or 'mini-barcodes' are used [33]. Research into degraded DNA samples has shown that a DNA barcode as small as 135bp can reliably distinguish most animal species, however the reduced discriminatory power may necessitate confining analysis to a particular taxonomic group for each primer set [34]. These restrictions in barcode resolution have prompted the design of bespoke primer sets for different taxonomic groups, as well as the use of multiple markers for diverse samples [31,35,36]. This situation has led to a proliferation of metabarcoding primer sets throughout the literature, many of which have not been benchmarked for effectiveness.

The mitochondrial COI locus was initially adopted as the primary DNA barcode for animals due to its high mutation rate and multi-copy nature that provides strong discriminatory power and ease of amplification [37,38]. However, since its application to metabarcoding a number of

limitations have become particularly apparent. As COI is a protein coding gene, the third position of codons can be variable, leaving no strictly conserved nucleotide sites for design of universal PCR primers [39]. This mis-match inevitably leads to primers having variable affinity for different template molecules, biasing the amplification towards well-matched taxa and failing to amplify others [40]. Unlike conventional DNA barcoding where a failed amplification will result in a noticeably absent PCR product, in a bulk sample failed amplification of a particular taxon will be masked by the recovery of sequences from other taxa and therefore will go unnoticed [39]. A further issue inherent to mitochondrial loci is the proliferation of nuclear mitochondrial pseudogenes (numts) in many insect orders [41–44], the result of historical recombination between the mitochondrial and nuclear genomes [45]. Co-amplification or preferential amplification of these pseudogenes instead of the true mitochondrial gene can complicate species identification [44] and result in overestimation of taxonomic diversity in the sample [46].

Due to the aforementioned issues, as well as the inability for COI to differentiate certain pest groups [47], the search for additional barcode markers to replace or supplement COI in metabarcoding has seen significant recent attention. Proposed alternatives include the mitochondrial small subunit (12S) [48,49] and large subunit (16S) ribosomal RNA (rRNA) genes, nuclear small subunit rRNA (18S) [50] and internal transcribed spacer (ITS) regions [51], as well as other group specific nuclear genes where necessary [52,53]. rRNA genes are particularly appealing due to their high copy number and stem-loop structure that consists of highly conserved core sequences for primer binding, interspaced with hypervariable regions providing taxonomic resolution [54,55]. However, these hypervariable regions can differ in length between taxa, introducing further bias [56] and complicating analysis pipelines [57]. Furthermore, the application of these markers to metabarcoding based surveillance will suffer from a lack of reference sequence data, as many insect taxa, including those of economic importance currently

only have COI data publicly available (Fig 2B, 2C). As species level resolution is a requirement of many diagnostic standards [17,58,59], for the groups where COI contains sufficient variation, the extensive reference information obtainable for this loci will maximise the utility of metabarcoding within a broad-spectrum surveillance programme [60].

Box 1 – Reference Sequence Databases

As with conventional DNA barcoding, accurate taxonomic assignment in metabarcoding studies relies on a well-curated reference database of DNA marker sequences tied to vouchered morphological specimens to compare query sequences against [61]. The primary public nucleotide databases of relevance to insect metabarcoding are the Barcode of Life Data System (BOLD) [62] and the NCBI GenBank database [63]. While GenBank hosts greater overall sequence data, BOLD represents a curated DNA barcoding centric database that aims to maintain consistent links between sequences, validated morphological specimens, and associated specimen collection metadata [64]. Concerted efforts to generate mitochondrial COI barcodes for major insect orders have led to broad coverage of insects of quarantine concern in both major public databases [65], however many geographic regions are still under sampled (Fig 2A) and reference sequences for alternative loci are mostly unavailable (Fig 2B, 2C). While continued public submission will increase the representation of missing taxa and loci over time, ensuring the quality of submitted sequences from correctly identified specimens is crucial [17]. There are numerous examples of barcode sequences annotated with the incorrect species in public databases [61,66–69] as well as multiple morpho-species sharing the same DNA barcode, which may reflect misidentifications or the existence of species complexes [65].

Some studies have responded to the these issues by exclusively using in-house reference databases for taxonomic assignment [70–72], however as many insect surveillance programmes

aim to detect species that are not locally present, the reliance on public data may be unavoidable. Regardless of source, all sequences will need to be compiled together and formatted appropriately for use with automatic taxonomic classification software [73–76], and this presents an ideal stage where further automated or semiautomated curation methods can be used in order to identify and remove any taxonomically mislabelled sequences [77,78]. These issues further highlight the importance of engaging taxonomic experts to ensure *a priori* identification of a specimen before submitting a reference barcode to a public database, ideally in conjunction with high quality images and records of diagnostic features used for identification [70]. Furthermore, the use of non-destructive DNA extraction methods would allow the retention of voucher specimens to ensure traceability between the molecular and morphological features, especially in the case of taxonomic reassignments [79].

Marker enrichment

Similar to conventional DNA barcoding, most metabarcoding studies use a set of universal oligonucleotide primers to exponentially amplify a target barcode marker until it reaches a concentration appropriate for sequencing. This PCR based 'Amplicon sequencing' methodology has proven reliable and sensitive for detection of low abundance taxa in bulk samples [80], however differential PCR amplification efficiencies between taxa generally results in a biased depiction of relative abundances of community members [81]. This bias is thought to mainly arise from primer-template mismatches, particularly at the 3' end of the primer [40,82], and therefore appropriate selection of barcode marker and comprehensive in-silico validation of primer sets is important to ensure accurate recovery of all desired taxa with minimal bias [54,83–85]. Where mismatches are predicted to occur, including degenerate bases in the primers can overcome taxonomic bias inherent to a specific primer sequence [86], however high levels of degeneracy can also lead to undesired off-target amplification [49,67]. In addition to the effects

of PCR primers, a range of template specific factors including; copy number of the loci [87], nucleotide composition [88], secondary structure [89], variable amplicon lengths [56], specimen biomass [90], and complexity of the species mixture [82] can further contribute bias. While the cumulative bias from all these factors may suggest that amplicon sequencing can only be used for presence-absence data, importantly, sequencing reads are still correlated with DNA input in a predictable way, and biases should only affect the slope of that correlation [87]. Therefore, the calculation of taxon-specific correction factors shows promise for improving abundance estimates [87,91], particularly for simpler communities such as those trapped using targeted attractant lures [12]. Nevertheless, if accurate quantification is essential for the surveillance programme, removing the PCR amplification process altogether shows the most potential for improving taxon abundance estimates from metabarcoding data.

PCR-free approaches

The major alternative to amplicon sequencing based metabarcoding involves simply fragmenting the genomic DNA extract and directly sequencing it without any prior bias-inducing enrichment step. This methodology, termed 'shotgun metagenomics', generates sequence reads comprising a random subsample of the mixed community DNA and relies on the higher representation of multi-copy mitochondria and nuclear rRNA in this subsample to identify community members [92,93]. In addition, these high copy regions can be assembled into long contigs and even full length mitochondrial genomes for further phylogenetic inference and systematics applications [94,95]. While shotgun metagenomics can improve correlations between sequencing reads and input compared to amplicon sequencing [96,97], biases are likely to still remain due to copy number variation of mitochondria between organisms [98] and tissues [99]. Furthermore, restricting taxonomic analysis to just mitochondrial and nuclear rRNA regions still leaves the vast majority of reads corresponding to DNA that is not taxonomically informative or easily

assembled from a bulk sample to be discarded [92] and costly deep sequencing will be required to reliably detect rare specimens in the community [95,100]. Therefore, for routine surveillance a cost-effective method for enriching taxonomically informative loci such as differential centrifugation [101], rolling circle amplification of mitochondria [102], or probe capture [103] should be used prior to sequencing.

Of the aforementioned enrichment strategies, hybridisation probes currently show the greatest promise as a cost effective PCR alternative, having recently been used to enrich for barcode markers [104–106] and whole mitochondria [107] for community identification. This strategy employs thousands of synthetic oligonucleotide immobilised on a microarray chip [107] or suspended in solution [104–106] to bait target genomic regions out of a complex DNA mixture. Hybridisation relies on strict complementarity of probes with the target sequence [103], and while capture may be possible over moderate phylogenetic distance [106] probes should ideally be designed with *a priori* knowledge of every target sequence [108]. Although this may be a limiting factor for recovery of overall diversity, it offers a more scalable approach to taxon recovery than degenerate PCR primers, and designing a probe set that exclusively captures sequences with existing reference information could ensure only taxonomically useful data is generated from the metabarcoding assay. The flexibility to include essentially infinite numbers of probes provides further advantages for building large 'multifaceted' metabarcoding assays that capture diverse loci for purposes beyond taxonomic inference [109], overcoming limitations of multiplexed PCRs where each additional primer set can increase the risk of cross-reactivity and individual primer sensitivities can change depending on community composition [110].

Library Preparation & Multiplexing

Regardless of whether an enrichment or metagenomics approach was used, platform specific sequencing adapters need to be attached to the molecules (via ligation [111], one-step [112] or two-step PCR [80,85]) to form 'libraries' which can then bind to the flow cell for sequencing (Fig

4A). As current HTS platforms output sequences far in excess of what is required to identify the taxa in a single community, metabarcoding studies commonly multiplex many samples together on a single flow and use oligonucleotide index sequences incorporated into the sequencing adapters to link sequencing reads back to origin sample. While a range of indexing strategies exist for HTS [113], for sensitive applications such as metabarcoding diagnostics it is critical to choose an approach that can adequately cope with the occasional switching of these indices between molecules. Index-switching has seen particular recent attention due to the high levels on recent Illumina platforms [114], however similar phenomena can plague multiplexed sequencing across all major platforms [115–119]. Suggested causes include contamination from residual adapter/primer oligonucleotides [120], chimera formation during adapter PCR [121], mixed clusters on the flow cell [117], or physical contamination during library preparation or oligo synthesis by the vendor [119,122]. Regardless of mechanism, when not properly controlled for, index-switching can cause taxa from one sample to 'bleed' into others, and while this will only produce false positives for a taxon of concern when a true positive is present in at least one of the samples, the spreading of positive signal across samples can imply the taxa of interest has a larger geographic distribution than reality. Recent studies have demonstrated the most effective method for controlling for index-switching is through the use of unique dual indices (Fig 4C) rather than the commonly used combinatorial indexing (Fig 4B). When unique dual indices are used, switching events at either end of the molecule will generate an index combination that was not originally applied and during de-multiplexing the reads with mismatched indices to the sample sheet will be filtered into an unassigned reads file and excluded from analysis [119,121,123]. Furthermore, sets of indices should be alternated for each sequencing run as run-to-run carryover of molecules on a HTS machine can be a further cause of false positives in high sensitivity sequencing applications [124].

Box 2- High-Throughput Sequencing Platforms

The rapid growth of the HTS field over the past decade has produced a variety of platforms and chemistries for discerning the nucleotide sequence of a DNA molecule [125]. While Roche 454 pyrosequencing technology was used for initial metabarcoding studies, this platform was discontinued in 2016 and the Illumina 'MiSeq' has since dominated the metabarcoding literature due to its high-quality reads, relatively low cost per gigabase of data, and ability to target amplicons up to ~550bp in length (Fig 1B). Despite the current popularity of the MiSeq for research studies, the cost per sample may be impractical for the number of specimens produced by large-scale surveillance programmes, especially if metagenomic sequencing with no prior enrichment is used. Instead, the Illumina 'NextSeq', 'HiSeq' and 'NovaSeq' provide progressive increases in throughput and therefore cost reductions (Table 2). However increased sequencing throughput must be balanced with diagnostic turnaround times, and effective use of the production scale HiSeq and NovaSeq flow cells will involve multiplexing of thousands of samples, requiring significant logistical effort in sample collection and handling. Therefore, unless flow-cells can be shared between multiple projects such as in a sequencing core facility, the Illumina NextSeq may be more appropriate for diagnostic labs seeking to invest in a HTS platform for high-throughput surveillance.

Despite the cost-effectiveness of the aforementioned platforms, the increase in per base output has come at the expense of further restricted read lengths (Table 2), which consequently decrease the taxonomic resolution achievable with a metabarcoding assay. Therefore, long read sequencing platforms such as the Pacific Biosciences (PacBio) 'Sequel' and Oxford Nanopore Technologies (ONT) 'MinION' and 'PromethION' are becoming increasingly attractive alternatives for metabarcoding studies. The single molecule sequencing technology used by these platforms allows significantly longer molecules to be sequenced [126] and therefore recovery of greater taxonomic diversity with sub-species resolution [56,118,127,128]. While their higher per-

base error rate currently causes issues differentiating closely related species [26], methods of repeatedly sequencing a target molecule and calling consensus may overcome this. The PacBio Sequel offers natively implemented circular consensus sequencing which produces consensus reads with similar accuracy to traditional Sanger sequencing [129], and third party protocols mimicking this approach have now been published for the ONT platforms [130,131]. If robust consensus sequencing can be achieved for nanopore sequencing, the significantly smaller start-up cost and portability of this technology may in future permit metabarcoding based diagnostics to be conducted in remote field sites [56], as well as enable lesser resourced diagnostic laboratories to access these technologies [10].

Table 2: Comparison of sequence throughputs, error rate and associated costs between high-throughput sequencing platforms.

**Short read platforms**					**Long read platforms**		
	Illumina MiSeq	Illumina NextSeq	Illumina HiSeq 3000/4000	Illumina NovaSeq	PacBio Sequel	ONT MinION	ONT PromethION
**Maximum throughput (Gigabases)**	15Gb	120Gb	750Gb /1500Gb (8/16 lanes)	6000Gb (8 lanes)	20Gb (2 lanes)	20Gb	150Gb per flow cell (up to 48)
**Maximum Read length**	2x300bp	2x150bp	2x150bp	2x150bp	~100kb	~2Mb	~2Mb
**Error rate**	Low	Low	Low	Low	Low (consensus error)	High	High
**Instrument cost**	Low	Medium	High	High	High	Extremely Low	Low
**Cost per flow cell (AUD) ***	~$2500	~$6000	~$24000	~$45,000	~$2000	~$1000	~$2500
**Setup time (labour)**	Medium	Medium	Medium	Medium	High	Low	Low
**Run time**	56hrs	30hrs	84hrs	40hrs	10hrs	1-72hrs	1-64hrs

**Sequencing cost per sample†**	~$42	~$13	~$4	~$2	~$25	~$25	~$5

*Costs are presented in Australian Dollars (AUD) and consider chemistry cost, depreciation, servicing, and computational cost over the lifespan of the instrument, however total costs and read lengths will further depend on marker capture and library preparation methods used. †Assuming pooled sequencing of many traps with 250Mb sequencing effort per sample.

Bioinformatics

Computational processing of sequence reads represents a series of steps of equal importance to laboratory protocols for ensuring accurate and sensitive detection of invasive species [132,133], however many of the skills and techniques involved in this process have not historically been required within diagnostic laboratories. While there exists a number of end-to-end computational pipelines for analysing metabarcoding data, many of these have been designed for measuring diversity rather than detection of low abundance taxa [57,134–136]. Each step in the bioinformatic analysis can present trade-offs between sensitivity to rare taxa, amount of erroneous sequences retained, and overall computing time [31,132] and use of metabarcoding in an invasive surveillance context presents some unique challenges that may be best addressed through the creation of a custom analysis pipeline [133].

De-multiplexing and sequence quality trimming

A metabarcoding assay typically involves multiplexing many samples into a single pooled sequencing library in order to make optimal use of the high capacity flow cells of current sequencing platforms. Therefore, the first step following sequencing (typically automated by the HTS platform's software) is to assign sequences back to their origin sample using unique oligonucleotide sample indices incorporated into the sequencing adapters (Fig 4). Following de-multiplexing, sequencing adapters and any other non-biological information such as PCR primer sequences are trimmed from reads, and forward and reverse reads are assembled into a

consensus sequence using their overlapping bases. Typically only a short stretch of overlapping bases is required for pairing reads together [137], however increasing read overlap by sequencing shorter fragments can improve consensus accuracy, particularly at the ends of reads where quality tends to drop [27,138,139]. While steady improvements in sequencing chemistries has led to major HTS platforms providing per base accuracies above 99.9% [125], when put in context of the billions of bases sequenced on modern flow cells, tens of thousands of sequences will contain errors [137]. Raw reads are generated in conjunction with a predicted error profile, and an initial quality trimming stage uses this profile to truncate or remove sequences that contain excessive ambiguous or low confidence base calls [137,140]. Quality trimming using error profiles is a coarse filtering process and represents an important step where parameters should be carefully considered. While strict quality trimming will more effectively remove sequencing artefacts and erroneous reads that can impact downstream diversity and abundance estimates, overly conservative parameters can result in removal of too many reads and therefore loss of sensitivity to rare occurrences [133].

OTU clustering & Denoising

In addition to sequencing errors, the PCR amplification process used in the majority of metabarcoding studies can further introduce single base substitutions [118] and length variation [141] that will not necessarily be associated with low quality scores [138]. These 'noisy' sequences will appear identical to much more abundant sequences apart from one or more single base errors and can be difficult to distinguish from real biological variation. To account for this noise, sequence reads are typically clustered into distinct subgroups called 'operational taxonomic units' (OTUs), each with a single representative sequence and an associated abundance [142]. The goal of OTU clustering is twofold, firstly it simplifies computation by dereplicating redundant copies of identical sequences, and secondly it will absorb near-identical 'noisy' sequences into the true biological sequence. Similarity clustering using a threshold of 97% has

customarily been used for this purpose, however while this threshold represents a compromise between interspecific and intraspecific variation [38] it is largely arbitrary and can differ greatly across insect taxa [143]. Therefore when the same threshold is used on diverse communities multiple species can be placed within the same OTU, resulting in false negatives [144]. Furthermore, absorbing all closely related sequences into a single OTU will lose all information on intraspecific diversity and restrict the ability to trace geographic origin of invasive populations [30,58]. Finally, as the OTU's generated are emergent properties of the particular dataset and parameters selected, these do lend themselves to ongoing comparison with the constantly evolving data produced by a longitudinal surveillance programme [144–146]. In order to overcome these limitations, recently developed 'de-noising' algorithms instead using statistical models to infer true positive sequences from sequencing noise and correct for single nucleotide differences [147–149]. This single nucleotide resolution enables binning sequences into 'Exact sequence variants' (similar to 100% clustered OTU's) that retain the precise information necessary for diagnostics of closely related taxa [144], and investigation of intra-specific variation [150].

OTU quality control

While the above measures control for most low abundance errors, they are not designed to deal with high abundance artefacts such as PCR generated chimeras and non-specific amplification products. Chimeric sequences are the result of incompletely extended PCR products acting as primers for a different closely related sequence [151], and therefore appear as concatenated products of two parent sequences. Assuming parent sequences will be more abundant having undergone more rounds of amplification, chimeras can be algorithmically removed through comparison with other sequences in the sample [147,152], or with a chimera-free reference database [153]. On the other hand, removing products of non-specific amplification such as intra-genomic variants and pseudogenes presents more of a challenge, and will generally involve

manual curation [111,154]. When targeting protein coding mitochondrial genes such as COI, the presence of stop codons and frameshifts that disrupt the open reading frame (ORF) are common indicators of pseudogenes [60], and for rRNA markers secondary structure prediction could be used to ensure sequences don't contain significant variation in highly conserved regions [155]. As it is inefficient to include a manual curation process as part of a high-throughput bioinformatics pipeline, it would be beneficial for future denoising algorithms to incorporate patterns of sequence evolution for precise and automated quality filtering of pseudogenic sequences [60,154].

Taxonomic assignment

In order to process the large diversity of sequences that a metabarcoding assay typically produces, the assignment of Linnaean taxonomy (species, genus etc.) is conducted in an automated manner. The most widely used approach for this has been best-hit classification using tools such as BLAST [156], which assumes that the taxonomy of the query sequence will be identical to the taxonomy of the most similar sequence or 'best-hit' in a reference database. While best-hit classifiers are simple to implement and can perform effectively when the reference database contains sequence information from conspecifics, when this data is absent or when the particular loci cannot distinguish between multiple organisms they are prone to over-classifying the sequence to incorrect species level taxonomy [78]. In the worst case, this over-classification error could lead to false positives by classifying a previously un-sequenced but probably innocuous organism as a known pest, due to the pest being the closest taxa with an existing reference sequence [143]. While best-hit classification can be coupled with a lowest common ancestor algorithm to instead return a higher taxonomic rank when no hits exceed a minimum similarity threshold [157,158], this faces a similar problem to similarity clustering for OTU's, in that thresholds are not consistent across insect taxa [143].

The use of phylogenetic theory instead of sequence similarity when assigning taxonomy can partly circumvent issues of over-classification, as when evolutionary distance is taken into account it may become clear that the best-hit in the reference database does not necessarily imply phylogenetic proximity [159]. However, traditional methods of phylogeny construction are computationally intractable for large metabarcoding studies and further rely on the sequences containing enough phylogenetic signal to reproduce an acceptable tree, which may not be the case for the short mini-barcodes typically used for metabarcoding [160]. Instead, phylogenetic placement algorithms that sequentially insert query sequences into a pre-assembled reference tree enable a computationally efficient approximation of traditional maximum likelihood analysis [161–164]. Identifying insects using the same phylogenetic framework typically used to delimit species is particularly appealing, however the application of phylogenetic placement relies on the existence of a suitable reference tree assembled with full length sequences. While this may be simple for small taxonomic groups, the creation of a reference tree for the entire Insecta would involve significant computational and curation efforts [165]. While this currently limits the application of phylogenetic placement to metabarcoding of diverse insect samples, as long read sequencing becomes more common this approach may provide the best method to take advantage of the longer barcodes for which reference data is currently scarce [118].

In order to overcome the limitations of the above methods, recent software tools have looked to the field of machine learning for solutions to the taxonomic classification problem [78,166–168]. The use of probabilistic machine learning models provide a promising means for dealing with the often-fuzzy definition of what constitutes a taxonomic group [169], and the ability to return confidence levels makes the classification process more robust to pervasive issues of missing and mis-annotated data in reference databases [170]. The most popular of these are naive Bayes classifiers, which split sequences into short kmers and use repeated random sampling of these against the reference database to estimate the confidence of the query sequences inclusion into

each taxonomic rank [166,171]. In an ideal case, only a single possible taxonomic outcome will obtain a high level of probability, whereas alternate outcomes will obtain probabilities close to zero. In cases where there may be ambiguity due to imperfect reference data and multiple taxonomic outcomes obtain similar probabilities, the sequence may still be robustly assigned to a higher taxonomic rank (e.g. family) [76], providing important information about sample composition and possible presence of novel taxa [172].

Quality Assurance

Controls and replication

The ability to simultaneously identify many loci from thousands of specimens in a single diagnostic assay underlies the power of the metabarcoding approach to surveillance, however the resulting increase in sequence diversity and analytical complexity introduces further risk of cross contamination and technical error. The majority of contamination in metabarcoding assays is expected to arise from other samples processed in the same laboratory environment, and therefore workspaces should be divided into separate pre-PCR, post-PCR and library preparation areas with all surfaces and equipment regularly decontaminated [26,173]. However, the sensitivity of HTS approaches means contaminant sequences will arise even the most cleanly laboratory space. Therefore, no-template controls should be incorporated throughout the entire laboratory workflow and sequenced alongside the real libraries to provide a cumulative measure of uncertainty [121,173,174]. Index-switching is perhaps the most worrisome cause of contaminants in HTS, and while use of unique-dual indices (Fig 1C) can reduce this phenomenon to a level acceptable for most studies, trace levels of index-switching can still persist and cause issues for sensitive diagnostic applications [119]. Index-switching artefacts will be detectable in negative controls, however it can be difficult to discern this phenomenon from sequences arising through physical contamination. Instead, including a positive control library made up of synthetic internal standard DNA [57,175,176] or 'alien' taxa guaranteed to be absent from the sample [68] allows

empirical measurement of the index-switch rate and can inform application of an appropriate filtering threshold [57]. While a single synthetic sequence will effectively serve as a positive control, use of a synthetic community would further highlight any batch effects that may have been introduced during laboratory, sequencing, and bioinformatic stages of the metabarcoding workflow if the composition or abundance of this community differs across sequencing runs [174].

Both DNA extraction and PCR amplification contain a fundamentally random component, and technical replicates are commonly used in metabarcoding studies to overcome stochasticity at these stages and therefore increase the likelihood of capturing loci from rare taxa in the community [31]. These replicates can then be pooled together prior to library preparation [177] or maintained separately through the rest of the workflow to provide a further avenue to identify laboratory cross-contamination in the case that replicates show significant dissimilarities in taxonomic composition. While the importance of technical replication for increasing detection probability is generally agreed upon [31,178,179], replicates reduce throughput without providing further independent data points [26]. Instead, with metabarcoding removing one of the major roadblocks to large-scale surveillance, more biological replicates from more frequent and intensive trapping could be used. Considering biological replication is particularly important as regardless of the effectiveness of the metabarcoding diagnostic assay if an insect is not caught in a trap it does not necessarily mean absence in the area. With insect community metabarcoding relying on the trapping of physical specimens, there is much higher likelihood of missing taxa at the field collection stage than in the actual diagnostic assay. Therefore, while out of the scope of this review, appropriate trap design [180] and surveillance grid planning [181] must also be adhered to for effective metabarcoding based surveillance.

Validating metabarcoding assays

Due to the relevance of many invasive insects to international trade and human health, laboratories conducting insect diagnostics generally exist within strict regional and international regulatory environments. As part of laboratory accreditations, newly developed assays are required undergo a validation process in order to provide objective evidence to all end users that an assay is fit for purpose [182,183]. Traditionally, validation first involves defining the scope of the assay and then establishing performance parameters such as sensitivity, specificity, reproducibility and repeatability for every individual target designated in this scope [22,53,183]. However, the universal nature of metabarcoding assays and diversity of potential surveillance catch makes this impractical [184]. Instead, a methods-based validation process should be used to establish performance parameters on representative samples and identify critical steps in the workflow where variation can be introduced. These critical steps can then be monitored run-to-run using control samples and appropriate QC checkpoints (Table 3), in order to ensure that no sample or sequence data continues without meeting minimum quality requirements [53,185–187]. In the case of insect metabarcoding, mock communities made up of the taxonomic groups of interest are generally used for validation [177,188], spiking these communities decreasing concentrations of target species can be used to establish assay sensitivity and limits of detection [177]. As DNA extraction efficiency [188] and taxonomic bias [82] can be affected by overall community complexity, mock communities should as closely as possible represent the diversity expected to be recovered in different trapping scenarios. Furthermore, the amount of sequencing effort assigned to an individual sample during multiplexed sequencing can vary across runs [173,189], and the effect of sequencing depth on detection should also be established using rarefaction curves [84]. Finally, parameters such as precision and reproducibility of a metabarcoding assay can be established similar to other molecular diagnostics, through replication of sequencing runs, and inter-laboratory comparisons [53].

Reporting & confirming detections

Even when primers are designed around a specific taxonomic group, metabarcoding can amplify and detect many more taxa outside the scope of original validation. How these incidental detections are reported and eventually acted upon will present a major challenge to diagnostic labs and end users, due to the increased number of previously undocumented taxa being discovered for which knowledge of distribution or ecological significance may be missing [187]. Many of these incidental detections will be taxa that simply have not previously been searched for, hence a greater emphasis needs to be placed on conducting baseline surveys to establish comprehensive species checklists of endemic diversity at the beginning of a surveillance programme in order to avoid creating sudden market access and trade issues [187]. Furthermore, a decision framework should be developed for evaluating incidental detections that sets out steps for further characterization and risk assessment for the detected organisms in order to establish if eradication or other management actions are appropriate or achievable [190]. However, before any results are reported to the end user putative detections should be confirmed using an orthogonal diagnostic method. While the use of species-specific molecular assays such as qPCR/ddPCR on the original DNA extract could confirm detections, these assays require prior development and will therefore not be available for all incidental taxa detected in a metabarcoding assay. Instead, the use of non-destructive DNA extraction methods that use a combination of enzymes, buffers and heat without mechanical homogenisation would take full advantage of having access to physical specimens rather than environmental nucleic acids and enable diagnosticians to revisit original samples following metabarcoding to confirm species detections [191,192]. Development of a non-destructive metabarcoding assay has great potential for bridging the gap between new HTS methods and traditional entomological techniques, and may bootstrap the acceptance of metabarcoding into international regulatory frameworks.

Perspectives & conclusions

The ability to accurately, rapidly and cost-effectively determine the species composition of bulk insect traps using metabarcoding has the potential to revolutionise broad-spectrum surveillance for invasive insect pests. Similar to any novel technology, as metabarcoding transitions from purely research to management applications it faces the growing pains that come with integration into established regulatory structures. While rigorous standardisation of both laboratory techniques and data analysis has proven essential for the acceptance of conventional DNA barcoding as a validated diagnostic for quarantine organisms [58], the sheer pace of development of HTS technologies and platforms may complicate similar for metabarcoding. Historically, the effective lifespan of many HTS platforms has only amounted to a few years before obsolescence [125], and laboratory protocols and bioinformatic methods are therefore constantly evolving to chase this moving target. In response to this complex state of the art, efforts towards standardisation should avoid the over prescription of restrictive standards, as these will become quickly outdated and risk further widening the gap between research and diagnostics capabilities. Instead, development and distribution of certified reference materials in the form of both standardised mock community DNA mixtures [193] and computational datasets [194] would enable benchmarking of laboratory methods and begin to characterise the sources of technical variation between laboratories [195,196]. This could be further developed into inter-laboratory proficiency testing program where blinded reference samples are periodically distributed for analysis, in order to demonstrate to all stakeholders that an assay is fit for purpose for detecting invasive insect species [185,197]. The results of these processes would allow further development of best-practice technical guidelines and begin to harmonise approaches across the wider metabarcoding community [198].

Biosecurity and pest management decision making is still largely reliant on the application of a species name to a specimen sequence, and issues of mislabelled sequences in public reference

databases (Box 1) highlight the importance of maintaining expertise in taxonomy and classical diagnostics to complement high-throughput approaches. Due to the incomplete nature of reference databases, much of the sequence data currently produced by metabarcoding assays will consist of insufficiently identified sequences [64]. While some of these will no doubt represent sequencing errors, many more will represent real taxa and reflect how much further work is needed to describe and acquire reference data for insect biodiversity. Embracing non-destructive DNA extraction techniques in metabarcoding protocols would enable taxonomists to retrieve specimens from communities that had low identification success and potentially locate previously unbarcoded taxa or novel species, which could then feed back into reference databases. Conventional DNA barcoding and morphological taxonomy currently benefit from a close and reciprocal interaction [199], and we envision a similar relationship for the future of insect metabarcoding. A further strength of HTS diagnostics lies in the ability to systematically reanalyse historical datasets with improved reference databases, bioinformatic tools, and biological knowledge [187] and therefore raw datasets should be archived alongside relevant technical and environmental metadata in a machine readable format [200]. However datasets from ongoing longitudinal surveillance will quickly amount to terabytes of data [201], the storage, management and securing of which will require dedicated infrastructure and personnel. Unlike the current drive for open sharing of data in academic research, concerns of misuse harming the international movement of goods means that historically the release of raw diagnostic data to the public has not been common [187]. However, a pathway for releasing this data to researchers should be developed, as the mass of community level information generated by metabarcoding biosurveillance shows great potential for generating new insights into the process and impacts of biological invasion [202].

In an increasingly globalised world, more effective and scalable utilisation of surveillance activities will be required to manage the spread and establishment of invasive organisms. While

current costs of technological investments can be high, we expect developments in portable real time sequencing will further enhance the availability of these tools to diagnostics labs. It is conceivable that the further miniaturisation of sequencers may synergise with advances in microfluidic and lab-on-a-chip technologies [203] to produce a new generation of metabarcoding based "smart-traps" for remote monitoring [204,205]. Furthermore, while the field of invasion biology has traditionally been concerned with the transport and movement of species, this doctrine overlooks the intra-specific movement of genetic material such as pesticide resistance alleles [206], transposable elements [207], and genetically modified organisms [208]. The ability to capture essentially any loci in a single metabarcoding assay has allowed simultaneous identification of host and associated viruses and pathogens [80,209], and sexing and genotyping organisms in bulk samples [109], and may allow integration with a more gene focused model of biosecurity in the future. In order to take complete advantage of the opportunities metabarcoding diagnostics offers, we encourage future research to involve closer collaboration between academic scientists, diagnosticians and the end users that rely on effective surveillance data to manage the spread of invasive insect pests.

Methods:

Analysis of the Scopus database was conducted on 2018-12-06 using the rscopus package [210] in R 3.4.4 [211] and all articles containing "Metabarcod*" in abstract, title or keywords as well as a number of additional search terms (Supplementary 1) were retrieved and represented graphically by year of publication using ggplot2 [212]. A list of global insect pests was then retrieved from Ashfaq et al [65] and combined with additional pests of concern for Australia [213]. This list was filtered to retain only unique and complete genus species binomials, retaining 558 species for which all records as well as the entire Insecta were retrieved from BOLD using the bold package [214]. The list of genes successfully retrieved from BOLD used to query GenBank and all records for species on the pest list and the entire Insecta were retrieved from

using the Rentrez R package [215]. Records from all databases were combined and specimen collection information was extracted using the R and the biofiles package [216]. Of the 5589069 records for all loci in the datasets, 4603488 were annotated with latitude and longitude information and these were plotted on a world map using ggmap [217]. The number of overall records and unique species within all datasets were then plotted for the top 10 occurring loci.

DECLARATIONS:

Ethics approval and consent to participate

Not applicable

Consent for publication

Not applicable

Availability of data and materials

A snapshot of the datasets and R markdown documents implementing the analyses contained in this manuscript are available at https://doi.org/10.5281/zenodo.2537379

Competing interests

The authors declare that they have no competing interests.

Funding

This work was supported by the Plant Biosecurity Cooperative Research Centre (PBCRC #2153) and Horticulture Innovation Australia (ST16010). AP was further supported by Agriculture Victoria's Improved Market Access for Horticulture program and an Australian Government Research Training Program Scholarship.

Authors' contributions

AP and MB conceptualized the manuscript. AP drafted the manuscript with contributions from JB, JW, JC, NC, BR and MB. All authors read and approved the final manuscript.

REFERENCES:

1. Chown SL, Hodgins KA, Griffin PC, Oakeshott JG, Byrne M, Hoffmann AA. Biological invasions, climate change and genomics. Evol Appl. 2015;8:23–46.

2. Hulme PE. Trade, transport and trouble: Managing invasive species pathways in an era of globalization. J Appl Ecol. 2009;46:10–8.

3. Paini DR, Sheppard AW, Cook DC, De Barro PJ, Worner SP, Thomas MB. Global threat to agriculture from invasive species. Proc Natl Acad Sci. 2016;113:7575–9.

4. Bradshaw CJA, Leroy B, Bellard C, Roiz D, Albert C, Fournier A, et al. Massive yet grossly underestimated global costs of invasive insects. Nat Commun. 2016;7.

5. Andersen MC, Adams H, Hope B, Powell M. Risk Assessment for Invasive Species. Risk Anal. 2004;24.

6. Simberloff D, Martin JL, Genovesi P, Maris V, Wardle DA, Aronson J, et al. Impacts of biological invasions: What's what and the way forward. Trends Ecol Evol. 2013;28:58–66.

7. Lodge DM, Simonin PW, Burgiel SW, Keller RP, Bossenbroek JM, Jerde CL, et al. Risk Analysis and Bioeconomics of Invasive Species to Inform Policy and Management. Annu Rev Environ Resour. 2016;41:453–88.

8. Martin RR, Constable F, Tzanetakis IE. Quarantine Regulations and the Impact of Modern Detection Methods. Annu Rev Phytopathol. 2016;54:189–205.

9. Schrader G, Unger JG. Plant quarantine as a measure against invasive alien species: The framework of the International Plant Protection Convention and the plant health regulations in the European Union. Biol Invasions. 2003;5:357–64.

10. Early R, Bradley BA, Dukes JS, Lawler JJ, Olden JD, Blumenthal DM, et al. Global threats from invasive alien species in the twenty-first century and national response capacities. Nat Commun. 2016;7:12485.

11. Meyerson LA, Mooney HA. Invasive alien species in an era of globalization. Front Ecol Environ. 2007;5:199–208.

12. Liebhold AM, Berec L, Brockerhoff EG, Epanchin-Niell RS, Hastings A, Herms DA, et al. Eradication of Invading Insect Populations: From Concepts to Applications. Annu Rev Entomol. 2016;61:335–52.

13. Trebitz AS, Hoffman JC, Darling JA, Pilgrim EM, Kelly JR, Brown EA, et al. Early detection monitoring for aquatic non-indigenous species: Optimizing surveillance, incorporating advanced technologies, and identifying research needs. J Environ Manage. Elsevier Ltd; 2017;202:299–310.

14. Anderson C, Low-Choy S, Whittle P, Taylor S, Gambley C, Smith L, et al. Australian plant biosecurity surveillance systems. Crop Prot. 2017;100:8–20.

15. Epanchin-Niell RS, Haight RG, Berec L, Kean JM, Liebhold AM. Optimal surveillance and

eradication of invasive species in heterogeneous landscapes. Ecol Lett. 2012;15:803–12.

16. Barrett S, Whittle P, Mengersen K, Stoklosa R. Biosecurity threats: The design of surveillance systems, based on power and risk. Environ Ecol Stat. 2010;17:503–19.

17. Hodgetts J, Ostojá-Starzewski JC, Prior T, Lawson R, Hall J, Boonham N. DNA barcoding for biosecurity: case studies from the UK plant protection program. Genome. 2016;59:1033–48.

18. Armstrong KF, Ball SL. DNA Barcodes for Biosecurity: Invasive Species Identification. Philos Trans Biol Sci. 2005;360:1813–23.

19. Blackburn TM, Essl F, Evans T, Hulme PE, Jeschke JM, Kühn I, et al. A Unified Classification of Alien Species Based on the Magnitude of their Environmental Impacts. PLoS Biol. 2014;12.

20. Epanchin-Niell RS, Liebhold AM. Benefits of invasion prevention: Effect of time lags, spread rates, and damage persistence. Ecol Econ. Elsevier B.V.; 2015;116:146–53.

21. Armstrong K. DNA barcoding: A new module in New Zealand's plant biosecurity diagnostic toolbox. EPPO Bull. 2010;40:91–100.

22. Blaser S, Diem H, von Felten A, Gueuning M, Andreou M, Boonham N, et al. From laboratory to point of entry: development and implementation of a loop-mediated isothermal amplification (LAMP)-based genetic identification system to prevent introduction of quarantine insect species. Pest Manag Sci. 2018;74:1504–12.

23. Raghu S, Hulsman K, Clarke a R, Drew RAI. A rapid method of estimating cathes of abundant fruit fly species (Diptera: Tephritidae) in modified Steiner traps. Aust J Entomol. 2000;39:15–9.

24. Morais P, Reichard M. Cryptic invasions: A review. Sci Total Environ. 2018;613–614:1438–48.

25. Taberlet P, Coissac E, Pompanon F, Brochmann C, Willerslev E. Towards next-generation biodiversity assessment using DNA metabarcoding. Mol Ecol. 2012;21:2045–50.

26. Tedersoo L, Drenkhan R, Anslan S, Morales-Rodriguez C, Cleary M. High-throughput identification and diagnostics of pathogens and pests: overview and practical recommendations. Mol Ecol Resour. 2018;1–30.

27. Porter TM, Hajibabaei M. Scaling up: A guide to high throughput genomic approaches for biodiversity analysis. Mol Ecol. 2018;

28. Comtet T, Sandionigi A, Viard F, Casiraghi M. DNA (meta)barcoding of biological invasions: a powerful tool to elucidate invasion processes and help managing aliens. Biol Invasions. 2015;17:905–22.

29. Simmons M, Tucker A, Chadderton WL, Jerde CL, Mahon AR, Taylor E. Active and passive environmental DNA surveillance of aquatic invasive species. Can J Fish Aquat Sci. 2016;73:76–83.

30. Darling JA, Blum MJ. DNA-based methods for monitoring invasive species: A review and prospectus. Biol Invasions. 2007;9:751–65.

31. Alberdi A, Aizpurua O, Gilbert MTP, Bohmann K. Scrutinizing key steps for reliable

metabarcoding of environmental samples. Methods Ecol Evol. 2018;9:134–47.

32. Folmer O, Black M, Hoeh W, Lutz R, Vrijenhoek R. DNA primers for amplification of mitochondrial cytochrome c oxidase subunit I from diverse metazoan invertebrates. Mol Mar Biol Biotechnol. 1994;3:294–9.

33. Gan H, Sing K, Lee P, Lim P, Wilson J. DNA metabarcoding of insects and allies : an evaluation of primers and pipelines. 2017;717–27.

34. Hajibabaei M, Smith MA, Janzen DH, Rodriguez JJ, Whitfield JB, Hebert PDN. A minimalist barcode can identify a specimen whose DNA is degraded. Mol Ecol Notes. 2006;6:959–64.

35. Zhang GK, Chain FJJ, Abbott CL, Cristescu ME. Metabarcoding using multiplexed markers increases species detection in complex zooplankton communities. Evol Appl. 2018;11:1901–14.

36. De Barba M, Miquel C, Boyer F, Mercier C, Rioux D, Coissac E, et al. DNA metabarcoding multiplexing and validation of data accuracy for diet assessment: Application to omnivorous diet. Mol Ecol Resour. 2014;14:306–23.

37. Hebert PDN, Ratnasingham S, de Waard JR. Barcoding animal life: cytochrome c oxidase subunit 1 divergences among closely related species. Proc R Soc B Biol Sci. 2003;270:S96–9.

38. Hebert PDN, Cywinska A, Ball SL, DeWaard JR. Biological identifications through DNA barcodes. Proc R Soc B Biol Sci. 2003;270:313–21.

39. Deagle BE, Jarman SN, Coissac E, Pompanon F, Taberlet P. DNA metabarcoding and the cytochrome c oxidase subunit I marker: Not a perfect match. Biol Lett. 2014;10.

40. Piñol J, Mir G, Gomez-Polo P, Agustí N. Universal and blocking primer mismatches limit the use of high-throughput DNA sequencing for the quantitative metabarcoding of arthropods. Mol Ecol Resour. 2015;15:819–30.

41. Song H, Moulton MJ, Whiting MF. Rampant nuclear insertion of mtDNA across diverse lineages with in Orthoptera (Insecta). PLoS One. 2014;9:41–3.

42. Pamilo P, Viljakainen L, Vihavainen A. Exceptionally high density of NUMTs in the honeybee genome. Mol Biol Evol. 2007;24:1340–6.

43. Hlaing T, Tun-lin W, Somboon P, Socheat D, Setha T, Min S, et al. Mitochondrial pseudogenes in the nuclear genome of Aedes aegypti mosquitoes: implications for past and future population genetic studies. 2009;12:1–12.

44. Blacket MJ, Semeraro L, Malipatil MB. Barcoding Queensland Fruit Flies (Bactrocera tryoni): Impediments and improvements. Mol Ecol Resour. 2012;12:428–36.

45. Bensasson D, Zhang DX, Hartl DL, Hewitt GM. Mitochondrial pseudogenes: Evolution's misplaced witnesses. Trends Ecol Evol. 2001;16:314–21.

46. Song H, Buhay JE, Whiting MF, Crandall KA. Many species in one: DNA barcoding overestimates the number of species when nuclear mitochondrial pseudogenes are coamplified. Proc Natl Acad Sci. 2008;105:13486–91.

47. Jiang F, Jin Q, Liang L, Zhang AB, Li ZH. Existence of species complex largely reduced barcoding success for invasive species of Tephritidae: A case study in Bactrocera spp. Mol Ecol Resour. 2014;14:1114–28.

48. Elbrecht V, Taberlet P, Dejean T, Valentini A, Usseglio-Polatera P, Beisel J-N, et al. Testing the potential of a ribosomal 16S marker for DNA metabarcoding of insects. PeerJ. 2016;4:e1966.

49. Marquina D, Andersson AF, Ronquist F. New mitochondrial primers for metabarcoding of insects, designed and evaluated using in silico methods. Mol Ecol Resour. 2018;325688.

50. Hadziavdic K, Lekang K, Lanzen A, Jonassen I, Thompson EM, Troedsson C. Characterization of the 18s rRNA gene for designing universal eukaryote specific primers. PLoS One. 2014;9.

51. Batovska J, Cogan NOI, Lynch SE, Blacket MJ. Using next-generation sequencing for DNA barcoding: Capturing allelic variation in ITS2. G3. 2017;7:19–29.

52. Krosch MN, Schutze MK, Strutt F, Clarke AR, Cameron SL. A transcriptome-based analytical workflow for identifying loci for species diagnosis: A case study with Bactrocera fruit flies (Diptera: Tephritidae). Austral Entomol. 2017;

53. PM 7/129 (1) DNA barcoding as an identification tool for a number of regulated pests. EPPO Bull. 2016;46:501–37.

54. Clarke LJ, Soubrier J, Weyrich LS, Cooper A. Environmental metabarcodes for insects: In silico PCR reveals potential for taxonomic bias. Mol Ecol Resour. 2014;14:1160–70.

55. Gillespie JJ, Johnston JS, Cannonone JJ, Gutell RR. Characteristics of the nuclear (18S, 5.8S, 28S and 5S) and mitochondrial (12S and 16S) rRNA genes of Apis mellifera (Insecta: Hymenoptera): structure, organization, and retrotransposable elements. Insect Mol Biol. 2006;15:657–86.

56. Krehenwinkel H, Pomerantz A, Henderson JB, Kennedy SR, Lim Y, Swamy V, et al. Nanopore sequencing of long ribosomal DNA amplicons enables portable and simple biodiversity assessments with high phylogenetic resolution across broad taxonomic scale. bioRxiv. 2018;

57. Palmer JM, Jusino MA, Banik MT, Lindner DL. Non-biological synthetic spike-in controls and the AMPtk software pipeline improve fungal high throughput amplicon sequencing data. PeerJ. 2017;213470.

58. Floyd R, Lima J, de Waard J, Humble L, Hanner R. Common goals: Policy implications of DNA barcoding as a protocol for identification of arthropod pests. Biol Invasions. 2010;12:2947–54.

59. Clover G, Hammons S, Unger JG. International diagnostic protocols for regulated plant pests. EPPO Bull. 2010;40:24–9.

60. Andújar C, Arribas P, Yu DW, Vogler AP, Emerson BC. Why the COI barcode should be the community DNA metabarcode for the metazoa. Mol Ecol. 2018;27:3968–75.

61. Boykin LM, Armstrong K, Kubatko L, De Barro P. DNA barcoding invasive insects: Database roadblocks. Invertebr Syst. 2012;26:506–14.

62. Ratnasingham S, Hebert PDN. BOLD : The Barcode of Life Data System (www.barcodinglife.org). Mol Ecol Notes. 2007;7:355–64.

63. Benson DA, Cavanaugh M, Clark K, Karsch-Mizrachi I, Ostell J, Pruitt KD, et al. GenBank. Nucleic Acids Res. 2018;46:D41–7.

64. Porter TM, Hajibabaei M. Over 2.5 million sequences in GenBank and Growing. PLoS One. 2018;13:e0200177.

65. Ashfaq M, Hebert PDN, Naaum A. DNA barcodes for bio-surveillance: Regulated and economically important arthropod plant pests. Genome. 2016;59:933–45.

66. Shen YY, Chen X, Murphy RW. Assessing DNA Barcoding as a Tool for Species Identification and Data Quality Control. PLoS One. 2013;8:e57125.

67. Mioduchowska M, Jan M, Gołdyn B, Kur J, Sell J. Instances of erroneous DNA barcoding of metazoan invertebrates: Are universal cox1 gene primers too "universal"? PLoS One. 2018;1–16.

68. Galan M, Pons JB, Tournayre O, Pierre É, Leuchtmann M, Pontier D, et al. Metabarcoding for the parallel identification of several hundred predators and their prey: Application to bat species diet analysis. Mol Ecol Resour. 2018;18:474–89.

69. Bengtsson-Palme J, Boulund F, Edström R, Feizi A, Johnning A, Jonsson VA, et al. Strategies to improve usability and preserve accuracy in biological sequence databases. Proteomics. 2016;16:2454–60.

70. Batovska J, Blacket MJ, Brown K, Lynch SE. Molecular identification of mosquitoes (Diptera: Culicidae) in southeastern Australia. Ecol Evol. 2016;6:3001–11.

71. Carew ME, Nichols SJ, Batovska J, St Clair R, Murphy NP, Blacket MJ, et al. A DNA barcode database of Australia's freshwater macroinvertebrate fauna. Mar Freshw Res. 2017;1788–802.

72. Kocher A, Gantier JC, Gaborit P, Zinger L, Holota H, Valiere S, et al. Vector soup: high-throughput identification of Neotropical phlebotomine sand flies using metabarcoding. Mol Ecol Resour. 2017;17:172–82.

73. Machida RJ, Leray M, Ho SL, Knowlton N. Data Descriptor: Metazoan mitochondrial gene sequence reference datasets for taxonomic assignment of environmental samples. Sci Data. 2017;4:1–7.

74. Richardson R, Bengtsson-Palme J, Gardiner MM, Johnson RM. A reference cytochrome c oxidase subunit I database curated for hierarchical classification of arthropod metabarcoding data. PeerJ. 2018;1–19.

75. Axtner J, Crampton-Platt A, Hoerig LA, Mohamed A, Xu CCY, Yu DW, et al. An efficient and robust laboratory workflow and tetrapod database for larger scale eDNA studies. bioRxiv. 2018;345082.

76. Porter TM, Hajibabaei M. Automated high throughput animal CO1 metabarcode classification. Sci Rep. 2018;8:4226.

77. Kozlov AM, Zhang J, Yilmaz P, Glöckner FO, Stamatakis A. Phylogeny-aware identification and correction of taxonomically mislabeled sequences. Nucleic Acids Res. 2016;44:5022–33.

78. Murali A, Bhargava A, Wright ES. IDTAXA: a novel approach for accurate taxonomic classification of microbiome sequences. Microbiome. 2018;6:140.

79. Castalanelli MA, Severtson DL, Brumley CJ, Szito A, Foottit RG, Grimm M, et al. A rapid non-destructive DNA extraction method for insects and other arthropods. J Asia Pac Entomol. 2010;13:243–8.

80. Batovska J, Lynch SE, Cogan NOI, Brown K, Darbro JM, Kho EA, et al. Effective mosquito and arbovirus surveillance using metabarcoding. Mol Ecol Resour. 2018;18:32–40.

81. Pawluczyk M, Weiss J, Links MG, Egaña Aranguren M, Wilkinson MD, Egea-Cortines M. Quantitative evaluation of bias in PCR amplification and next-generation sequencing derived from metabarcoding samples. Anal Bioanal Chem. 2015;407:1841–8.

82. Piñol J, Senar MA, Symondson WOC. The choice of universal primers and the characteristics of the species mixture determines when DNA metabarcoding can be quantitative. Mol Ecol. 2018;00:1–13.

83. Elbrecht V, Leese F. PrimerMiner: an r package for development and in silico validation of DNA metabarcoding primers. Methods Ecol Evol. 2017;8:622–6.

84. Bylemans J, Gleeson DM, Hardy CM, Furlan E. Toward an ecoregion scale evaluation of eDNA metabarcoding primers: A case study for the freshwater fish biodiversity of the Murray-Darling Basin (Australia). Ecol Evol. 2018;8697–712.

85. Rennstam Rubbmark O, Traugott M, Sint D, Horngacher N. A broadly-applicable COI primer pair and an efficient single tube amplicon library preparation protocol for metabarcoding. Ecol. Evol. 2018.

86. Elbrecht V, Leese F. Validation and Development of COI Metabarcoding Primers for Freshwater Macroinvertebrate Bioassessment. Front Environ Sci. 2017;5:1–11.

87. Krehenwinkel H, Wolf M, Lim JY, Rominger AJ, Simison WB, Gillespie RG. Estimating and mitigating amplification bias in qualitative and quantitative arthropod metabarcoding. Sci Rep. 2017;7:1–12.

88. Nichols R V, Vollmers C, Newsom LA, Wang Y, Heintzman PD, Leighton M, et al. Minimizing polymerase biases in metabarcoding. Mol Ecol Resour. 2018;

89. D'haene B, Vandesompele J, Hellemans J. Accurate and objective copy number profiling using real-time quantitative PCR. Methods. .; 2010;50:262–70.

90. Elbrecht V, Peinert B, Leese F. Sorting things out: Assessing effects of unequal specimen biomass on DNA metabarcoding. Ecol Evol. 2017;7:6918–26.

91. Thomas AC, Deagle BE, Eveson JP, Harsch CH, Trites AW. Quantitative DNA metabarcoding: Improved estimates of species proportional biomass using correction factors derived from control material. Mol Ecol Resour. 2016;16:714–26.

92. Crampton-Platt A, Yu DW, Zhou X, Vogler AP. Mitochondrial metagenomics: letting the genes out of the bottle. Gigascience. GigaScience; 2016;5:15.

93. Gómez-Rodríguez C, Crampton-Platt A, Timmermans MJTN, Baselga A, Vogler AP. Validating the power of mitochondrial metagenomics for community ecology and phylogenetics of complex assemblages. Methods Ecol Evol. 2015;6:883–94.

94. Linard B, Crampton-Platt A, Moriniere J, Timmermans MJTN, Andújar C, Arribas P, et al. The contribution of mitochondrial metagenomics to large-scale data mining and phylogenetic analysis of Coleoptera. Mol Phylogenet Evol. 2018;128:1–11.

95. Papadopoulou A, Taberlet P, Zinger L. Metagenome skimming for phylogenetic community ecology: A new era in biodiversity research. Mol Ecol. 2015;24:3515–7.

96. Tang M, Hardman CJ, Ji Y, Meng G, Liu S, Tan M, et al. High-throughput monitoring of wild bee diversity and abundance via mitogenomics. 2015;1034–43.

97. Wilson JJ, Brandon-Mong GJ, Gan HM, Sing KW. High-throughput terrestrial biodiversity assessments: mitochondrial metabarcoding, metagenomics or metatranscriptomics? Mitochondrial DNA Part A DNA Mapping, Seq Anal. 2018;0:1–8.

98. Palmeira CM, Rolo AP. PCR Based Determination of Mitochondrial DNA Copy Number in Multiple Species. Mitochondrial Regul Methods Protoc. 2014;1–194.

99. Moraes C. What regulates mitochondrial DNA copy number in animal cells? Trends Genet. 2001;17:199–205.

100. Arribas P, Andújar C, Hopkins K, Shepherd M, Vogler AP. Metabarcoding and mitochondrial metagenomics of endogean arthropods to unveil the mesofauna of the soil. Methods Ecol Evol. 2016;7:1071–81.

101. Macher JN, Zizka VMA, Weigand AM, Leese F. A simple centrifugation protocol for metagenomic studies increases mitochondrial DNA yield by two orders of magnitude. Methods Ecol Evol. 2018;9:1070–4.

102. Wolff JN, Shearman DCA, Brooks RC, Ballard JWO. Selective enrichment and sequencing of whole mitochondrial genomes in the presence of nuclear encoded mitochondrial pseudogenes (Numts). PLoS One. 2012;7:1–7.

103. Mamanova L, Coffey AJ, Scott CE, Kozarewa I, Turner EH, Kumar A, et al. Target-enrichment strategies for next-generation sequencing. Nat. Methods. 2010. p. 111–8.

104. Dowle EJ, Pochon X, C. Banks J, Shearer K, Wood SA. Targeted gene enrichment and high-throughput sequencing for environmental biomonitoring: a case study using freshwater macroinvertebrates. Mol Ecol Resour. 2016;16:1240–54.

105. Wilcox TM, Zarn KE, Piggott MP, Young MK, McKelvey KS, Schwartz MK. Capture enrichment of aquatic environmental DNA: A first proof of concept. Mol Ecol Resour. 2018;0–3.

106. Peñalba J V., Smith LL, Tonione MA, Sass C, Hykin SM, Skipwith PL, et al. Sequence capture using PCR-generated probes: A cost-effective method of targeted high-throughput sequencing for nonmodel organisms. Mol Ecol Resour. 2014;14:1000–10.

107. Liu S, Wang X, Xie L, Tan M, Li Z, Su X, et al. Mitochondrial capture enriches mito-DNA 100 fold, enabling PCR-free mitogenomics biodiversity analysis. Mol Ecol Resour. 2016;16:470–9.

108. Jones MR, Good JM. Targeted capture in evolutionary and ecological genomics. Mol Ecol. 2016;25:185–202.

109. Swift JF, Lance RF, Guan X, Britzke ER, Lindsay DL, Edwards CE. Multifaceted DNA metabarcoding: Validation of a noninvasive, next-generation approach to studying bat populations. Evol Appl. 2018;11:1120–38.

110. Sint D, Raso L, Traugott M. Advances in multiplex PCR: Balancing primer efficiencies and improving detection success. Methods Ecol Evol. 2012;3:898–905.

111. Saitoh S, Aoyama H, Fujii S, Sunagawa H, Nagahama H, Akutsu M, et al. A quantitative

protocol for DNA metabarcoding of springtails (Collembola). Genome. 2016;59:705–23.

112. Elbrecht V, Leese F. Can DNA-based ecosystem assessments quantify species abundance? Testing primer bias and biomass-sequence relationships with an innovative metabarcoding protocol. PLoS One. 2015;10:1–16.

113. Gohl DM, Vangay P, Garbe J, MacLean A, Hauge A, Becker A, et al. Systematic improvement of amplicon marker gene methods for increased accuracy in microbiome studies. Nat Biotechnol. 2016;34:942–9.

114. Sinha R, Stanley G, Gulati GS, Ezran C, Travaglini KJ, Wei E, et al. Index Switching Causes “Spreading-Of-Signal” Among Multiplexed Samples In Illumina HiSeq 4000 DNA Sequencing. bioRxiv. 2017;125724.

115. Wick RR, Judd LM, Holt KE. Deepbinner : Demultiplexing barcoded Oxford Nanopore reads with deep convolutional neural networks. PLoS Comput Biol. 2018;14:e1006583.

116. Carlsen T, Aas AB, Lindner D, Vrålstad T, Schumacher T, Kauserud H. Don’t make a mista(g)ke: Is tag switching an overlooked source of error in amplicon pyrosequencing studies? Fungal Ecol. 2012;5:747–9.

117. Kircher M, Sawyer S, Meyer M. Double indexing overcomes inaccuracies in multiplex sequencing on the Illumina platform. Nucleic Acids Res. 2012;40:1–8.

118. Tedersoo L, Tooming-Klunderud A, Anslan S. PacBio metabarcoding of Fungi and other eukaryotes: errors, biases and perspectives. New Phytol. 2017;

119. Costello M, Fleharty M, Abreu J, Farjoun Y, Ferriera S, Holmes L, et al. Characterization and remediation of sample index swaps by non-redundant dual indexing on massively parallel sequencing platforms. BMC Genomics. 2018;19:1–10.

120. Illumina. Effects of Index Misassignment on Multiplexing and Downstream Analysis. 2017.

121. Schnell IB, Bohmann K, Gilbert MTP. Tag jumps illuminated - reducing sequence-to-sample misidentifications in metabarcoding studies. Mol Ecol Resour. 2015;15:1289–303.

122. Hanna RE, Doench JG. A case of mistaken identity. Nat Biotechnol. 2018;36:802–4.

123. MacConaill LE, Burns RT, Nag A, Coleman HA, Slevin MK, Giorda K, et al. Unique, dual-indexed sequencing adapters with UMIs effectively eliminate index cross-talk and significantly improve sensitivity of massively parallel sequencing. BMC Genomics. 2018;19:1–10.

124. Bartram J, Mountjoy E, Brooks T, Hancock J, Williamson H, Wright G, et al. Accurate Sample Assignment in a Multiplexed, Ultrasensitive, High-Throughput Sequencing Assay for Minimal Residual Disease. J Mol Diagnostics. 2016;18:494–506.

125. Goodwin S, McPherson JD, McCombie WR. Coming of age: Ten years of next-generation sequencing technologies. Nat Rev Genet. 2016;17:333–51.

126. van Dijk EL, Jaszczyszyn Y, Naquin D, Thermes C. The Third Revolution in Sequencing Technology. Trends Genet. 2018;34:666–81.

127. Benítez-Páez A, Sanz Y. Multi-locus and long amplicon sequencing approach to study microbial diversity at species level using the MinION™ portable nanopore sequencer. Gigascience. 2017;6:1–12.

128. Callahan BJ, Wong J, Heiner C, Oh S, Theriot CM, Gulati AS, et al. High-throughput amplicon sequencing of the full-length 16S rRNA gene with single-nucleotide resolution. bioRxiv. 2018;392332.

129. Hebert PDN, Braukmann TWA, Prosser SWJ, Ratnasingham S, DeWaard JR, Ivanova N V., et al. A Sequel to Sanger: Amplicon sequencing that scales. BMC Genomics. 2018;19:1–14.

130. Calus ST, Ijaz UZ, Pinto AJ. NanoAmpli-Seq: A workflow for amplicon sequencing for mixed microbial communities on the nanopore sequencing platform. Gigascience. 2018;giy140.

131. Volden R, Palmer T, Byrne A, Cole C, Schmitz RJ, Green RE, et al. Improving nanopore read accuracy with the R2C2 method enables the sequencing of highly multiplexed full-length single-cell cDNA. Proc Natl Acad Sci. 2018;115:9726–31.

132. Murray DC, Coghlan ML, Bunce M. From benchtop to desktop: Important considerations when designing amplicon sequencing workflows. PLoS One. 2015;10:1–21.

133. Scott R, Zhan A, Brown EA, Chain FJJ, Cristescu ME, Gras R, et al. Optimization and performance testing of a sequence processing pipeline applied to detection of nonindigenous species. Evol Appl. 2018;891–905.

134. Anslan S, Bahram M, Hiiesalu I, Tedersoo L. PipeCraft: Flexible open-source toolkit for bioinformatics analysis of custom high-throughput amplicon sequencing data. Mol Ecol Resour. 2017;17:e234–40.

135. Hildebrand F, Tadeo R, Voigt AY, Bork P, Raes J. LotuS: An efficient and user-friendly OTU processing pipeline. Microbiome. 2014;2:1–7.

136. Caporaso JG, Kuczynski J, Stombaugh J, Bittinger K, Bushman FD, Costello EK, et al. QIIME allows analysis of high-throughput community sequencing data. Nat. Methods. 2010. p. 335–6.

137. Edgar RC, Flyvbjerg H. Error filtering, pair assembly and error correction for next-generation sequencing reads. Bioinformatics. 2015;31:3476–82.

138. Schirmer M, Ijaz UZ, D'Amore R, Hall N, Sloan WT, Quince C. Insight into biases and sequencing errors for amplicon sequencing with the Illumina MiSeq platform. Nucleic Acids Res. 2015;43.

139. Nguyen NH, Smith D, Peay K, Kennedy P. Parsing ecological signal from noise in next generation amplicon sequencing. New Phytol. 2015;205:1389–93.

140. Bokulich NA, Subramanian S, Faith JJ, Gevers D, Gordon JI, Knight R, et al. Quality-filtering vastly improves diversity estimates from Illumina amplicon sequencing. Nat Methods. 2013;10:57–9.

141. Elbrecht V, Hebert PDN, Steinke D. Slippage of degenerate primers can cause variation in amplicon length. Sci Rep. 2018;8:1–5.

142. Kopylova E, Navas-Molina JA, Mercier C, Xu ZZ, Mahé F, He Y, et al. Open-Source Sequence Clustering Methods Improve the State Of the Art. mSystems. 2016;1:e00003-15.

143. Virgilio M, Backeljau T, Nevado B, De Meyer M. Comparative performances of DNA barcoding across insect orders. BMC Bioinformatics. 2010;11.

144. Callahan BJ, McMurdie PJ, Holmes SP. Exact sequence variants should replace operational taxonomic units in marker-gene data analysis. ISME J. 2017;11:2639–43.

145. Majaneva M, Hyyti?inen K, Varvio SL, Nagai S, Blomster J. Bioinformatic amplicon read processing strategies strongly affect eukaryotic diversity and the taxonomic composition of communities. PLoS One. 2015;10:1–18.

146. Tedersoo L, Ramirez KS, Nilsson RH, Kaljuvee A, Kõljalg U, Abarenkov K. Standardizing metadata and taxonomic identification in metabarcoding studies. Gigascience. 2015;4:1–4.

147. Callahan BJ, McMurdie PJ, Rosen MJ, Han AW, Johnson AJA, Holmes SP. DADA2: High-resolution sample inference from Illumina amplicon data. Nat Methods. 2016;13:581–3.

148. Amir A, Daniel M, Navas-Molina J, Kopylova E, Morton J, Xu ZZ, et al. Deblur Rapidly Resolves Single-Nucleotide Community Sequence Patterns. mSystems. 2017;2:e00191-16.

149. Edgar RC. UNOISE2: improved error-correction for Illumina 16S and ITS amplicon sequencing. bioRxiv. 2016;081257.

150. Elbrecht V, Vamos EE, Steinke D, Leese F. Estimating intraspecific genetic diversity from community DNA metabarcoding data. PeerJ. 2018;6:e4644.

151. Smyth RP, Schlub TE, Grimm A, Venturi V, Chopra A, Mallal S, et al. Reducing chimera formation during PCR amplification to ensure accurate genotyping. Gene. 2010;469:45–51.

152. Edgar RC, Haas BJ, Clemente JC, Quince C, Knight R. UCHIME improves sensitivity and speed of chimera detection. Bioinformatics. 2011;27:2194–200.

153. Haas BJ, Gevers D, Earl AM, Feldgarden M, Ward D V, Giannoukos G, et al. Chimeric 16S rRNA Sequence Formation and Detection in Sanger and 454-Pyrosequenced PCR Amplicons. Genome Res. 2011;21:494–504.

154. Brown EA, Chain FJJ, Crease TJ, Macisaac HJ, Cristescu ME. Divergence thresholds and divergent biodiversity estimates: Can metabarcoding reliably describe zooplankton communities? Ecol Evol. 2015;5:2234–51.

155. Decelle J, Romac S, Sasaki E, Not F, Mahé F. Intracellular diversity of the V4 and V9 regions of the 18S rRNA in marine protists (radiolarians) assessed by high-throughput sequencing. PLoS One. 2014;9.

156. Altschul SF, Gish W, Miller W, Myers EW, Lipman DJ. Basic local alignment search tool. J Mol Biol. 1990;215:403–10.

157. Huson D, Auch A, Qi J, Schuster S. MEGAN analysis of metagenome data. Genome Res. 2007;17:377–86.

158. Bengtsson-Palme J, Hartmann M, Eriksson KM, Pal C, Thorell K, Larsson DGJ, et al. metaxa2: Improved identification and taxonomic classification of small and large subunit rRNA in metagenomic data. Mol Ecol Resour. 2015;15:1403–14.

159. Koski LB, Golding GB. The closest BLAST hit is often not the nearest neighbor. J Mol Evol. 2001;52:540–2.

160. Janssen S, McDonald D, Gonzalez A, Navas-Molina JA, Jiang L, Xu ZZ, et al. Phylogenetic Placement of Exact Amplicon Sequences Improves Associations with Clinical Information.

mSystems. 2018;3:e00021-18.

161. Krause L, Diaz NN, Goesmann A, Kelley S, Nattkemper TW, Rohwer F, et al. Phylogenetic classification of short environmental DNA fragments. Nucleic Acids Res. 2008;36:2230–9.

162. Matsen FA, Kodner RB, Armbrust EV. pplacer: linear time maximum-likelihood and Bayesian phylogenetic placement of sequences onto a fixed reference tree. BMC Bioinformatics. 2010;11:538.

163. Berger SA, Krompass D, Stamatakis A. Performance, accuracy, and web server for evolutionary placement of short sequence reads under maximum likelihood. Syst Biol. 2011;60:291–302.

164. Mirarab S, Nguyen N, Warnow T. SEPP: SATé-Enabled Phylogenetic Placement. Pac Symp Biocomput. 2012. p. 247–58.

165. McDonald D, Price MN, Goodrich J, Nawrocki EP, Desantis TZ, Probst A, et al. An improved Greengenes taxonomy with explicit ranks for ecological and evolutionary analyses of bacteria and archaea. ISME J. 2012;6:610–8.

166. Bokulich NA, Kaehler BD, Rideout JR, Dillon M, Bolyen E, Knight R, et al. Optimizing taxonomic classification of marker-gene amplicon sequences with QIIME 2 ' s q2-feature-classifier plugin. Microbiome. 2018;6:90.

167. Wang Q, Garrity GM, Tiedje JM, Cole JR. Na??ve Bayesian classifier for rapid assignment of rRNA sequences into the new bacterial taxonomy. Appl Environ Microbiol. 2007;73:5261–7.

168. Pires DEV, Oliveira FS, Correa FB, Morais DK, Fernandes GR. TAG.ME: Taxonomic Assignment of Genetic Markers for Ecology. bioRxiv. 2018;263293.

169. De Queiroz K. Different species problems and their resolution. BioEssays. 2005;27:1263–9.

170. Porter TM, Gibson JF, Shokralla S, Baird DJ. Rapid and accurate taxonomic classification of insect (class Insecta) cytochrome c oxidase subunit 1 (COI) DNA barcode sequences using a naive Bayesian classifier. 2014;1:1–14.

171. Wang Q, Garrity GM, Tiedje JM, Cole JR. Naive Bayesian classifier for rapid assignment of rRNA sequences into the new bacterial taxonomy. Appl Environ Microbiol. 2007;73:5261–7.

172. Lan Y, Wang Q, Cole JR, Rosen GL. Using the RDP classifier to predict taxonomic novelty and reduce the search space for finding novel organisms. PLoS One. 2012;7:1–15.

173. Ficetola GF, Taberlet P, Coissac E. How to limit false positives in environmental DNA and metabarcoding? Mol Ecol Resour. 2016;16:604–7.

174. Elbrecht V, Steinke D. Scaling up DNA metabarcoding for freshwater macrozoobenthos monitoring. Freshw Biol. 2018;1–8.

175. Klymus KE, Marshall NT, Stepien CA. Environmental DNA (eDNA) metabarcoding assays to detect invasive invertebrate species in the Great Lakes. PLoS One. 2017;12:1–24.

176. Wilson CC, Wozney KM, Smith CM. Recognizing false positives: Synthetic oligonucleotide controls for environmental DNA surveillance. Methods Ecol Evol. 2016;7:23–9.

177. Hatzenbuhler C, Kelly JR, Martinson J, Okum S, Pilgrim E. Sensitivity and accuracy of high-throughput metabarcoding methods for early detection of invasive fish species. Sci Rep.

2017;7:1–10.

178. Mata VA, Rebelo H, Amorim F, Mccracken GF, Jarman S, Beja P. How much is enough? Effects of technical and biological replication on metabarcoding dietary analysis. Mol Ecol. 2018;

179. Ficetola GF, Pansu J, Bonin A, Coissac E, Giguet-Covex C, De Barba M, et al. Replication levels, false presences and the estimation of the presence/absence from eDNA metabarcoding data. Mol Ecol Resour. 2015;15:543–56.

180. Krehenwinkel H, Fong M, Kennedy S, Huang EG, Noriyuki S, Cayetano L, et al. The effect of DNA degradation bias in passive sampling devices on metabarcoding studies of arthropod communities and their associated microbiota. PLoS One. 2018;13:1–14.

181. Berec L, Kean JM, Epanchin-Niell R, Liebhold AM, Haight RG. Designing efficient surveys: spatial arrangement of sample points for detection of invasive species. Biol Invasions. 2014;17:445–59.

182. PM 7/98 (2) Specific requirements for laboratories preparing accreditation for a plant pest diagnostic activity. EPPO Bull. 2010;44:117–47.

183. National Association of Testing Authorities. Guidelines for the validation and verification of quantitative and qualitative test methods. 2012.

184. Schlaberg R, Chiu CY, Miller S, Procop GW, Weinstock G. Validation of metagenomic next-generation sequencing tests for universal pathogen detection. Arch Pathol Lab Med. 2017;141:776–86.

185. Gargis AS, Kalman L, Lubin IM. Assuring the quality of next-generation sequencing in clinical microbiology and public health laboratories. J Clin Microbiol. 2016;54:2857–65.

186. Adams IP, Fox A, Boonham N, Massart S, De Jonghe K. The impact of high throughput sequencing on plant health diagnostics. Eur J Plant Pathol. 2018;1–11.

187. Olmos A, Boonham N, Candresse T, Gentit P, Giovani B, Kutnjak D, et al. High-throughput sequencing technologies for plant pest diagnosis: challenges and opportunities. EPPO Bull. 2018;48:219–24.

188. Bell KL, Burgess KS, Botsch JC, Dobbs EK, Read TD, Brosi BJ. Quantitative and qualitative assessment of pollen DNA metabarcoding using constructed species mixtures. Mol Ecol. 2018;0–2.

189. Smith DP, Peay KG. Sequence depth, not PCR replication, improves ecological inference from next generation DNA sequencing. PLoS One. 2014;9.

190. Massart S, Candresse T, Gil J, Lacomme C, Predajna L, Ravnikar M, et al. A framework for the evaluation of biosecurity, commercial, regulatory, and scientific impacts of plant viruses and viroids identified by NGS technologies. Front Microbiol. 2017;8.

191. Carew ME, Coleman RA, Hoffmann AA. Can non-destructive DNA extraction of bulk invertebrate samples be used for metabarcoding? PeerJ. 2018;6:e4980.

192. Zizka VMA, Leese F, Peinert B, Geiger MF. DNA metabarcoding from sample fixative as a quick and voucher preserving biodiversity assessment method. bioRxiv [Internet]. 2018;287276. Available from: https://www.biorxiv.org/content/early/2018/03/23/287276

193. Hardwick SA, Chen WY, Wong T, Kanakamedala BS, Deveson IW, Ongley SE, et al. Synthetic microbe communities provide internal reference standards for metagenome sequencing and analysis. Nat Commun. 2018;9:3096.

194. Duncavage EJ, Abel HJ, Pfeifer JD. In Silico Proficiency Testing for Clinical Next-Generation Sequencing. J Mol Diagnostics. 2017;19:35–42.

195. Hardwick SA, Deveson IW, Mercer TR. Reference standards for next-generation sequencing. Nat Rev Genet. 2017;18:473–84.

196. Sinha R, Abu-Ali G, Vogtmann E, Fodor AA, Ren B, Amir A, et al. Assessment of variation in microbial community amplicon sequencing by the Microbiome Quality Control (MBQC) project consortium. Nat Biotechnol. 2017;35:1077–86.

197. Schrijver I, Aziz N, Jennings LJ, Richards CS, Voelkerding K V., Weck KE. Methods-based proficiency testing in molecular genetic pathology. J Mol Diagnostics. 2014;16:283–7.

198. Knight R, Vrbanac A, Taylor BC, Aksenov A, Callewaert C, Debelius J, et al. Best practices for analysing microbiomes. Nat Rev Microbiol. 2018;16:410–22.

199. Schlick-Steiner BC, Steiner FM, Seifert B, Stauffer C, Christian E, Crozier RH. Integrative Taxonomy: A Multisource Approach to Exploring Biodiversity. Annu Rev Entomol. 2010;55:421–38.

200. Yilmaz P, Kottmann R, Field D, Knight R, Cole JR, Amaral-Zettler L, et al. Minimum information about a marker gene sequence (MIMARKS) and minimum information about any (x) sequence (MIxS) specifications. Nat Biotechnol. 2011;29:415–20.

201. Stephens ZD, Lee SY, Faghri F, Campbell RH, Zhai C, Efron MJ, et al. Big data: Astronomical or genomical? PLoS Biol. 2015;13:1–11.

202. Evans DM, Kitson JJN, Lunt DH, Straw NA, Pocock MJO. Merging DNA metabarcoding and ecological network analysis to understand and build resilient terrestrial ecosystems. Funct Ecol. 2016;1904–16.

203. Lafleur JP, Jönsson A, Senkbeil S, Kutter JP. Recent advances in lab-on-a-chip for biosensing applications. Biosens Bioelectron. 2016;76:213–33.

204. Potamitis I, Eliopoulos P, Rigakis I. Automated Remote Insect Surveillance at a Global Scale and the Internet of Things. Robotics. 2017;6:19.

205. Bohan DA, Vacher C, Tamaddoni-Nezhad A, Raybould A, Dumbrell AJ, Woodward G. Next-Generation Global Biomonitoring: Large-scale, Automated Reconstruction of Ecological Networks. Trends Ecol Evol. 2017;32:477–87.

206. Daborn PJ. A Single P450 Allele Associated with Insecticide Resistance in Drosophila. Science (80-). 2002;297:2253–6.

207. Stapley J, Santure AW, Dennis SR. Transposable elements as agents of rapid adaptation may explain the genetic paradox of invasive species. Mol Ecol. 2015;24:2241–52.

208. Ricciardi A, Blackburn TM, Carlton JT, Dick JTA, Hulme PE, Iacarella JC, et al. Invasion Science: A Horizon Scan of Emerging Challenges and Opportunities. Trends Ecol Evol. 2017;32:464–74.

209. Bergqvist J, Forsman O, Larsson P, Näslund J, Lilja T, Engdahl C, et al. Detection and Isolation of Sindbis Virus from Mosquitoes Captured During an Outbreak in Sweden, 2013. Vector-Borne Zoonotic Dis. 2015;15:133–40.

210. Muschelli J. rscopus: Scopus Database "API" Interface [Internet]. 2018. Available from: https://github.com/muschellij2/rscopus

211. R Core Team. R: A language and environment for statistical computing. [Internet]. R Foundation for Statistical Computing, Vienna, Austria.; 2017. Available from: http://www.r-project.org/

212. Wickham H. ggplot2: Elegant Graphics for Data Analysis [Internet]. Springer-Verlag New York; 2016. Available from: http://ggplot2.org

213. Plant Health Australia. The National Plant Biosecurity Status Report. 2017.

214. Chamberlain S. bold: Interface to Bold Systems API [Internet]. 2017. Available from: https://cran.r-project.org/package=bold

215. Winter DJ. rentrez: An R package for the NCBI eUtils API. R J. 2017;9:520–6.

216. Schöfl G. biofiles: An Interface for GenBank/GenPept Flat Files [Internet]. Available from: https://github.com/gschofl/biofiles

217. Kahle D, Wickham H. ggmap: Spatial Visualization with ggplot2. R J [Internet]. 2013;5:144–61. Available from: http://journal.r-project.org/archive/2013-1/kahle-wickham.pdf

TABLES

Table 3. Recommended quality control checkpoints for metabarcoding based diagnostics.

	Quality control checkpoint	Consequences
**Laboratory preparedness**	Are all reagents within expiry date and stored properly?	Poor reagent storage can lead to reduced efficiency and false negatives
	Is equipment appropriately maintained and calibrated?	Poorly calibrated equipment will generate inconstancies and inaccurate data
	Have laboratory surfaces been decontaminated?	Dirty laboratories can be a source of DNA contamination, leading to lowered sensitivity or false positives
**Sample acceptance**	Have specimens arrived in a condition appropriate for extracting DNA?	Inappropriately stored specimens can lead to false negative results and a reduction in sensitivity
	Are specimens traceable to origin location?	Misidentification of sample origin can complicate detection response
**Nucleic acid extraction**	Is DNA of sufficient quantity and quality?	Insufficient DNA quantity or presence of contaminants can inhibit reactions and result in false negatives
**Marker enrichment**	Are the correct fragment sizes present for the target barcode marker?	Incorrect fragment sizes could indicate off-target amplification
	Have the positive control samples successfully amplified?	Absence of product in positive controls indicates amplification failure
	Are negative control samples free of DNA fragments?	Visible DNA fragments in negative controls indicates contamination
**Library preparation & multiplexing**	Are libraries of the appropriate size and concentration?	Libraries of significantly different sizes or concentrations will complicate multiplexing
	Have sets of unique-dual indices been used?	Unique-dual indexing is necessary to control

		for index-switching
	Have index sets been alternated since the previous sequencing run?	Cross-contamination of libraries between sequencing runs can cause false positives
**High-throughput sequencing**	Has the pooled library been appropriately sized and quantified?	Inaccurate sizing and quantification can cause overloading of flow cell and failed runs, or underloading and low data output
	Has the sequencer been appropriately cleaned between runs?	Insufficient cleaning of the sequencer can result in cross-contamination between runs
**Demultiplexing & quality trimming**	Has minimum sequencing depth been achieved for each sample?	Low sequencing depth can cause false positives
	Are an appropriate number of reads passing quality filtering?	Low numbers of reads passing quality filters can indicate issues with sequencing run and result in false negatives
**OTU Clustering & Denoising**	How much of the original data is explained by the final OTU's	Lower than expected sequences can indicate overly restrictive bioinformatics parameters
	Have chimeras been and sequences with disrupted ORF's been checked for? (for protein coding genes)	Chimeras and pseudogenes can inflate taxonomic diversity lead to false positives
**Taxonomic assignment**	Has the reference database been curated to remove mislabelled taxonomy and pseudogenic sequences?	Mislabelled sequences can lead to false positives & negatives
	Has the taxonomy been applied with appropriate confidence levels?	Low confidence assignment indicates incomplete or issues in reference database
**Interpretation of results**	Have the taxa received an appropriate number of reads to pass detection threshold?	Taxa under detection threshold could represent errors that have not been sufficiently controlled for
	Has a minimum detection threshold been applied to remove index-switching?	Index-switching can cause spreading of taxa to other samples and result in false positives
	Are there any taxa that need to be confirmed with alternative methods?	Any putative detections or low confidence detections should be confirmed with alternative method before reporting
**Reporting & sign off**	Have any exceptions to laboratory standard operating procedure been made?	Non-compliances with SOP should be highlighted and diagnostic confidence may be reduced
	Has data been stored appropriately?	Archiving of data is important for re-analysis
	Have results been signed off by competent individual?	Taxa of significance may be interpreted or presented incorrectly, leading to incorrect biosecurity response

Figure Legends

Figure 1- Metabarcoding in the literature

A. Scopus search of all metabarcoding studies, and those containing words in abstract title or keywords relevant to invasive insect surveillance. **B.** Sequencing platforms used in metabarcoding studies displayed as a proportion for each year.

Figure 2- Overview of common metabarcoding workflows for identification of trapped insect species

Figure 3- DNA barcodes on public reference databases

A. Global distribution of all sufficiently annotated DNA barcode records from BOLD and GenBank; Insecta are represented in green, while those species present on international pest lists are represented in red. **B**. Distribution of records and **C**. species within major public databases

for the 10 barcode markers with the most reference information for entire Insecta and pest Insecta.

Figure 4- Unique dual indexing overcomes issues of cross-contamination due to index-switching

A. An amplified barcode locus with sequencing adapters attached, read locations and orientations are indicated for commonly used Illumina MiSeq platform. Read 1 and 2 are designed to overlap to facilitate assembly into a consensus sequence. Both sequencing adapters incorporate a unique oligonucleotide index sequence to allow differentiation of multiplexed samples. Strategies for indexing include; **B.** Combinatorial indexing, where indices on either end of the molecule are shared with other samples but the combination of the two is unique, and **C.** Unique dual indexing, where adapter indices at both ends of the molecule are completely unique to the sample.

Figure 1

Click here to download Figure Fig1_Final.pdf

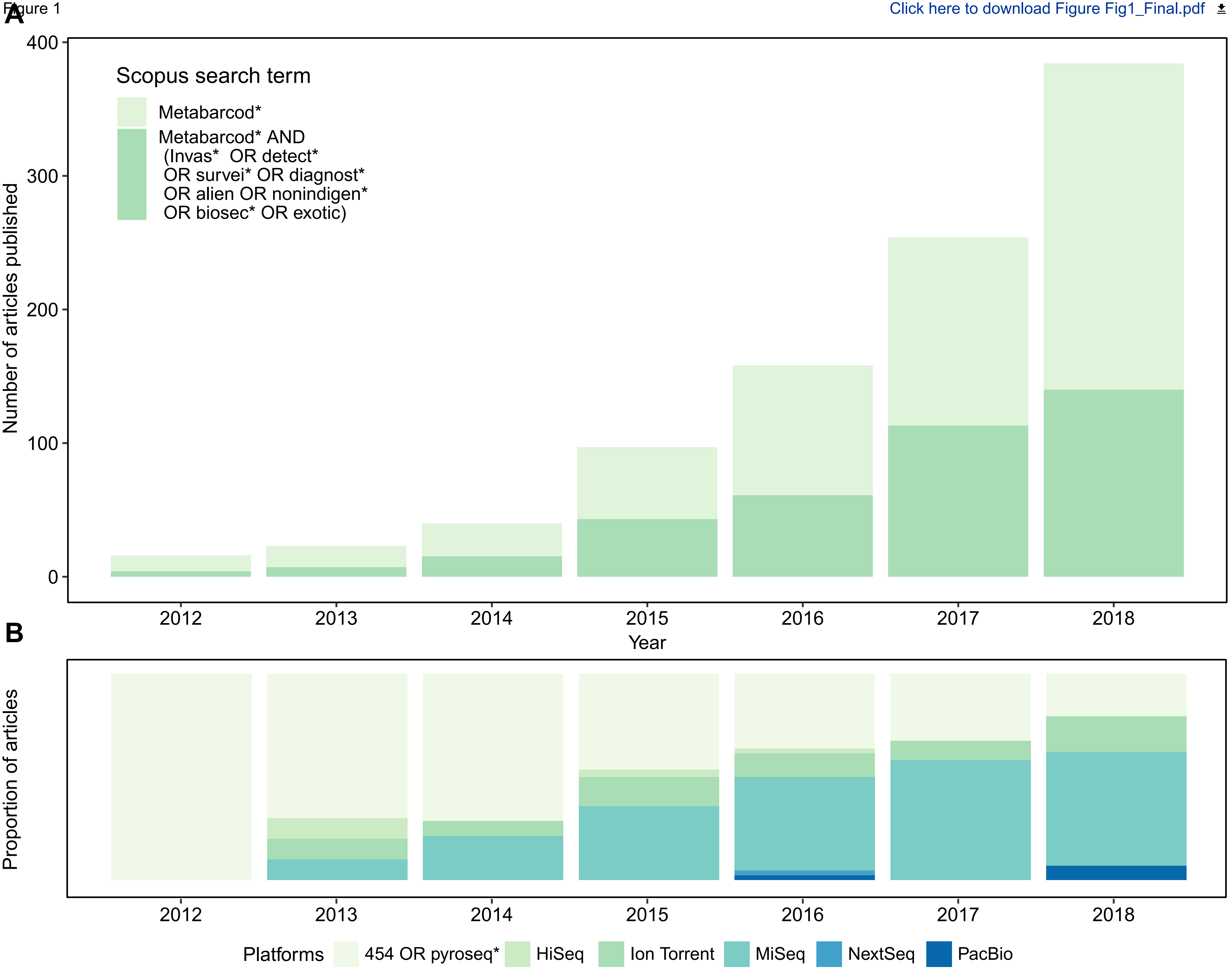

Figure 2

Click here to download Figure Fig2_Final.pdf

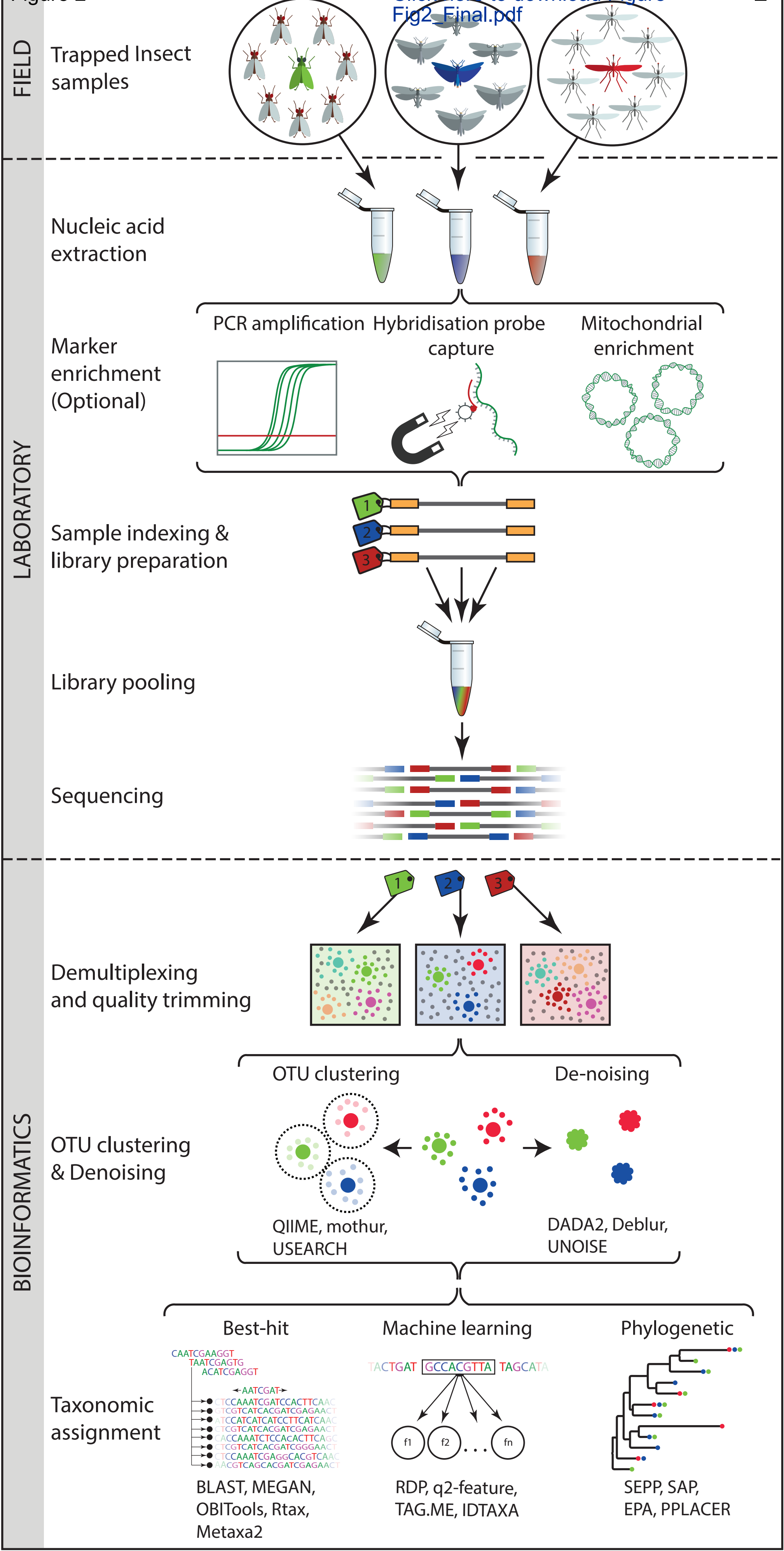

Figure 3

Click here to download Figure Fig3_Final_Rastered.pdf

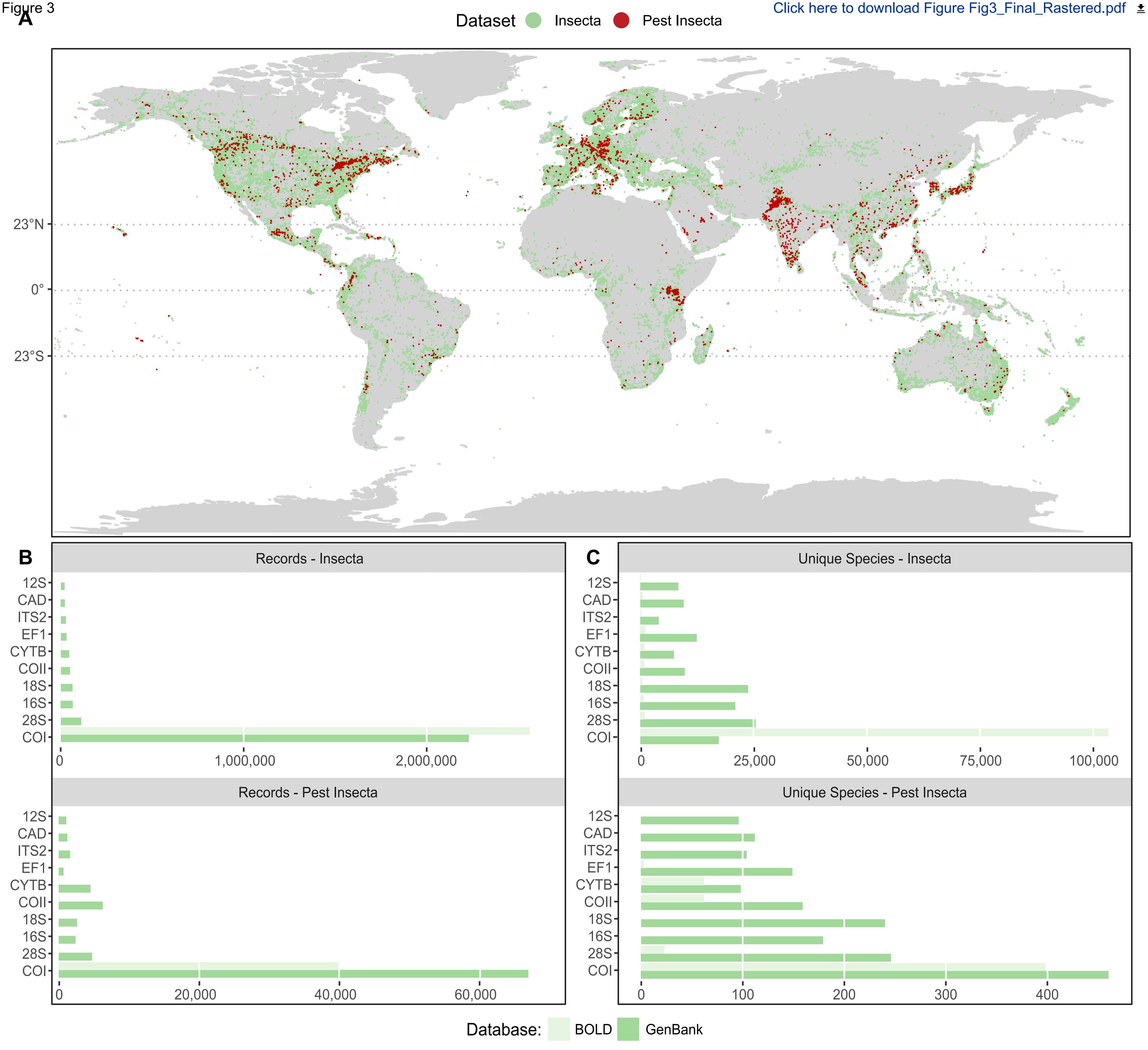

Figure 4 Click here to download Figure Fig4_Final.pdf

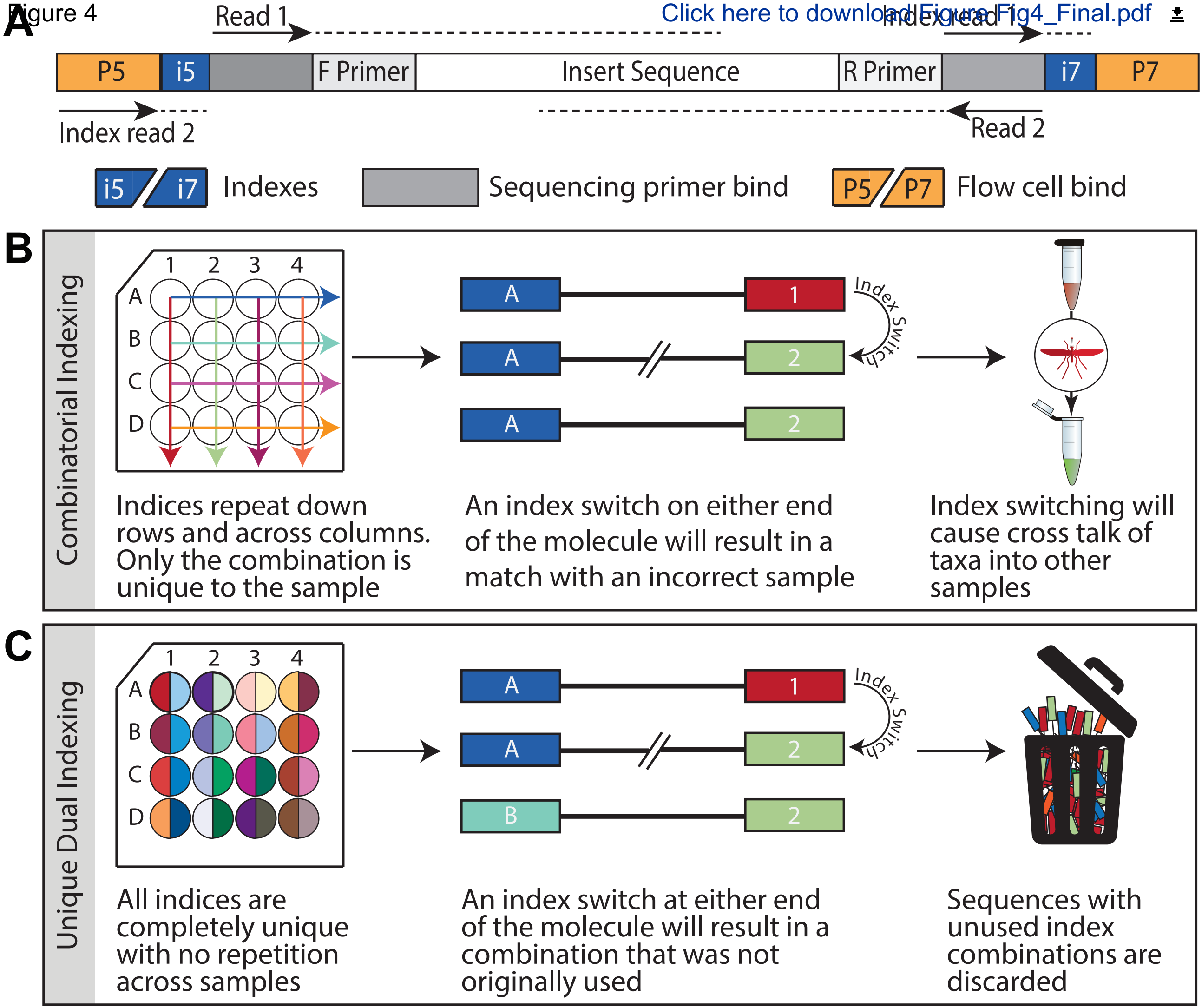

Code to implement analyses

Click here to access/download
Supplementary Material
Supplementary_S1.pdf
