# Supplementary material for: Prospects and challenges of implementing DNA metabarcoding for high-throughput insect surveillance
Source: Gigascience. 2019 Jul 30;8(8):giz092. doi: 10.1093/gigascience/giz092 (PMC6667344; doi:10.1093/gigascience/giz092)
Supplement: giz092_GIGA-D-19-00011_Revision_1 [file giz092_giga-d-19-00011_revision_1.pdf]

GigaScience

Prospects and challenges of implementing DNA metabarcoding for high throughput insect surveillance

--Manuscript Draft--

Manuscript Number:	GIGA-D-19-00011R1
Full Title:	Prospects and challenges of implementing DNA metabarcoding for high throughput insect surveillance
Article Type:	Review
Funding Information:	Plant Biosecurity Cooperative Research Centre (2153) — Dr John Paul Cunningham<br>Horticulture Innovation Australia (ST16010) — Dr Brendan C. Rodoni<br>State Government of Victoria (CMI105584) — Dr Mark J. Blacket
Abstract:	Trap based surveillance strategies are widely employed for monitoring of invasive insect species, aiming to detect newly arrived exotic taxa as well as track the population levels of established or endemic pests. Where these surveillance traps have low specificity and capture non-target endemic species in excess of the target pests, the need for extensive specimen sorting and identification creates a major diagnostic bottleneck. While the recent development of standardised molecular diagnostics has partly alleviated this requirement; the single specimen per reaction nature of these methods does not readily scale to the sheer number of insects trapped in surveillance programmes. Consequently, target lists are often restricted to a few high-priority pests, allowing unanticipated species to avoid detection and potentially establish populations.<br><br>DNA metabarcoding has recently emerged as a method for conducting simultaneous, multi-species identification of complex mixed communities, and may lend itself ideally to rapid diagnostics of bulk insect trap samples. Moreover, the high-throughput nature of recent sequencing platforms could enable the multiplexing of hundreds of diverse trap samples on a single flow cell, thereby providing the means to dramatically scale-up insect surveillance in terms of both the quantity of traps that can be processed concurrently, and number of pest species that can be targeted. In this review of the metabarcoding literature, we explore how DNA metabarcoding could be tailored to the detection of invasive insects in a surveillance context and highlight the unique technical and regulatory challenges that must be considered when implementing high-throughput sequencing technologies into sensitive diagnostic applications.
Corresponding Author:	Alexander M Piper, B.Sc.<br>La Trobe University<br>Bundoora, VIC AUSTRALIA
Corresponding Author Secondary Information:	
Corresponding Author's Institution:	La Trobe University
Corresponding Author's Secondary Institution:	
First Author:	Alexander M Piper, B.Sc.
First Author Secondary Information:	
Order of Authors:	Alexander M Piper, B.Sc.
	Jana Batovska, B.Sc. (Hons)
	Noel O.I. Cogan, PhD
	John Weiss, PhD
	John Paul Cunningham, PhD

Powered by Editorial Manager® and ProduXion Manager® from Aries Systems Corporation

	Brendan C. Rodoni, PhD
	Mark J. Blacket, PhD
**Order of Authors Secondary Information:**	
**Response to Reviewers:**	Dear Editor,<br>We thank the reviewers for their constructive and helpful comments. Since our previous submission our manuscript has been updated to incorporate the reviewers' suggestions, the particulars of which we have listed below:<br><br>•We have expanded the breadth of our literature search, including further references uncovered through this process and those suggested by the reviewers.<br>•We have updated Figure 1 to reflect this expanded search, and it now includes citations from Crossref and Pubmed in addition to Scopus.<br>•We have condensed the discussion of alternative loci, as well as PCR free approaches and instead refer to in-depth reviews on the topics.<br>•We have added discussion of the limitations of multi-locus approaches in relation to assigning taxonomy and added an additional box that covers the use of multi-locus approaches for capturing markers beyond taxonomic inference, for example the parallel identification of vectored pathogens in modular metabarcoding assays.<br>•We have further covered the MGI/BGI sequencers in our discussion of sequencing platforms and Table 2 as per your suggestion and also have covered the recently released PacBio Sequel II.<br>•We have added additional detail in the Background and throughout the manuscript on the implications of barcoding for biosecurity law<br>•We have revised the taxonomic assignment and technical replicates sections to address important advances highlighted by the reviewers.<br>•Finally, we have updated Figure 2 to summarise our vision for how metabarcoding can be applied to insect pest surveillance and to include further aspects discussed in this manuscript such as technical replicates, controls, long read sequencing, additional loci, and interpretation of results.<br>We believe these changes address the primary concerns of the reviewers. Please find detailed responses to the specific comments of the reviewers below.<br><br><br>Reviewer #1: Piper et al. present a thorough review on DNA metabarcoding applied to high throughput insect surveillance. The text is well written and it does not contain any major flaw, so I think it is a good contribution to the literature. The authors should nevertheless be aware that similar reviews -although not dedicated to insect surveillance- already exist in the bibliography (two recent examples that the authors omitted: Deiner et al. 2017 Mol Ecol; Alberdi et al. 2019 Mol Ecol Res). These reviews cover some redundant issues, which could enable reducing the length of this article in some sections, but also contain some contents and ideas that have not been added to this article and might be worth referencing in the context of high throughput insect surveillance (e.g. using replicated restrictively to gain confidence on the identified taxa and reduce the risk of false positives).<br><br>Response: We have highlighted other reviews of the wider metabarcoding literature in the background, including the suggested references. In the quality assurance section of the manuscript we have further expanded on some of the ideas suggested in these references, including the benefits of additive or restrictive processing of replicates.<br><br><br>Reviewer #2: The manuscript is a review on the usage metabarcoding in the context of insect control, biosurveillance, and with a special emphasis on pests. It is an important contribution for control agencies around the world who require more throughput in their analysis of invasive species that can be carried in imports and movement of goods. It is also a good contribution to entomologists and molecular biologists looking to incorporate metabarcoding of these taxa into their research. The review includes mostly all steps involved in metabarcoding for surveillance purposes, from selecting a marker to quality assurance. In my opinion, the paper is presented in a coherent and organized manner. However, there are a few points that I consider will make the manuscript stronger, and that it would widen the reach of the paper. I would like to see a wider scope in their searching method (there are a few references missing that I think

will improve the manuscript). I also will like to see thorough discussion on the current state of affairs in international law about biosecurity and pest management , since this directly affect the applicability of barcodes in this context. Finally, the manuscript will benefit to have a figure perspectives and conclusions that summarize the author's vision on how to apply barcodes for bio-surveillance. Below more specific comments:

Response: We have expanded our literature search beyond the Scopus database and added further relevant references that were previously missed. We have added an additional paragraph to the introduction to address the current state of affairs in international biosecurity law since the introduction of the WTO agreement on Sanitary and Phytosanitary measures and provided additional detail throughout the manuscript where a particular aspect of the metabarcoding approach may be impacted by current regulatory frameworks. We agree that summarising our vision of how metabarcoding should be applied in a figure would benefit the manuscript. However, rather than adding an extra figure we have updated figure 2 to better summarise our vision for how samples a range of insect surveillance activities could be fed into the same core metabarcoding diagnostic assay, with the results informing relevant management actions. We have further updated this figure to include diagrams relating to quality control and long read sequencing.

* Typo in line 73
* Adjust Table2 column width
* In line 152 you make reference to figure 2 when you meant figure 3
* As before but in lines 167 and 168

Response: We thank the reviewer for bringing these mistakes to our attention and they have been corrected.

* It is not clear how can you obtain minimal amplification bias. This is not even clear in the cited articles, and one of them (ref. 83) even explicitly says that it requires in vitro validation and "as many primers might not be suitable for DNA metabardocing due to low base degeneracy, potentially high primer bias or critical design flaws". Therefore the sentences of lines 194-198 is a bit misleading and should be reworded, despite the following sentence introducing other sources of bias.

Response: We have reworded this to state: This bias is thought to mainly arise from primer-template mismatches, particularly at the 3' end of the primer where extension takes place (Piñol et al 2015, 2019) and therefore comprehensive in-silico evaluation should be conducted at the beginning of a project to ensure primer sequences are appropriate for the underlying target community (Rennstam Rubbmark et al 2018, Bylemans et al 2018 Ficetola et al 2010). Where mismatches with certain taxa are predicted to occur, inclusion of degenerate bases can overcome taxonomic bias inherent to a specific primer sequence (Elbrecht et al 2017), however high levels of degeneracy can also lead to undesirable off-target amplification or formation of dimers (Mioduchowska et al 2018, Marquina et al 2018) which will require further laboratory validation to detect (Clarke et al 2014, Elbrecht et al 2017)

* Despite the argument about avoiding PCR is compelling, I believe that the author's "over-endorsement" of the micro-array chips is a big leap. It is especially difficult to deliver the appropriate probes when good references are not present, as is one of the arguments of the paper.

Response: We have condensed this section and removed some of the discussion on microarrays and hybrid capture, instead referring to the in-depth reviews of Mamanova et al 2010 and Jones et al 2015 on the topic of PCR free targeted enrichment approaches. We have also raised a further consideration that implementation of PCR-free sequence enrichment may require overcoming further regulatory hurdles as opposed to the already wide acceptance of PCR amplification within diagnostic protocols.

* For an interesting discussion on index-switching (discussed around line 257), you can

Powered by Editorial Manager® and ProduXion Manager® from Aries Systems Corporation

include https://doi.org/10.1111/1755-0998.12928

Response: We thank the reviewer for highlighting this paper, and we have added the reference to the manuscript

* Despite that the authors mentioned different sequencing platforms, they seem to restrict their discussion of "demultiplexing and sequence quality trimming" to Illumina pair end reads. They should include at least a paragraph regarding the rest of the platforms discussed

Response: In the interest of article length, rather than adding an additional paragraph we have adapted the current paragraph to be more generic to all the sequencers discussed. Overall the process is reasonably similar between Illumina and other modern platforms, with the exception being the need to assemble consensus sequences prior to quality trimming if using PacBio CCS or another third-party consensus method for Nanopore. We have also highlighted that quality trimming using error profiles is a coarse filtering process where parameters should be carefully considered, particularly for higher error rate nanopore reads. We have further added diagrams in figure 2 to help highlight the differences between analysis of metabarcoding data from short and long read platforms.

* I think that referencing 150 in line 374 sounds like Elbrecht et al. 2018 used zero-radius OTUs (ZOTU) to investigate intraspecific variation. It is a bit misleading since they used a 3% threshold after the ZOTU inference, essentially making it not an ESV. Some clarification would be needed.

Response: We have clarified that use of denoising algorithms (rather than ESVs per se) provide the ability to investigate intra-specific variation as they don't impose the arbitrary similarity threshold which define OTUs. This single nucleotide resolution enables binning sequences into 'amplicon sequence variants' that retain precise haplotype information that can be necessary for diagnostics of closely related taxa or tracking an invasion and act as a consistent label between analyses. We now reference Marshal & Stephien 2019 who use the fine-scale resolution provided by ASV's to infer population histories of an invasive species.

* Fix reference 167

Response: We thank the reviewer for noticing the error in reference 167 and it has been corrected.

* The authors should be careful with the statement " makes the classification process more robust to pervasive issues of missing and mis-annotated data in reference database". While is true that some ML implementations give some sort of confidence levels in the taxonomic hierarchy, it is not true that it would be less influenced by misannoations and missing references. In fact, most implementations of the Naive bayes will return NA when no significant match with the training set is found. Also, mislabels in the training set will lead to erroneous prediction (https://doi.org/10.1613/jair.606) and is an ongoing challenge in ML to detect mislabels (https://doi.org/10.1007/978-3-319-58628-1_43)

* The statement "In cases where there may be ambiguity due to imperfect reference data and multiple taxonomic outcomes obtain similar probabilities, the sequence may still be robustly assigned to a higher taxonomic rank (e.g. family) [76], providing important information about sample composition and possible presence of novel taxa" is also problematic in my view. The text suggest that Naive bayes by itself has this property, which is not true. Only specific implementations and variations of the algorithm have it. The paper would benefit from exploring this further.

Response to both of the above: We agree with the reviewer that this section of the manuscript unintentionally conflates the specifics of the RDP implementation of naïve Bayes algorithm with the broader concept of probabilistic approaches to sequence

classification. We have now revised the taxonomic assignment section so that Paragraph 1 introduces the general approaches to sequence classification (Sequence similarity, sequence composition, phylogenetic, or a hybrid of these) and then refers to other reviews and comparisons of these methods. The remainder of paragraph 1 focuses on the problem of over-classification using the popular BLAST best-hit assignment method. Paragraph 2 now focuses more broadly on the importance of using methods of classification that return a confidence score, which is particularly important for metabarcoding applications where management decisions are based on appropriate assignment of a taxonomic name to sequence reads.

* Some important development is missing in the taxonomic assignment section. The probabilistic method Protax (DOI: 10.1093/bioinformatics/btw346,DOI: 10.1111/2041-210X.12721). Also Axtner et al. (doi: https://doi.org/10.1101/345082) preprint contains a very robust workflow for eDNA, and I think you can extract important information from their discussion. Protax do not require a complete database, gives hierarchical probability of assignment and has a very elegant mathematical framework. It would be a shame if it is not included in your review (or at least mention it to the public).

Response: We agree with the reviewer that we have overlooked the importance of PROTAX method for sequence classification. In paragraph 2 of the taxonomic assignment section we now introduce the PROTAX method and discuss its advantages, in particular the ability to model probability that a sequence belongs to species that exist in Linnaean taxonomy but not in the sequence reference database, as well as modelling the probability that a sequence comes from a taxa novel to both the reference sequence database and reference taxonomy. In box 1 we further highlight the ability of PROTAX and other methods to set a prior weighting for certain sequences, which could be useful when combining high confidence in-house sequences with more variable quality public sequences, or when the endemic diversity for the target region is well characterized.

*I disagree with the authors when they say "While the importance of technical replication for increasing detection probability is generally agreed upon [31,178,179], replicates reduce throughput without providing further independent data points [26]. Instead, with metabarcoding removing one of the major roadblocks to large-scale surveillance, more biological replicates from more frequent and intensive trapping could be used. Considering biological replication is particularly important as regardless of the effectiveness of the metabarcoding diagnostic assay if an insect is not caught in a trap it does not necessarily mean absence in the area". While I agree on the importance of biological replication (and my argument is never against that), technical replicates are of utmost important, especially when dealing with rare species and invasive species. Even if the entire labwork is done in pristine condition, with close to 0 bias, the resulting sequences vary dramatically between replicates. Also, technical replicates inform you of the overall quality of your survey, and allows you to model or correct for biases that might have occur. I think it would be a disservice to the community to let a statement that disregards technical replicates pass. Please consider revising this point.

Response: We agree with the reviewer that we have discounted the importance of technical replication too far. This section has been revised and we have emphasised the importance of technical replication to counter stochasticity during PCR and library preparation and identify laboratory cross-contamination in the case that replicates show significant dissimilarities in taxonomic composition. We then contrast additive and restrictive processing of replicates, and suggest it may be best to include a minimum number of technical replicates to allow a majority rules approach (i.e. 2/3 replicates count as a detection) to balance the positives of both approaches. We then suggest the use of site occupancy models to determine the appropriate number of both biological and technical replicates required to reach the desired statistical power for the survey.

* In line 541, there is something missing after "similar"

Powered by Editorial Manager® and ProduXion Manager® from Aries Systems Corporation

	Response: We have corrected this to read: "similar standardisation of metabarcoding protocols"<br><br>* While I liked and agree with the idea exposed between the lines 541 and 550, the references cited proposed in silico mock communities. Despite this is necessary, I think the authors should also push for the usage of a standard and diverse mock community. The analyses of in silico mock communities and the real mock communities have shown important differences between the two.<br><br>Response: We have suggested the use of standard and diverse mock communities, alongside computational datasets for benchmarking, and have given the example of the ZymoBIOMICS microbial mock community standards, which are used in the microbiome sequencing field to allow more realistic benchmarking of both laboratory and bioinformatics methods than can be provided by in-silico mock communities alone.<br><br>* Although Scopus is a large database, it missing a significant amount of citations. I would encourage the authors to widen their scope to other sources. R has a very good package called fulltext that encompasses multiple sources.<br><br>Response: The literature search has been expanded beyond Scopus and figure 1 has been recreated to also include citations listed on Crossref, and Pubmed, resulting in an addition of 16 citations for years prior to 2019. We have also included all 2019 publications to date, adding an additional 257 citations to this figure and resulting in the use of Nanopore sequencing and Illumina NovaSeq now being represented in Fig1b. The methods section and Supplementary 1 have been updated to include the new methods involved in the creation of this figure.<br><br>We thank the reviewers for their insightful and constructive comments, which have helped us improve the manuscript.
**Additional Information:**	
**Question**	**Response**
Are you submitting this manuscript to a special series or article collection?	No
**Experimental design and statistics**<br><br>Full details of the experimental design and statistical methods used should be given in the Methods section, as detailed in our Minimum Standards Reporting Checklist. Information essential to interpreting the data presented should be made available in the figure legends.<br><br>Have you included all the information requested in your manuscript?	Yes
**Resources**<br><br>A description of all resources used, including antibodies, cell lines, animals and software tools, with enough information to allow them to be uniquely	Yes

identified, should be included in the Methods section. Authors are strongly encouraged to cite Research Resource Identifiers (RRIDs) for antibodies, model organisms and tools, where possible.<br><br>Have you included the information requested as detailed in our Minimum Standards Reporting Checklist?	
**Availability of data and materials**<br><br>All datasets and code on which the conclusions of the paper rely must be either included in your submission or deposited in publicly available repositories (where available and ethically appropriate), referencing such data using a unique identifier in the references and in the “Availability of Data and Materials” section of your manuscript.<br><br>Have you have met the above requirement as detailed in our Minimum Standards Reporting Checklist?	Yes

Manuscript
Click here to view linked References
Click here to access/download;Manuscript;DNA_Metabarcoding_Insect_Sur

Prospects and challenges of implementing DNA metabarcoding for high throughput insect surveillance

Alexander M. Piper[1,2], Jana Batovska[1,2], Noel O.I. Cogan[1,2], John Weiss[1], John Paul Cunningham[1], Brendan C. Rodoni[1,2], Mark J. Blacket[1]

[1] Agriculture Victoria Research, AgriBio Centre, Bundoora 3083, Victoria, Australia

[2] School of Applied Systems Biology, La Trobe University, Bundoora 3083, Victoria, Australia

Corresponding author:

Alexander M. Piper

Email: alexander.piper@ecodev.vic.gov.au;

ORCID IDs:

Alexander M. Piper: 0000-0002-0664-7564

Jana Batovska: 0000-0002-0919-568X

John Paul Cunningham: 0000-0002-9348-2939

Mark J. Blacket: 0000-0001-7864-5712

Abstract

Trap based surveillance strategies are widely employed for monitoring of invasive insect species, aiming to detect newly arrived exotic taxa as well as track the population levels of established or endemic pests. Where these surveillance traps have low specificity and capture non-target endemic species in excess of the target pests, the need for extensive specimen sorting and identification creates a major diagnostic bottleneck. While the recent development of standardised molecular diagnostics has partly alleviated this requirement; the single specimen per reaction nature of these methods does not readily scale to the sheer number of insects trapped in surveillance programmes. Consequently, target lists are often restricted to a few high-priority pests, allowing unanticipated species to avoid detection and potentially establish populations.

DNA metabarcoding has recently emerged as a method for conducting simultaneous, multi-species identification of complex mixed communities, and may lend itself ideally to rapid diagnostics of bulk insect trap samples. Moreover, the high-throughput nature of recent sequencing platforms could enable the multiplexing of hundreds of diverse trap samples on a single flow cell, thereby providing the means to dramatically scale-up insect surveillance in terms of both the quantity of traps that can be processed concurrently, and number of pest species that can be targeted. In this review of the metabarcoding literature, we explore how DNA metabarcoding could be tailored to the detection of invasive insects in a surveillance context and highlight the unique technical and regulatory challenges that must be considered when implementing high-throughput sequencing technologies into sensitive diagnostic applications.

Keywords:

Biosecurity, Alien species, Biosurveillance, Early detection, Bioinformatics, Reference database, Quality assurance, Controls, Validation, Non-destructive

BACKGROUND

Increasing globalisation of trade and tourism along with changing climates are expected to further increase the rate of biological invasions over coming decades [1–3]. Insects form a dominant component of this global spread of invasive species [4], posing a major threat to agroecosystems [5], the environment [6] and human health [7] through disruption of ecological networks, plant herbivory and the transmission of pathogens and disease [8]. Once established in a new environment, ongoing containment and control of invasive insect pests imposes significant costs to industry, government and private landowners [8] and consequently major efforts are made to forecast incursion risk [9–11] and implement quarantine of entry pathways [12–14]. Despite these measures, the exponential increase in global movement of food, commerce, and humans complicates traceability and makes quarantine inspection of more than a fraction of arriving cargo an impossible task [15,16]. Therefore, proactive post-border surveillance within agricultural and natural landscapes is becoming an increasingly important component of effective biosecurity programmes, aiming to detect invasive species early before populations escalate or spread and eradication becomes unfeasible [17–19].

Insect invasions can initiate and disperse across vast and highly heterogenous landscapes [20], and therefore surveillance programmes often involve extensive trapping conducted across a range of spatial scales, from large geographic areas to precise crop monitoring activities within agricultural properties [21]. As it is generally unclear whether a new introduction has occurred, or what species it may be, surveillance programmes can extend over many years and target diverse taxonomic groups [22,23]. In many cases surveillance traps will capture non-target endemic species in vast excess of the target pests and the sheer number of specimens that need to be sorted through and identified by highly-trained entomologists forms a major diagnostic bottleneck. While insect diagnostics still largely relies on traditional morphological examination [24], in recent years this has been supplemented by a range of molecular techniques that allow

standardised identification of a wide range of taxa without specialist taxonomic expertise (Table 1). DNA barcoding in particular has become a central component of the modern diagnostic toolbox, due to the ability to compare a single unknown specimen against many potential species in a single assay, and standardised protocols that allow transparent and objective comparison of specimen identifications between laboratories, regulatory agencies and trading partners [24–26]. Despite these advantages, the time-consuming process of conducting single PCR and sequencing reactions on individual specimens has restricted the use of DNA barcoding to confirming the identity of specimens already deemed suspect by prior morphological sorting, or for identification of taxa or life stages where a taxonomic key may not be available or key diagnostic structures are degraded or missing [24,27]. Without access to a scalable and cost effective diagnostic method for large trap catches, current surveillance programmes generally do not identify all specimens to species level [23,28]. Instead, target lists are confined to relatively few priority pest species identified by previous risk assessment [9] or statistical methods are used to select only a subset of specimens for species level identification [29]. These restrictions can result in unanticipated or cryptic invasive species that are not being actively monitored for, to go undetected [30].

In order to overcome the limitations of current identification methods for processing large numbers of specimens, recent studies have looked to high-throughput sequencing (HTS) technologies to allow DNA barcode-based identification to be conducted in a massively-parallel manner. This process, termed "metabarcoding" [31] or "marker gene sequencing" [32], generates a large number of individual barcode sequences in a single reaction, enabling the simultaneous identification of individuals in large mixed communities [33,34], such as a trap sample containing many different insect species. The ability to rapidly and cost effectively survey biodiversity has led to metabarcoding being taken up across numerous fields of applied ecology [34–37], including the identification of invasive species (Fig 1A) [33,38–40]. By identifying both endemic

and potential exotic species in a bulk DNA analysis approach, metabarcoding can remove the time-consuming specimen sorting required by previous molecular and morphological diagnostic methods, and allow detection of not just key pests but also other unanticipated species that are not being actively searched for [38,41,42]. This aspect is particularly advantages for the detection of environmental threats, as when considering impacts beyond just agriculture and the time lag that can occur between introduction of a new species and perceptible damage to the environment [43], there are far more invasive species of threat than can be identified by risk assessment and incorporated into target lists [23,44]. A further advantage arises from the ability of HTS to count occurrences of specific sequences in a mixed sample [45] potentially allowing simultaneous pest identification and population size estimation. Finally, the rapidly increasing output of HTS technologies enables multiplexing of hundreds of trap samples in a single sequencing run, providing an avenue to dramatically scale up insect surveillance to the level required for effective, affordable and proactive management response.

Despite the advantages that metabarcoding may offer to insect surveillance programs, uptake of new diagnostic tools into operational use depends on more than just the cost-effectiveness of the tool, but also on factors such as ease of use, accuracy, reproducibility, perceived usefulness to the end users as well as compatibility with existing policy frameworks [46,47]. With the introduction of the World Trade Organisation Agreement on the Application of Sanitary and Phytosanitary measures (SPS) came new obligations for exporting nations to demonstrate freedom of a geographic area from particular pests using scientifically rigorous surveillance practices [48]. This agreement has in turn led to harmonisation of routine diagnostic procedures into internationally standardised protocols to ensure that all end users are aware of the particulars involved and therefore committed to accepting any risk management actions that arise through its use [46,49]. The SPS agreement recognises the International Plant Protection Convention (IPPC) and the World Organisation of Animal Health (OIE) as the internationally recognised standard setting

bodies for plant and animal health respectively [48], and adoption of new standards stems from exhaustive workgroup efforts by these agencies [13,50]. While the opportunities that HTS approaches could offer has been widely recognised by the diagnostics community [51,52], due to the relative infancy of the technology standards and guidelines around their use is a rapidly evolving space and validated protocols do not yet exist. Despite this, there is flexibility within the SPS framework for trading partners to introduce novel sanitary or surveillance procedures if it can be demonstrated that they are equivalent or better to previous methods [49] and both the IPPC and OIE have now released guidelines for those labs preparing to implement HTS approaches in routine diagnostics applications. These guidelines highlight the need for robust experimental designs, assay validation, and quality assurance [51,53,54], reflecting recent discussions in the wider metabarcoding community [55]. In this review we explore the application of metabarcoding for high-throughput species level identification of insects, providing an overview of common metabarcoding workflows (Fig 2) and considerations required at each step to ensure reliable detection and quantification of taxa within complex mixed communities. We further discuss the unique technical and regulatory challenges of integrating broad-spectrum HTS assays into a diagnostic framework and offer a perspective on the future adoption of high-throughput insect surveillance within international biosecurity frameworks.

REVIEW

Selecting a taxonomic marker

Appropriate selection of a taxonomic marker or barcode locus is a critical first step in design of a metabarcoding assay, as all downstream species detection and identification will rely on how conserved this marker is across taxa, and the discriminatory power of the nucleotide variation contained within it [56]. The markers most commonly employed in metabarcoding studies are those already widely adopted for conventional DNA barcoding, and therefore the mitochondrial

cytochrome oxidase I (COI) locus has been the most widely used marker for metabarcoding of insects to date. The 658bp region of COI [57] used for conventional DNA barcoding has a strong track record of delivering species level identification of insect pests [58], however many HTS platforms impose strict limitations in molecule length that can be sequenced (Table 2) and therefore smaller stretches of the conventional barcode loci or 'mini-barcodes' must be used [59]. Nevertheless, research into degraded DNA samples has shown that singular COI barcode of sizes between 135bp [60] and 250bp [61] can reliably distinguish most animal species, however appropriate placement within the larger barcode region is essential [62]. Despite the excellent taxonomic resolution provided by COI, since its application to metabarcoding a number of further limitations have become particularly apparent. As COI is a protein coding gene, the third position of codons can be variable, leaving no strictly conserved nucleotide sites for design of universal PCR primers [63]. This mismatch inevitably leads to primers having variable affinity for different template molecules, biasing the amplification towards well-matched taxa and failing to amplify others [64]. Unlike conventional DNA barcoding where a failed amplification will result in a noticeably absent PCR product, in a bulk sample failed amplification of a particular taxon will be masked by the recovery of sequences from other taxa and therefore will go unnoticed [63]. A further issue inherent to mitochondrial loci such as COI is the proliferation of nuclear mitochondrial pseudogenes (numts) in many insect orders [65–67], the result of historical recombination between the mitochondrial and nuclear genomes [68]. Co-amplification or preferential amplification of these pseudogenes instead of the true mitochondrial locus can complicate species identification [67] and result in overestimation of taxonomic diversity in the sample [69].

Due to the aforementioned issues, as well as the inability for COI to differentiate certain pest groups [70], a range of alternative universal barcode markers have been proposed (reviewed by Freeland [56]). Ribosomal RNA (rRNA) genes are particularly appealing due to their high copy

number and stem-loop structure that consists of highly conserved core sequences for primer binding, interspaced with variable regions providing taxonomic resolution [71,72]. Despite this, rRNA regions are on average more conserved than COI and therefore while appropriate for reconstructing higher level relationships they require longer spans of nucleotides to be informative at the species level. For single specimen barcoding this can be overcome by concatenating several markers to increase phylogenetic resolution [73], however this presents a challenge for metabarcoding of mixed communities as there no way of knowing whether two non-overlapping markers are from the same individual [74]. Therefore, while multi-locus approaches can be useful for expanding the taxonomic diversity an assay can recover [75–77], in particular cross kingdom diversity (Box 2), they do not necessarily provide greater resolution [45]. Consequently, closely related and difficult to diagnose pest taxa may require further studies to identify appropriate diagnostic loci [78], or the development of novel analytical methods to integrate taxonomic assignments from multiple independent barcode loci. Finally, the application of alternative markers to insect diagnostics will suffer from a lack of reference sequence data, as many taxa, including those of economic importance currently only have COI sequence data publicly available (Fig 3B, 3C). Therefore, as species level resolution is a requirement of many diagnostic standards [24,49,79], for the taxa in which it has sufficient resolution, the high mutation rate and extensive reference information obtainable for COI will maximise the utility of metabarcoding within a broad-spectrum surveillance programme [80].

Box 1 – Reference sequence databases

As with conventional DNA barcoding, accurate taxonomic assignment in metabarcoding studies relies on a well-curated reference database of DNA marker sequences tied to vouchered morphological specimens to compare query sequences against [81]. The primary public nucleotide databases of relevance to insect metabarcoding are the Barcode of Life Data System (BOLD) [82] and the NCBI GenBank database [83]. While GenBank hosts greater overall

sequence data, BOLD represents a curated DNA barcoding database that aims to maintain consistent links between sequences, validated morphological specimens, and associated specimen collection metadata [84]. Concerted efforts to generate mitochondrial COI barcodes for major insect orders have led to broad coverage of insects of biosecurity concern in both major public databases [58], however many geographic regions are still under sampled (Fig 3A) and reference sequences for alternative loci are mostly unavailable (Fig 3B, 3C). While continued public submission and high throughput reference sequence generation [85] will increase the representation of missing taxa and loci over time, ensuring the quality of submitted sequences from correctly identified specimens is crucial [24]. There are numerous examples of barcode sequences being either insufficiently annotated [34], annotated with the incorrect species in public databases [81,86–89], or multiple morpho-species assigned to the same DNA barcode, which may reflect misidentifications or the existence of species complexes [58]. These issues highlight the importance of engaging taxonomic experts to ensure *a priori* identification of a specimen before submitting a reference barcode to a public database [90,91]. Furthermore, the use of non-destructive DNA extraction methods when generating barcode sequences would allow the retention of voucher specimens to ensure traceability between the molecular and morphological features, especially in the case of taxonomic reassignments [92].

While some metabarcoding studies have responded to the aforementioned issues by exclusively using in-house reference databases for taxonomic assignment [90,93–95], as many insect surveillance programmes aim to detect species that are not locally present, the reliance on public data to supplement in-house sequences may be unavoidable. Some taxonomic classifiers used in metabarcoding studies provide the option to weight classifications towards certain reference sequences [96,97], which could be beneficial when combining high confidence in-house sequences with more variable quality public sequences, or when the endemic diversity for the target region is well characterized [74,98]. Regardless of source, barcode sequences will be

compiled together and formatted appropriately for use with automatic taxonomic classification software [99–101] and this presents an ideal point where automated or semiautomated curation methods can be used in order to identify and remove any taxonomically mislabelled sequences or non-homologous regions such as pseudogenes [74,102]. Finally, curated databases used in an active surveillance program should only be updated after rigorous testing with standardized datasets to ensure assay results remain accurate and reproducible following addition of new sequences [103].

Marker enrichment

Similar to conventional DNA barcoding, most metabarcoding studies use a set of universal oligonucleotide primers to exponentially amplify a target barcode marker until it reaches a concentration appropriate for sequencing. This 'amplicon sequencing' methodology has proven reliable and sensitive for detection of low abundance taxa in bulk samples [40]. However differential PCR amplification efficiencies between taxa generally results in a biased depiction of relative abundances of community members [104]. This bias is thought to mainly arise from primer-template mismatches, particularly at the 3' end of the primer where extension takes place [64,105] and therefore comprehensive *in-silico* evaluation should be conducted at the beginning of a project to ensure primer sequences are appropriate for the underlying target community [106–108]. Where mismatches with certain taxa are predicted to occur, inclusion of degenerate bases can overcome taxonomic bias inherent to a specific primer sequence [109,110], however high levels of degeneracy can also lead to undesirable off-target amplification or formation of dimers [87,111], which will require further laboratory validation to detect [71,109,112]. In addition to the effects of PCR primers, a range of template specific factors including copy number of the loci [113], nucleotide composition and secondary structure [114], variable amplicon lengths [115], specimen biomass [116], and complexity of the species mixture [105,117] can further contribute bias. While the cumulative bias from all these factors may suggest that amplicon sequencing can

only be used for presence-absence data, importantly, sequencing reads are still correlated with DNA input in a predictable way, and biases should only affect the slope of that correlation [113]. Therefore the calculation of taxon-specific correction factors shows great promise for improving abundance estimates from metabarcoding data [113,118–120], particularly for simpler communities such as those trapped using targeted attractant lures [17]. Nevertheless, if accurate quantification is essential for the surveillance programme, removing the PCR amplification process altogether should also be considered for improving taxon abundance estimates from metabarcoding data.

PCR-free approaches

The major alternative to amplicon sequencing based metabarcoding involves simply fragmenting the genomic DNA extract to lengths appropriate for the sequencing platform and directly sequencing it without any prior bias-inducing enrichment step. This methodology, termed 'shotgun metagenomics', generates sequence reads comprising a random subsample of the mixed community DNA and relies on the higher representation of taxonomically informative multi-copy mitochondria and nuclear rRNA in this subsample to identify community members [121–123]. In addition, these high copy regions can be assembled into long contigs and even full length mitochondrial genomes for further phylogenetic inference and systematics applications [124,125]. Despite this, restricting taxonomic analysis to just mitochondrial and nuclear rRNA regions still leaves the vast majority of reads corresponding to DNA that is not taxonomically informative or easily assembled from a bulk sample to be discarded [121] and deep sequencing will be required to reliably detect rare specimens in the community [125,126]. While the rapid growth in sequencing capabilities is making this brute force approach to community identification increasingly possible, for routine surveillance a cost-effective method for enriching taxonomically informative loci should be used prior to sequencing. A range of potential methods for PCR free sequence enrichment have been reviewed elsewhere (see: Mamanova et al [127] and

Jones et al [128]), but some examples that have been successfully used for metabarcoding include differential centrifugation to enrich for mitochondria [129] or baiting target barcode markers and whole mitochondria using hybridisation probe capture [130–133]. Hybridisation capture relies on the use of thousands of synthetic oligonucleotide probes each with strict complementarity to a target sequence, and therefore should ideally be designed with *a priori* knowledge of every target sequence [128]. Although this may be a limiting factor for recovery of previously unsequenced diversity, the flexibility to include essentially infinite numbers of probes provides further advantages for building bespoke metabarcoding assays that capture diverse loci for purposes beyond taxonomic inference (Box 2). Nevertheless, while PCR-free approaches have shown improved correlations between sequencing reads and input DNA [123,134], it is important to remember that HTS counts molecules not individuals [45] and therefore biases are likely to still remain due to variation in biomass and copy number between organisms and tissues [131,134]. Furthermore, the process of PCR amplification is already widely accepted with validated diagnostics protocols [49], and implementation of alternative PCR-free sequence enrichment methods may require overcoming additional regulatory hurdles.

Box 2 – Modular metabarcoding assays

Many of the insect pests actively monitored by surveillance programs are not targeted because of direct damage they do to animals, plants or the environment, but instead due to the associated fungi, bacteria, viruses and viroids that they can vector [52,135,136]. Similar to identification of insects, detection of host-associated pathogens has previously required screening of trapped samples on a specimen-by-specimen basis using target-specific assays or culturing and morphological analysis [33], however this is rapidly being augmented with metabarcoding and metagenomic approaches [33,103,137,138]. The ability of HTS platforms to sequence a heterogenous mix of loci opens up the opportunity for combining both the identification of insects and the screening of a diverse range of host-associated microbiota within a single

multiplexed metabarcoding assay [40,139]. Nonetheless, developing an integrated assay that allows detection and identification of biologically diverse organisms in a diagnostics context presents a number of challenges. Extraction techniques will need to be optimised account for the pathogen association with its insect host (i.e. intracellular [140], external [141], gut borne [142]) and specific microbial life histories may make this incompatible with non-destructive DNA extraction. Furthermore, PCR protocols will need to be optimised to account for the large differences in template quantity between abundant host DNA and low-titre vectored organisms [143].

In contrast with the high resolution COI provides for identification of insects, the commonly used universal markers for bacterial and fungal barcoding struggle to identify organisms to the species or strain level, which is necessary to separate pathovars from common innocuous environmental organisms [33,136]. Therefore, diagnostic assays that aim to be universal for both host and vectored organisms identification will require analysis of a range of group-specific markers in multiplex, or make use of long read HTS platforms for increased taxonomic resolution [144,145]. While multiplexing many loci together in single PCR reactions can greatly simplify laboratory protocols and therefore costs involved, for metabarcoding this can be complicated by cross-reactivity between primers and individual primer sensitivities changing depending on community composition [76,105,112]. As an alternative, various target loci could be enriched in parallel reactions and then pooled together by sample prior to library preparation In proportions relative to the number of reads desired for each marker [40,146]. This highly flexible modular approach would then allow group-specific microbial primers, or other markers of interest to be added or retracted from the assay depending on the target community and needs of the end user. For example, Swift et al [147] have demonstrated the ability of modular metabarcoding assays to not just identify cross-kingdom species composition, but also genotype microsatellite loci and sex specific markers relevant to the community under study. While the

field of invasion biology has traditionally been concerned with the transport and movement of species, this doctrine overlooks the intra-specific movement of genetic material such as pesticide resistance alleles [148], transposable elements [149], and genetically modified organisms [150]. The ability to capture essentially any loci in a modular metabarcoding assay may allow integration with a more gene focused model of biosecurity in the future.

Library preparation & multiplexing

Regardless of whether an enrichment or metagenomics approach was used, platform specific sequencing adapters need to be attached to the molecules (via ligation [151], one-step [152] or two-step PCR [40,106]) to form 'libraries' which can then bind to the flow cell for sequencing (Fig 4A). As current HTS platforms output sequences far in excess of what is required to identify the taxa in a single community, metabarcoding studies commonly multiplex many samples together on a single flow cell and use oligonucleotide index sequences incorporated into the sequencing adapters to link sequencing reads back to origin sample. While a range of indexing strategies exist for HTS [153], for sensitive diagnostics applications it is critical to choose an approach that can adequately cope with the occasional recombination of these indices between molecules. Index-switching has received particular attention due to the particularly high levels on recent Illumina platforms [154], however similar phenomena can affect multiplexed sequencing across major platforms to various degrees [155–159] (with the possible exception of recent MGI platforms [160]). Suggested causes include contamination from residual adapter/primer oligonucleotides [161], chimera formation during adapter PCR [162], mixed clusters on the flow cell [157], or physical contamination during library preparation or oligo synthesis by the vendor [159,163,164]. Regardless of mechanism, when not properly controlled for, index-switching can cause taxa from one sample to 'bleed' into others, and while this will only produce false-positives for a taxon of concern when a true positive is present in at least one of the samples, the spreading of positive signal across samples can imply the taxa of interest has a larger geographic

distribution than reality. Recent studies have demonstrated the most effective method for controlling for index-switching is through the use of unique dual indices (Fig 4C) rather than the commonly used combinatorial indexing (Fig 4B). When unique dual indices are used, switching events at either end of the molecule will generate an index combination that was not originally applied and during de-multiplexing the reads with mismatched indices to the sample sheet will be filtered into an unassigned reads file and excluded from analysis [159,162,165]. Furthermore, sets of indices should be alternated for each sequencing run [51] as carryover of molecules between runs on a HTS machine can be a further cause of false-positives in high sensitivity sequencing applications [166]. Finally, it is further important that index sequences used are designed with sufficient edit distance between them so that substitution or insertion/deletion errors within the index do not cause further sequence mis-assignment [131,167], particularly for higher error rate platforms such as nanopore [115].

High-throughput sequencing platforms

While the rapid growth of HTS over the past decade has produced a variety of techniques and chemistries for discerning the nucleotide sequence of a DNA molecule [168], modern platforms can largely be divided into those producing short-but-accurate sequences, or long-but-error-prone sequences (Table 2). To date the majority of metabarcoding studies have been conducted using the former, with the Illumina 'MiSeq' dominating the recent metabarcoding literature due to its high-quality reads and relatively inexpensive purchase cost (Fig 1B). Despite the current popularity of the MiSeq for research studies, the cost per sample may be impractical for the number of specimens produced by large-scale surveillance programmes, and instead the production scale Illumina 'NextSeq', 'HiSeq' and 'NovaSeq' provide progressive increases in throughput and therefore cost reductions (Table 2). Nevertheless this increased sequencing throughput of these platforms must be balanced with diagnostic turnaround times, and effective use of the ultra-high capacity HiSeq and NovaSeq flow cells will involve multiplexing of

thousands of samples, requiring significant logistical efforts in sample collection and processing [103].

Despite the cost-effectiveness of the aforementioned platforms, their restricted read lengths (Table 2) limit the taxonomic resolution achievable with a metabarcoding assay and therefore long read sequencing platforms such as the Pacific Biosciences (PacBio) 'Sequel' and Oxford Nanopore Technologies (ONT) 'MinION' and 'PromethION' are becoming increasingly attractive alternatives. The ability to sequence barcode regions thousands of bases in length has potential to enable greater recovery of taxonomic diversity with intra-specific resolution [169], however in practice the utility of long reads for species identification has been limited by significantly higher per-base error rates that commonly exceed intraspecific distance [115,170]. Nevertheless, methods for repeatedly sequencing a single molecule to create higher quality consensus sequences [171] are now opening up applications in metabarcoding [144,158], with natively implemented circular consensus sequencing on the PacBio Sequel producing consensus reads with similar accuracy to traditional Sanger sequencing [172], and third party protocols mimicking this approach have now been published for the ONT platforms [173,174]. If similarly robust consensus sequencing can be achieved with nanopore technology, the significantly smaller start-up cost and portability of the handheld MinION platform may in future permit metabarcoding based diagnostics to be conducted in remote field sites [115], as well as enable lesser resourced laboratories to access these technologies [14].

Bioinformatics

Computational processing of sequence reads represents a series of steps of equal importance to laboratory protocols for ensuring accurate and sensitive detection of invasive species [175,176], however many of the skills and techniques involved in this process have not historically been required within diagnostic laboratories. While there exists a number of popular end-to-end

computational pipelines for analysing marker gene data [177–181], many of these have been designed for measuring diversity rather than detection of low abundance taxa. Each step in the bioinformatic analysis can present trade-offs between sensitivity to rare taxa, amount of erroneous sequences retained, and overall computing time [77,175,182–184] and use of metabarcoding in an invasive species surveillance or other sensitive context presents some unique challenges and regulatory requirements that may be best addressed through the creation of a custom analysis pipeline [146,176].

De-multiplexing and sequence quality trimming

A metabarcoding assay typically involves multiplexing many samples into a single pooled sequencing library in order to make optimal use of the high capacity flow cells of current sequencing platforms. Therefore, the first step following sequencing (typically automated by the HTS platform's software) is to assign sequences back to their origin sample using unique oligonucleotide sample indices incorporated into the sequencing adapters (Fig 4). Following de-multiplexing, sequencing adapters and any other non-biological information such as PCR primer sequences are removed, and reads are assembled into consensus sequences using their overlapping bases. While improvements in underlying sequencing chemistries and aforementioned consensus approaches means the majority of platforms now provide per base accuracies above 99.99% (with the notable exception of nanopore platforms) [168,173,185], when put in context of the billions of bases sequenced on modern flow cells, tens of thousands of sequences will still contain errors [186]. Raw sequence reads are generated in conjunction with a predicted error profile based off signal intensity and background noise, and this data is generally presented to the user in the form of a FASTQ file [187]. An initial quality trimming stage uses this profile to truncate or remove sequences that contain excessive ambiguous or low confidence base calls [186,188], this is however a coarse filtering process where parameters should be carefully considered, particularly for higher error platforms such as nanopore. While

strict quality trimming will more effectively remove sequencing artefacts and erroneous reads that can impact downstream diversity and abundance estimates, overly conservative parameters can result in removal of too many reads and therefore loss of sensitivity to low abundance taxa [146,176].

OTU clustering & denoising

While quality trimming can improve accuracy by removing sequencing errors, the PCR amplification process used in the majority of metabarcoding studies can further introduce single base substitutions [158,189] and length variation [190] that will not necessarily be associated with low quality scores [191]. As these noisy sequences can cause spurious results and significantly increase downstream computation, many studies cluster together all sequences within an arbitrary similarity threshold (commonly 97%) into representative bins called 'operational taxonomic units' (OTUs). While the 97% similarity threshold is thought to represent a broadly generalisable compromise between interspecific and intraspecific variation and is commonly used to indicate distinct taxa [192,193], actual coalescent depths between species can differ greatly across taxonomic groups [91]. Therefore when a single global threshold is applied to diverse communities it can result in both the splitting of a single species across multiple OTUs, as well as the lumping of multiple species into the same OTU, resulting in false-negatives [176,194]. Furthermore, aggregating all similar sequences into a single OTU loses all information on intraspecific diversity, restricting the ability to trace geographic origin of invasive populations [39,79]. In addition, the OTUs generated by clustering are dependent on the particular dataset, reference database, and parameters selected [194,195], and as such they do not lend themselves to ongoing comparison with the constantly evolving data produced by a longitudinal surveillance programme. In order to overcome the aforementioned limitations, newly developed 'de-noising' algorithms instead use statistical models to infer true biological sequences from sequencing noise and correct for single nucleotide differences, without imposing the arbitrary similarity threshold

which define OTUs [196–198]. This single nucleotide resolution enables binning sequences into 'amplicon sequence variants' (ASV) [196] (also termed 'exact sequence variants' (ESVs) [194], sub-OTUs [197] or zero-radius OTUs (zOTUs) [198]) that retain precise haplotype information that can be necessary for diagnostics of closely related taxa or tracking an invasion [199], and act as a consistent label between analyses [194].

OTU quality control

While the above measures account for the majority of low abundance errors, they are not designed to deal with high abundance artefacts such as PCR generated chimeras and non-specific amplification products. Chimeric sequences are the result of incompletely extended PCR products acting as primers for a different closely related sequence [189], and therefore appear as concatenated products of two parent sequences. Assuming parent sequences will be more abundant having undergone more rounds of amplification, chimeras can be algorithmically removed through comparison with other sequences in the sample [196,200], or with a chimera-free reference database [201]. On the other hand, removing products of non-specific amplification such as intra-genomic variants and pseudogenes presents more of a challenge, and will generally require manual curation [151,202]. When targeting protein coding mitochondrial genes such as COI, the presence of stop codons and frameshifts that disrupt the open reading frame (ORF) are common indicators of pseudogenes [80], and for rRNA markers secondary structure prediction could be used to ensure sequences don't contain significant variation in highly conserved regions [203]. As it is inefficient to include a manual curation process as part of a high-throughput bioinformatics pipeline, it would be beneficial for future denoising algorithms to incorporate patterns of sequence evolution to allow more precise and automated filtering of barcode loci from erroneous and pseudogenic sequences [80,204,205].

Taxonomic assignment

In order to process the large diversity of sequences that a metabarcoding assay typically produces, the assignment of Linnaean taxonomy (species, genus etc.) is typically conducted in an automated manner. While a large range of software tools exist for this purpose [206], the approaches used can generally be delineated into either sequence similarity searches (i.e. BLAST alignment), sequence composition methods (i.e. Hidden Markov models and Kmer counts), phylogenetic methods, or a hybrid of the above (see Bazinet et al [207] for an in-depth comparison). To date, the most widely used approach for taxonomic classification in metabarcoding studies has been best-hit classification using alignment based tools such as BLAST [208], which assume that the taxonomy of the query sequence will be identical to the taxonomy of the most similar sequence in a reference database. While this approach is simple to implement and can perform effectively when the reference database contains sequence information from conspecifics, when reference data is absent or when the particular loci cannot distinguish between multiple organisms, best-hit classification is prone to over-classifying the sequence to incorrect species level taxonomy [209]. In the worst case, this over-classification error could lead to false-positives by classifying a previously un-sequenced but probably innocuous organism as a known pest, due to the pest being the closest taxa with an existing reference sequence [210].

As the above situation demonstrates, for applications where management decisions are to be based on the results of a taxonomic classification, a central question is the reliability of that classification. A number of taxonomic assignment algorithms aim to address this issue by returning a measure of confidence of inclusion in each taxonomic rank, for example using repeated random sampling [97,211], lowest common ancestor methods [212], or probabilistic models [96,213]. In an ideal case, only a single possible taxonomic outcome will obtain a high level of confidence, whereas alternate outcomes will obtain probabilities close to zero. In cases where there may be uncertainty at the species or genus level due to imperfect reference data and

multiple taxonomic outcomes obtaining similar probabilities, the sequence may still be robustly assigned to a higher taxonomic rank (e.g. family) [101], providing important information about sample composition and possible presence of novel taxa without producing false-positives [214]. While employing measures of confidence can reduce incidence of over-classification, many of these approaches suffer from an inherent bias in that they infer the entire scope of possible taxonomic outcomes exclusively from the reference sequences used for training [215,216], which in reality only represents taxonomic units that have been previously sequenced. In contrast, the Bayesian framework of PROTAX [96] accepts a reference taxonomy tree alongside the reference sequence database in order to account for taxa that are present in Linnaean taxonomy but not represented by reference sequences. Furthermore, PROTAX explicitly models the probability that a sequence belongs to a taxon that is novel to both the reference sequence database and reference taxonomy, which could be particularly important when conducting surveillance in regions with significant uncharacterized biodiversity [216,217]. Nevertheless, even the most complex taxonomic assignment algorithms do not model important aspects of species biology that may limit the possible geographical distribution or habitat they could reasonably exist in, and therefore the results of taxonomic assignment should be vetted with ecological knowledge of the detected species where possible [35].

Quality assurance

The ability to simultaneously identify many loci from thousands of specimens in a single diagnostic assay underlies the power of the metabarcoding approach to surveillance, however the resulting increase in sequence diversity and analytical complexity introduces further risk of cross-contamination and technical error [55]. An important challenge for the use of metabarcoding in a diagnostic context is the rate of false-positives (incorrect identification of an insect as the pest of concern) and false-negatives (not identifying a pest of concern). While many ecological studies prioritize minimizing false-positives errors over false-negative errors [37], generally the

precautionary principle applies in biosecurity, i.e. it is better to have a false-positive that can be followed up with an orthologous confirmation method than to miss a serious pest. This is particularly important if the assay is to provide 'evidence of absence' to support pest free status [218], which can be required to access certain international markets [28]. Therefore, a quality assurance system for metabarcoding diagnostics should aim to reduce false-positives as much as possible through the appropriate use of controls, replication and validation, without in turn increasing the incidence of false-negatives.

Controls & replication

The majority of contamination in NGS assays is expected to arise from other samples processed in the same laboratory environment, particularly when PCR is involved [164,219], and therefore workspaces should be physically or temporally separated for different assay steps with all surfaces, equipment and reagents regularly decontaminated [33,219–221]. Periodic swipe tests of laboratory surfaces can help identify common laboratory contaminants and confirm the absence of environmental DNA from target pests [220,222]. Despite these precautions, even the cleanest laboratory environment will not account for all possible contaminant sequences and therefore no-template controls should be included throughout the entire laboratory workflow and sequenced alongside the sample libraries to provide a cumulative measure of contamination [162,223,224]. When these controls are incorporated sequentially at each step of the laboratory protocol they can further enable partitioning of contamination to the stage in the workflow where it occurred, which can be particularly for highlighting processes that can be improved during assay development [35,37]. Index-switching is perhaps the most worrisome cause of contaminating sequences in HTS, and while use of unique-dual indices (Fig 4C) can reduce this phenomenon to a level acceptable for most studies, trace levels of index-switching can still persist and cause issues for sensitive diagnostic applications [159]. While index-switching artefacts will be detectable in no-template controls, it can be difficult to discern this

phenomenon from sequences arising through physical contamination. Instead, including a positive control library made up of synthetic standard DNA [177,225,226] or an 'alien' taxa guaranteed to be absent from the sample [88,227] allows empirical measurement of the index-switch rate. Alternatively, the rate of index-switching can be measured post-hoc by comparing read counts between valid and invalid combinations of unique-dual indices [131,228]. Once contaminant sequences have been identified, their presence can be controlled through the application of a minimum abundance filter based on the read counts within negative and/or positive control libraries [35,229], although choice of an appropriate threshold can be complicated by read depth differences between samples and preferential amplification of contaminants in low biomass no-template control samples [175,230]. As an alternative, new statistical methods allow systematic removal of contaminant sequences based on co-occurrence patterns and library quantification data [231–233], however if particularly high levels of contamination or abnormally high rates of index-switching are detected in a specific batch of samples it may be more appropriate to repeat the assay. Finally, including an additional positive control in the form of a well characterized mock 'calibration community' in every sequencing run could further highlight any additional run specific aberrations or batch effects that may have been introduced during the metabarcoding workflow when taxonomic composition or error rates deviate strongly from expected [205,234,235].

In addition to being prone to contamination, library preparation protocols involve a series of molecular bottlenecks where during each subsequent stage of DNA extraction, target enrichment and binding of molecules onto the flow cell, only a random subsample of molecules are taken forward [37]. Stochasticity in this sampling process is likely to bias the resulting sequences towards more abundant taxa and increase the false-negative rate for rare taxa [236], and this can be further exacerbated by negative primer bias [77]. Potential loss of rare taxa during sample processing can be offset through the use of technical replicates, and these provide a further

avenue to identify laboratory cross-contamination in the case that replicates show significant dissimilarities in taxonomic composition [77,229,237]. While using higher numbers of replicates can increase the probability of detecting rare taxa [237], this must be weighed against the increased costs of sequencing and library replication as well as the strategy for processing the replicates [37]. Additive processing (i.e. pooling the detections of all replicates) can be most useful for overcoming sampling stochasticity and controlling for false-negatives, while restrictive processing (i.e. only retaining sequences present in several replicates) more effectively controls for cross-contamination. To balance the positives of both approaches, it may be best to include a minimum number of technical replicates to allow a majority rules approach (e.g. 2/3 replicates count as a detection) [77,88,112]. A further aspect to consider is the importance of biological replicates at the sample collection stage [238], as regardless of the effectiveness of the metabarcoding diagnostic assay if an insect is not caught in a trap it does not necessarily mean absence in the area. The use of site occupancy models that account for the false-positive and false-negative prone nature of metabarcoding surveys could be used to determine the optimal number of both technical and biological replicates to reach the desired statistical power for the survey [239,240]. Finally, while out of the scope of this review, appropriate trap design [241] and surveillance grid planning [242] must also be adhered to for effective metabarcoding based surveillance.

Validating metabarcoding assays

Due to the relevance of many invasive insects to international trade and human health, laboratories conducting insect diagnostics generally exist within strict regulatory environments. As part of laboratory accreditations, newly developed assays are required to undergo a validation process in order to provide objective evidence to all end users that an assay is fit for purpose [53,54,243,244]. Traditionally, validation first involves defining the scope of the assay and then establishing performance parameters such as analytical sensitivity, analytical specificity,

reproducibility and repeatability for every individual target designated in this scope [26,244,245]. However, the universal nature of metabarcoding assays and the taxonomic diversity of potential surveillance catch makes this impractical [246]. To overcome this inevitable variation between reference samples and reality, a flexible scope validation process should be used to establish performance parameters on representative samples and identify critical steps in the workflow where variation can be introduced [146,247]. These critical steps can then be monitored run-to-run using control samples and appropriate QC checkpoints (Table 3), in order to ensure that no sample or sequence data continues without meeting minimum quality requirements [51,221,247,248]. In the case of insect metabarcoding, mock communities made up of the taxonomic groups of interest are generally used for validation, which are then spiked with decreasing concentrations of target species in order to establish assay sensitivity and limits of detection [40,249]. As DNA extraction efficiency and primer bias can be affected by overall community complexity [105,250], mock communities should as closely as possible represent the diversity expected to be recovered in different trapping scenarios. Furthermore, the amount of sequencing effort assigned to an individual sample during multiplexed sequencing can vary across runs [224,251], and the effect of sequencing depth on detection should also be established using rarefaction curves [107,117]. On the other hand, analytical specificity will generally depend on choices made during assay design, such as the choice of target marker, availability of appropriately annotated reference sequences for the chosen marker, and taxonomic assignment criteria used [220,246]. Parameters such as precision and reproducibility of a metabarcoding assay can be established similar to other molecular diagnostics, through replication of samples and controls within and across sequencing runs and inter-laboratory comparisons [146]. Finally, stability of specimens and DNA to environmental factors such as temperature, UV radiation, pH of commonly used drowning or attractant solutions (e.g. vinegar traps [252]), and exposure to environmental microorganisms in the field and during storage [253] should be evaluated, and

may prompt a need for redesign of insect traps to collect and preserve samples in a manner more suited to DNA based identification.

Reporting & confirming detections

Even when primers are designed around a specific taxonomic group, metabarcoding can amplify and detect many more taxa outside the scope of the original validated target list [254]. How these incidental detections are reported and eventually acted upon will present a major challenge to diagnostic labs and end users, due to the increased number of previously undocumented taxa being discovered for which knowledge of distribution or ecological significance may be missing [51,53]. Many of these incidental detections will be taxa that simply have not previously been searched for, and it will be important when considering an appropriate management response not to conflate 'first detection' in an invasion biology sense, where there was prior evidence of absence, with merely the first time a species has been formally identified in a region [255]. Hence a greater emphasis needs to be placed on conducting baseline surveys to establish comprehensive species checklists of endemic diversity and resolve synonymous taxa at the beginning of a surveillance programme in order to avoid creating sudden market access and trade issues [256]. Furthermore, a decision framework should be developed for evaluating incidental detections that sets out steps for further characterization and risk assessment for the detected organisms in order to establish if eradication or other management actions are appropriate or achievable [257]. Where necessary, putative detections can be further confirmed using an orthogonal diagnostic method such as qPCR/ddPCR on the original DNA extract [146], however these assays require prior development and will therefore not be available for all incidental taxa detected in a metabarcoding assay. Instead, the use of non-destructive DNA extraction methods that use a combination of enzymes, buffers and heat without mechanical homogenisation [227,258–260], or even amplification of insect DNA from the ethanol used to preserve specimens [261–264] would enable diagnosticians to revisit original samples following metabarcoding to confirm

species detections. Development of a non-destructive metabarcoding assay has great potential for bridging the gap between new HTS methods and traditional entomological techniques and may bootstrap the acceptance of metabarcoding into international regulatory frameworks.

Perspectives & conclusions

The ability to accurately, rapidly and cost-effectively determine the species composition of bulk insect traps using metabarcoding has the potential to revolutionise broad-spectrum surveillance for invasive insect pests. Similar to any novel technology, as metabarcoding transitions from purely research to management applications it faces the growing pains that come with integration into established regulatory structures. While rigorous standardisation of both laboratory techniques and data analysis has proven essential for the acceptance of conventional DNA barcoding as a validated diagnostic for insects of regulatory concern [26,79], the sheer pace of development of HTS technologies and platforms may complicate similar standardisation of metabarcoding protocols. Historically, the effective lifespan of many HTS platforms has only amounted to a few years before obsolescence [168], and laboratory protocols and bioinformatic methods are therefore constantly evolving to chase this moving target. In response to this constantly shifting state of the art, harmonisation efforts by regulatory bodies should avoid the over prescription of restrictive standards into law, as these will become quickly outdated and risk further widening the gap between research and diagnostics capabilities [46]. Instead, development and distribution of certified reference materials in the form of standard and diverse mock communities or DNA standards (similar to the ZymoBIOMICS microbial mock community standards [265]) as well as computational datasets [266] would enable benchmarking of laboratory and computational methods and begin to characterise the sources of technical variation between laboratories [267,268]. This could be further developed into an inter-laboratory proficiency testing program where blinded reference samples are periodically distributed for analysis, in order to demonstrate to all stakeholders that an assay is fit for purpose

for detecting invasive insect species [248,269]. The results of these processes would allow further development of best-practice technical guidelines and begin to harmonise approaches across the wider metabarcoding community [270].

Biosecurity and pest management decision making is still largely reliant on the application of a species name to a specimen barcode sequence [81], and issues of mislabelled sequences in public reference databases (Box 1) highlight the importance of maintaining expertise in taxonomy and classical diagnostics to complement high-throughput approaches. Due to the incomplete nature of reference databases, much of the sequence data currently produced by metabarcoding assays will consist of insufficiently identified sequences [84]. While some of these will no doubt be the result of sequencing errors making it through quality control, many more will represent real taxa and reflect the further work required to more completely describe and acquire reference data for insect biodiversity. Monitoring programs for biological invasions are at their most informative when they are continuous and long term [271,272] and it would be beneficial for these insufficiently identified sequences to be integrated into references databases and tracked across analyses and timepoints. Porter and Hajibabaei [84] have highlighted the advantages that ASVs provide over more traditional OTU methods for consistent labelling of insufficiently identified sequences, and embracing non-destructive DNA extraction techniques would further enable taxonomists to verify these sequences using morphological methods and potentially locate previously unbarcoded taxa or novel species, which could then feed back into reference databases [259]. Conventional DNA barcoding and morphological taxonomy currently benefit from a close and reciprocal interaction [273], and we envision a similar relationship for the future of insect metabarcoding. This ability to systematically reanalyse historical datasets with improved reference databases, bioinformatic tools, and biological knowledge presents a major strength of HTS diagnostics [51] and therefore raw datasets should also be archived alongside relevant technical and environmental metadata in a machine readable format [195]. However the datasets

from ongoing longitudinal surveillance quickly amount to terabytes of data [274], the storage, management and securing of which will require dedicated infrastructure and personnel [53]. Unlike the current drive for open sharing of data in academic research, concerns of misuse harming the international movement of goods means that historically the release of raw diagnostic data to the public has not been common [51]. However, a pathway for declassifying and releasing this data to researchers should be developed, as the mass of community level information generated by metabarcoding bio-surveillance shows great potential for generating new insights into the process and impacts of biological invasion [275].

In an increasingly globalised world, more effective and scalable utilisation of surveillance effort will be required to manage the spread and establishment of invasive species. While broad-spectrum approaches to surveillance have historically been limited by the overwhelming amount of diagnostics work generated, metabarcoding based diagnostics fundamentally change this dynamic by allowing entire communities of diverse organisms containing target pests, endemic species, and unexpected invaders to be simultaneously identified [41]. While present costs of technological investments may currently limit the uptake of HTS tools to only well-funded core diagnostic labs, we expect developments in portable real time sequencing will further enhance the availability of these tools to a much wider user-base worldwide. Furthermore, it is conceivable that the ongoing miniaturisation of sequencers may synergise with advances in microfluidic and lab-on-a-chip technologies [276] to produce a new generation of metabarcoding based "smart-traps" for remote monitoring [277,278]. Nevertheless, metabarcoding forms just a single component of a larger biosecurity toolbox that contains not only fast, cost effective and reliable means of diagnostics, but also predictive models, improved risk forecasting, field tested tools, and an overarching decision support system [46,52,135,137]. The future of biosecurity surveillance and pest management is a distinctly interdisciplinary area, and we encourage future research to involve closer collaboration between academic scientists, diagnosticians and the end

users that rely on effective surveillance data to manage the spread of invasive pests and pathogens.

Methods:

All articles containing "Metabarcoding" in their abstract, title or keywords were retrieved from the Scopus, PubMed and Crossref citation databases on 2019-06-20 using the rscopus [279], rentrez [280], and fulltext [281] packages in R 3.5.3 [282]. Duplicated article entries were detected using fuzzy string matching functions from tidystringdist [283], and filtered out using dplyr [284]. All articles containing keywords in their title or abstract indicative of invasive species or sequencing platform used (See supplementary 1 for full list of keywords) were then represented graphically by year of publication using ggplot2 [285]. A list of global insect pests was then retrieved from Ashfaq et al [58] and combined with additional pests of concern for Australia [286]. This list was filtered to retain only unique and complete genus species binomials, retaining 558 species, for which all records for these species and the entire Insecta were retrieved from BOLD using the bold package [287]. The list of genes successfully retrieved from BOLD used to query GenBank and all records for species on the pest list and the entire Insecta were retrieved using the Rentrez R package [280]. Records from all databases were combined and specimen collection information was extracted using R and the biofiles package [288]. Of the 5,589,069 records for all loci in the datasets, 4,603,488 were annotated with latitude and longitude information and these were plotted on a world map using ggmap [289]. The number of overall records and unique species within all datasets were then plotted for the top 10 occurring loci.

Acknowledgements:

We would like to thank the reviewers for comments and suggestions that greatly improved this manuscript.

ABBREVIATIONS

BOLD: Barcode of Life Data System; COI: cytochrome oxidase I; ESVs: exact sequence variants; HTS: High Throughput Sequencing;; OUT: Operational Taxonomic Units; zOTUs: zero-radius Operational Taxonomic Units

DECLARATIONS:

Ethics approval and consent to participate

Not applicable

Consent for publication

Not applicable

Availability of data and materials

A snapshot of the datasets and R markdown documents implementing the analyses contained in this manuscript are available in the Zenodo repository [290].

Competing interests

The authors declare that they have no competing interests.

Funding

This work was supported by funding from the Plant Biosecurity Cooperative Research Centre (PBCRC #2153), Horticulture Innovation Australia (ST16010), and Agriculture Victoria's Improved Market Access for Horticulture program (CMI105584). AP and JB were further supported by an Australian Government Research Training Program Scholarship.

Authors' contributions

AP and MB conceptualized the manuscript. AP drafted the manuscript with contributions from JB, JW, JPC, NC, BR and MB. All authors read and approved the final manuscript.

REFERENCES:

1. Hulme PE. Trade, transport and trouble: Managing invasive species pathways in an era of globalization. J Appl Ecol. 2009;46:10–8.

2. Meyerson LA, Mooney HA. Invasive alien species in an era of globalization. Front Ecol Environ. 2007;5:199–208.

3. Chown SL, Hodgins KA, Griffin PC, Oakeshott JG, Byrne M, Hoffmann AA. Biological invasions, climate change and genomics. Evol Appl. 2015;8:23–46.

4. Seebens H, Blackburn TM, Dyer EE, Genovesi P, Hulme PE, Jeschke JM, et al. Global rise in emerging alien species results from increased accessibility of new source pools. Proc Natl Acad Sci. 2018;115:E2264–73.

5. Paini DR, Sheppard AW, Cook DC, De Barro PJ, Worner SP, Thomas MB. Global threat to agriculture from invasive species. Proc Natl Acad Sci. 2016;113:7575–9.

6. Kenis M, Auger-Rozenberg MA, Roques A, Timms L, Péré C, Cock MJW, et al. Ecological effects of invasive alien insects. Biol Invasions. 2009;11:21–45.

7. Mazza G, Tricarico E, Genovesi P, Gherardi F. Biological invaders are threats to human health: An overview. Ethol. Ecol. Evol. 2014. p. 112–29.

8. Bradshaw CJA, Leroy B, Bellard C, Roiz D, Albert C, Fournier A, et al. Massive yet grossly underestimated global costs of invasive insects. Nat Commun. 2016;7:12986.

9. Andersen MC, Adams H, Hope B, Powell M. Risk Assessment for Invasive Species. Risk Anal. 2004;24:787–93.

10. Simberloff D, Martin JL, Genovesi P, Maris V, Wardle DA, Aronson J, et al. Impacts of biological invasions: What's what and the way forward. Trends Ecol Evol. 2013;28:58–66.

11. Lodge DM, Simonin PW, Burgiel SW, Keller RP, Bossenbroek JM, Jerde CL, et al. Risk Analysis and Bioeconomics of Invasive Species to Inform Policy and Management. Annu Rev Environ Resour. 2016;41:453–88.

12. Martin RR, Constable F, Tzanetakis IE. Quarantine Regulations and the Impact of Modern Detection Methods. Annu Rev Phytopathol. 2016;54:189–205.

13. Schrader G, Unger JG. Plant quarantine as a measure against invasive alien species: The framework of the International Plant Protection Convention and the plant health regulations in the European Union. Biol Invasions. 2003;5:357–64.

14. Early R, Bradley BA, Dukes JS, Lawler JJ, Olden JD, Blumenthal DM, et al. Global threats from invasive alien species in the twenty-first century and national response capacities. Nat Commun. 2016;7:12485.

15. Work TT, McCullough DG, Cavey JF, Komsa R. Arrival rate of nonindigenous insect species into the United States through foreign trade. Biol Invasions. 2005;7:323–32.

16. Joe Moffitt L, Stranlund JK, Osteen CD. Robust detection protocols for uncertain introductions of invasive species. J Environ Manage. 2008;89:293–9.

17. Liebhold AM, Berec L, Brockerhoff EG, Epanchin-Niell RS, Hastings A, Herms DA, et al. Eradication of Invading Insect Populations: From Concepts to Applications. Annu Rev Entomol. 2016;61:335–52.

18. Trebitz AS, Hoffman JC, Darling JA, Pilgrim EM, Kelly JR, Brown EA, et al. Early detection monitoring for aquatic non-indigenous species: Optimizing surveillance, incorporating advanced technologies, and identifying research needs. J Environ Manage. 2017;202:299–310.

19. Yemshanov D, Haight RG, Koch FH, Venette RC, Swystun T, Fournier RE, et al. Optimizing surveillance strategies for early detection of invasive alien species. Ecol Econ. 2019;162:87–99.

20. Epanchin-Niell RS, Haight RG, Berec L, Kean JM, Liebhold AM. Optimal surveillance and eradication of invasive species in heterogeneous landscapes. Ecol Lett. 2012;15:803–12.

21. Low-Choy S. Getting the Story Straight: Laying the Foundations for Statistical Evaluation of the Performance of Surveillance. In: Jarrad F, Low-Choy S, Mengersen K, editors. Biosecurity Surveillance Quant approaches. 6th ed. CABI; 2015. p. 43–73.

22. Whittle PJL, Stoklosa R, Barrett S, Jarrad FC, Majer JD, Martin PAJ, et al. A method for designing complex biosecurity surveillance systems: Detecting non-indigenous species of invertebrates on Barrow Island. Divers Distrib. 2013;19:629–39.

23. Davidovitch L, Stoklosa R, Majer J, Nietrzeba A, Whittle P, Mengersen K, et al. Info-gap theory and robust design of surveillance for invasive species: The case study of Barrow Island. J Environ Manage. 2009;90:2785–93.

24. Hodgetts J, Ostojá-Starzewski JC, Prior T, Lawson R, Hall J, Boonham N. DNA barcoding for biosecurity: case studies from the UK plant protection program. Genome. 2016;59:1033–48.

25. Armstrong KF, Ball SL. DNA Barcodes for Biosecurity: Invasive Species Identification. Philos Trans Biol Sci. 2005;360:1813–23.

26. European and Mediterranean Plant Protection Organization. PM 7/129 (1) DNA barcoding as an identification tool for a number of regulated pests. EPPO Bull. 2016;46:501–37.

27. Armstrong K. DNA barcoding: A new module in New Zealand's plant biosecurity diagnostic toolbox. EPPO Bull. 2010;40:91–100.

28. Anderson C, Low-Choy S, Whittle P, Taylor S, Gambley C, Smith L, et al. Australian plant biosecurity surveillance systems. Crop Prot. 2017;100:8–20.

29. Raghu S, Hulsman K, Clarke AR, Drew RAI. A rapid method of estimating cathes of abundant fruit fly species (Diptera: Tephritidae) in modified Steiner traps. Aust J Entomol. 2000;39:15–9.

30. Morais P, Reichard M. Cryptic invasions: A review. Sci Total Environ. 2018;613–614:1438–48.

31. Taberlet P, Coissac E, Pompanon F, Brochmann C, Willerslev E. Towards next-generation biodiversity assessment using DNA metabarcoding. Mol Ecol. 2012;21:2045–50.

32. Bik HM, Porazinska DL, Creer S, Caporaso JG, Knight R, Thomas WK. Sequencing our way towards understanding global eukaryotic biodiversity. Trends Ecol Evol. 2012;27:233–43.

33. Tedersoo L, Drenkhan R, Anslan S, Morales-Rodriguez C, Cleary M. High-throughput identification and diagnostics of pathogens and pests: overview and practical recommendations. Mol Ecol Resour. 2018;1–30.

34. Porter TM, Hajibabaei M. Scaling up: A guide to high throughput genomic approaches for biodiversity analysis. Mol Ecol. 2018;27:313– 338.

35. Deiner K, Bik HM, Mächler E, Seymour M, Lacoursière-Roussel A, Altermatt F, et al. Environmental DNA metabarcoding: Transforming how we survey animal and plant communities. Mol Ecol. 2017;26:5872–95.

36. Taberlet P, Bonin A, Zinger L, Coissac E. Environmental DNA: For Biodiversity Research and Monitoring. Oxford University Press; 2017. doi:10.1093/oso/9780198767220.001.0001

37. Alberdi A, Aizpurua O, Bohmann K, Gopalakrishnan S, Lynggaard C, Nielsen M, et al. Promises and pitfalls of using high-throughput sequencing for diet analysis. Mol. Ecol. Resour. 2019. p. 327–48.

38. Comtet T, Sandionigi A, Viard F, Casiraghi M. DNA (meta)barcoding of biological invasions: a powerful tool to elucidate invasion processes and help managing aliens. Biol Invasions. 2015;17:905–22.

39. Darling JA, Blum MJ. DNA-based methods for monitoring invasive species: A review and prospectus. Biol Invasions. 2007;9:751–65.

40. Batovska J, Lynch SE, Cogan NOI, Brown K, Darbro JM, Kho EA, et al. Effective mosquito

and arbovirus surveillance using metabarcoding. Mol Ecol Resour. 2018;18:32–40.

41. Simmons M, Tucker A, Chadderton WL, Jerde CL, Mahon AR, Taylor E. Active and passive environmental DNA surveillance of aquatic invasive species. Can J Fish Aquat Sci. 2016;73:76–83.

42. Lawson Handley L. How will the “molecular revolution” contribute to biological recording? Biol J Linn Soc. 2015;115:750–66.

43. Epanchin-Niell RS, Liebhold AM. Benefits of invasion prevention: Effect of time lags, spread rates, and damage persistence. Ecol Econ. Elsevier B.V.; 2015;116:146–53.

44. Blackburn TM, Essl F, Evans T, Hulme PE, Jeschke JM, Kühn I, et al. A Unified Classification of Alien Species Based on the Magnitude of their Environmental Impacts. PLoS Biol. 2014;12:e1001850.

45. Deagle BE, Thomas AC, McInnes JC, Clarke LJ, Vesterinen EJ, Clare EL, et al. Counting with DNA in metabarcoding studies: How should we convert sequence reads to dietary data? Mol Ecol. 2019;28:391–406.

46. Bilodeau P, Roe AD, Bilodeau G, Blackburn GS, Cui M, Cusson M, et al. Biosurveillance of forest insects: part II—adoption of genomic tools by end user communities and barriers to integration. J Pest Sci. 2019;92:71–82.

47. European and Mediterranean Plant Protection Organization. PM 7/76 (4) Use of EPPO diagnostic protocols. EPPO Bull. 2017;47:7–9.

48. World Trade Organization. Sanitary and Phytosanitary Measures. WTO Agareements Ser. 2010.

49. Clover G, Hammons S, Unger JG. International diagnostic protocols for regulated plant pests. EPPO Bull. 2010;40:24–9.

50. Thiermann AB. Globalization, international trade and animal health: The new roles of OIE. Prev Vet Med. 2005. p. 101–8.

51. Olmos A, Boonham N, Candresse T, Gentit P, Giovani B, Kutnjak D, et al. High-throughput sequencing technologies for plant pest diagnosis: challenges and opportunities. EPPO Bull. 2018;48:219–24.

52. Roe AD, Torson AS, Bilodeau G, Bilodeau P, Blackburn GS, Cui M, et al. Biosurveillance of forest insects: part I—integration and application of genomic tools to the surveillance of non-native forest insects. J. Pest Sci. 2019. p. 51–70.

53. FAO. Preparing to use high-throughput sequencing (HTS) technologies as a diagnostic tool for phytosanitary purposes. Commission on Phytosanitary Measures Recommendation No. 8. Rome; 2019.

54. OIE. Standards for high throughput sequencing, bioinformatics and computational genomics. OIE Terr Man. 2018. p. 88–93.

55. Zinger L, Bonin A, Alsos IG, Bálint M, Bik H, Boyer F, et al. DNA metabarcoding—Need for robust experimental designs to draw sound ecological conclusions. Mol Ecol. 2019;28:1857–62.

56. Freeland JR. The importance of molecular markers and primer design when characterizing biodiversity from environmental DNA. Genome. 2017;6:358–74.

57. Folmer O, Black M, Hoeh W, Lutz R, Vrijenhoek R. DNA primers for amplification of mitochondrial cytochrome c oxidase subunit I from diverse metazoan invertebrates. Mol Mar Biol Biotechnol. 1994;3:294–9.

58. Ashfaq M, Hebert PDN, Naaum A. DNA barcodes for bio-surveillance: Regulated and economically important arthropod plant pests. Genome. 2016;59:933–45.

59. Brandon-Mong G-J, Gan H-M, Sing K-W, Lee P-S, Lim P-E, Wilson J-J. DNA metabarcoding of insects and allies: an evaluation of primers and pipelines. Bull Entomol Res. 2015;105:717–27.

60. Hajibabaei M, Smith MA, Janzen DH, Rodriguez JJ, Whitfield JB, Hebert PDN. A minimalist barcode can identify a specimen whose DNA is degraded. Mol Ecol Notes. 2006;6:959–64.

61. Meusnier I, Singer GAC, Landry JF, Hickey DA, Hebert PDN, Hajibabaei M. A universal DNA mini-barcode for biodiversity analysis. BMC Genomics. 2008;9:4–7.

62. Elbrecht V, Braukmann TWA, Ivanova N V, Prosser SWJ, Hajibabaei M, Wright M, et al. Validation of COI metabarcoding primers for terrestrial arthropods. PeerJ Prepr. 2019;7:e27801v1.

63. Deagle BE, Jarman SN, Coissac E, Pompanon F, Taberlet P. DNA metabarcoding and the cytochrome c oxidase subunit I marker: Not a perfect match. Biol Lett. 2014;10:20140562.

64. Piñol J, Mir G, Gomez-Polo P, Agustí N. Universal and blocking primer mismatches limit the use of high-throughput DNA sequencing for the quantitative metabarcoding of arthropods. Mol Ecol Resour. 2015;15:819–30.

65. Song H, Moulton MJ, Whiting MF. Rampant nuclear insertion of mtDNA across diverse lineages with in Orthoptera (Insecta). PLoS One. 2014;9:e110508.

66. Hlaing T, Tun-Lin W, Somboon P, Socheat D, Setha T, Min S, et al. Mitochondrial pseudogenes in the nuclear genome of Aedes aegypti mosquitoes: Implications for past and future population genetic studies. BMC Genet. 2009;10:1–12.

67. Blacket MJ, Semeraro L, Malipatil MB. Barcoding Queensland Fruit Flies (Bactrocera tryoni): Impediments and improvements. Mol Ecol Resour. 2012;12:428–36.

68. Bensasson D, Zhang DX, Hartl DL, Hewitt GM. Mitochondrial pseudogenes: Evolution's misplaced witnesses. Trends Ecol Evol. 2001;16:314–21.

69. Song H, Buhay JE, Whiting MF, Crandall KA. Many species in one: DNA barcoding overestimates the number of species when nuclear mitochondrial pseudogenes are coamplified. Proc Natl Acad Sci. 2008;105:13486–91.

70. Jiang F, Jin Q, Liang L, Zhang AB, Li ZH. Existence of species complex largely reduced barcoding success for invasive species of Tephritidae: A case study in Bactrocera spp. Mol Ecol Resour. 2014;14:1114–28.

71. Clarke LJ, Soubrier J, Weyrich LS, Cooper A. Environmental metabarcodes for insects: In silico PCR reveals potential for taxonomic bias. Mol Ecol Resour. 2014;14:1160–70.

72. Gillespie JJ, Johnston JS, Cannonone JJ, Gutell RR. Characteristics of the nuclear (18S, 5.8S, 28S and 5S) and mitochondrial (12S and 16S) rRNA genes of Apis mellifera (Insecta: Hymenoptera): structure, organization, and retrotransposable elements. Insect Mol Biol. 2006;15:657–86.

73. Zaidi F, Wei S, Shi M, Chen X. Utility of Multi-Gene Loci for Forensic Species Diagnosis of Blowflies. J Insect Sci. 2011;11:59.

74. Axtner J, Crampton-platt A, Lisa AH, Mohamed A, Xu CCY, Yu DW, et al. An efficient and robust laboratory workflow and tetrapod database for larger scale environmental DNA studies. Gigascience. 2019 Apr 1;8(4). pii: giz029. doi: 10.1093/gigascience/giz029.

75. Zhang GK, Chain FJJ, Abbott CL, Cristescu ME. Metabarcoding using multiplexed markers increases species detection in complex zooplankton communities. Evol Appl. 2018;11:1901–14.

76. De Barba M, Miquel C, Boyer F, Mercier C, Rioux D, Coissac E, et al. DNA metabarcoding multiplexing and validation of data accuracy for diet assessment: Application to omnivorous diet. Mol Ecol Resour. 2014;14:306–23.

77. Alberdi A, Aizpurua O, Gilbert MTP, Bohmann K. Scrutinizing key steps for reliable metabarcoding of environmental samples. Methods Ecol Evol. 2018;9:134–47.

78. Krosch MN, Schutze MK, Strutt F, Clarke AR, Cameron SL. A transcriptome-based analytical workflow for identifying loci for species diagnosis: A case study with Bactrocera fruit flies (Diptera: Tephritidae). Austral Entomol. 2017;58:395– 408.

79. Floyd R, Lima J, de Waard J, Humble L, Hanner R. Common goals: Policy implications of DNA barcoding as a protocol for identification of arthropod pests. Biol Invasions. 2010;12:2947–54.

80. Andújar C, Arribas P, Yu DW, Vogler AP, Emerson BC. Why the COI barcode should be the community DNA metabarcode for the metazoa. Mol Ecol. 2018;27:3968–75.

81. Boykin LM, Armstrong K, Kubatko L, De Barro P. DNA barcoding invasive insects: Database roadblocks. Invertebr Syst. 2012;26:506–14.

82. Ratnasingham S, Hebert PDN. BOLD: The Barcode of Life Data System (www.barcodinglife.org). Mol Ecol Notes. 2007;7:355–64.

83. Benson DA, Cavanaugh M, Clark K, Karsch-Mizrachi I, Ostell J, Pruitt KD, et al. GenBank. Nucleic Acids Res. 2018;46:D41–7.

84. Porter TM, Hajibabaei M. Over 2.5 million sequences in GenBank and Growing. PLoS One. 2018;13:e0200177.

85. Liu S, Yang C, Zhou C, Zhou X. Filling reference gaps via assembling DNA barcodes using high-throughput sequencing-Moving toward barcoding the world. Gigascience. 2017 Dec 1;6(12):1-8. doi: 10.1093/gigascience/gix104.

86. Shen YY, Chen X, Murphy RW. Assessing DNA Barcoding as a Tool for Species Identification and Data Quality Control. PLoS One. 2013;8:e57125.

87. Mioduchowska M, Jan M, Gołdyn B, Kur J, Sell J. Instances of erroneous DNA barcoding of metazoan invertebrates: Are universal cox1 gene primers too "universal"? PLoS One. 2018;13:e0199609.

88. Galan M, Pons JB, Tournayre O, Pierre É, Leuchtmann M, Pontier D, et al. Metabarcoding for the parallel identification of several hundred predators and their prey: Application to bat species diet analysis. Mol Ecol Resour. 2018;18:474–89.

89. Bengtsson-Palme J, Boulund F, Edström R, Feizi A, Johnning A, Jonsson VA, et al. Strategies to improve usability and preserve accuracy in biological sequence databases. Proteomics. 2016;16:2454–60.

90. Batovska J, Blacket MJ, Brown K, Lynch SE. Molecular identification of mosquitoes (Diptera: Culicidae) in southeastern Australia. Ecol Evol. 2016;6:3001–11.

91. Collins RA, Cruickshank RH. The seven deadly sins of DNA barcoding. Mol Ecol Resour. 2013;13:969–75.

92. Castalanelli MA, Severtson DL, Brumley CJ, Szito A, Foottit RG, Grimm M, et al. A rapid non-destructive DNA extraction method for insects and other arthropods. J Asia Pac Entomol. 2010;13:243–8.

93. Carew ME, Nichols SJ, Batovska J, St Clair R, Murphy NP, Blacket MJ, et al. A DNA barcode database of Australia's freshwater macroinvertebrate fauna. Mar Freshw Res. 2017;68:1788–802.

94. Kocher A, Gantier JC, Gaborit P, Zinger L, Holota H, Valiere S, et al. Vector soup: high-throughput identification of Neotropical phlebotomine sand flies using metabarcoding. Mol Ecol Resour. 2017;17:172–82.

95. Bergqvist J, Forsman O, Larsson P, Näslund J, Lilja T, Engdahl C, et al. Detection and Isolation of Sindbis Virus from Mosquitoes Captured During an Outbreak in Sweden, 2013. Vector-Borne Zoonotic Dis. 2015;15:133–40.

96. Somervuo P, Koskela S, Pennanen J, Henrik Nilsson R, Ovaskainen O. Unbiased probabilistic taxonomic classification for DNA barcoding. Bioinformatics. 2016;32:2920–7.

97. Bokulich NA, Kaehler BD, Rideout JR, Dillon M, Bolyen E, Knight R, et al. Optimizing taxonomic classification of marker-gene amplicon sequences with QIIME 2's q2-feature-classifier plugin. Microbiome. 2018;6:90.

98. Rodgers TW, Xu CCY, Giacalone J, Kapheim KM, Saltonstall K, Vargas M, et al. Carrion fly-derived DNA metabarcoding is an effective tool for mammal surveys: Evidence from a known tropical mammal community. Mol Ecol Resour. 2017;17:e133–45.

99. Machida RJ, Leray M, Ho SL, Knowlton N. Data Descriptor: Metazoan mitochondrial gene sequence reference datasets for taxonomic assignment of environmental samples. Sci Data. 2017;4:170027.

100. Richardson R, Bengtsson-Palme J, Gardiner MM, Johnson RM. A reference cytochrome c oxidase subunit I database curated for hierarchical classification of arthropod metabarcoding data. PeerJ. 2018;6:e5126.

101. Porter TM, Hajibabaei M. Automated high throughput animal CO1 metabarcode classification. Sci Rep. 2018;8:4226.

102. Kozlov AM, Zhang J, Yilmaz P, Glöckner FO, Stamatakis A. Phylogeny-aware identification and correction of taxonomically mislabeled sequences. Nucleic Acids Res. 2016;44:5022–33.

103. Chiu CY, Miller SA. Clinical metagenomics. Nat Rev Genet. 2019;20:341–355.

104. Pawluczyk M, Weiss J, Links MG, Egaña Aranguren M, Wilkinson MD, Egea-Cortines M. Quantitative evaluation of bias in PCR amplification and next-generation sequencing derived from metabarcoding samples. Anal Bioanal Chem. 2015;407:1841–8.

105. Piñol J, Senar MA, Symondson WOC. The choice of universal primers and the characteristics of the species mixture determines when DNA metabarcoding can be quantitative. Mol Ecol. 2019;28:407– 419.

106. Rennstam Rubbmark O, Sint D, Horngacher N, Traugott M. A broadly-applicable COI primer pair and an efficient single tube amplicon library preparation protocol for metabarcoding. Ecol Evol. 2018;8:12335– 12350.

107. Bylemans J, Gleeson DM, Hardy CM, Furlan E. Toward an ecoregion scale evaluation of eDNA metabarcoding primers: A case study for the freshwater fish biodiversity of the Murray-Darling Basin (Australia). Ecol Evol. 2018;8:8697–712.

108. Ficetola GF, Coissac E, Zundel S, Riaz T, Shehzad W, Bessière J, et al. An In silico approach for the evaluation of DNA barcodes. BMC Genomics. 2010;11:434.

109. Elbrecht V, Leese F. Validation and Development of COI Metabarcoding Primers for Freshwater Macroinvertebrate Bioassessment. Front Environ Sci. 2017;5:11.

110. Elbrecht V, Leese F. PrimerMiner: an r package for development and in silico validation of DNA metabarcoding primers. Methods Ecol Evol. 2017;8:622–6.

111. Marquina D, Andersson AF, Ronquist F. New mitochondrial primers for metabarcoding of insects, designed and evaluated using in silico methods. Mol Ecol Resour. 2018;325688.

112. Corse E, Tougard C, Archambaud-Suard G, Agnèse JF, Messu Mandeng FD, Bilong Bilong CF, et al. One-locus-several-primers: A strategy to improve the taxonomic and haplotypic coverage in diet metabarcoding studies. Ecol Evol. 2019;9:4603–20.

113. Krehenwinkel H, Wolf M, Lim JY, Rominger AJ, Simison WB, Gillespie RG. Estimating and mitigating amplification bias in qualitative and quantitative arthropod metabarcoding. Sci Rep. 2017;7:17668.

114. Nichols R V, Vollmers C, Newsom LA, Wang Y, Heintzman PD, Leighton M, et al. Minimizing polymerase biases in metabarcoding. Mol Ecol Resour. 2018;18:927– 939.

115. Krehenwinkel H, Pomerantz A, Henderson JB, Kennedy SR, Lim JY, Swamy V, et al. Nanopore sequencing of long ribosomal DNA amplicons enables portable and simple biodiversity assessments with high phylogenetic resolution across broad taxonomic scale. Gigascience. 2019 May 1;8(5). pii: giz006. doi: 10.1093/gigascience/giz006..

116. Elbrecht V, Peinert B, Leese F. Sorting things out: Assessing effects of unequal specimen biomass on DNA metabarcoding. Ecol Evol. 2017;7:6918–26.

117. Braukmann TWA, Ivanova N V., Prosser SWJ, Elbrecht V, Steinke D, Ratnasingham S, et al. Metabarcoding a Diverse Arthropod Mock Community. Mol Ecol Resour. 2019;19:711–27.

118. Thomas AC, Deagle BE, Eveson JP, Harsch CH, Trites AW. Quantitative DNA metabarcoding: Improved estimates of species proportional biomass using correction factors derived from control material. Mol Ecol Resour. 2016;16:714–26.

119. Mclaren MR, Willis AD, Callahan BJ. Consistent and correctable bias in metagenomic sequencing experiments. bioRxiv. 2019;559831.

120. Silverman JD, Bloom RJ, Jiang S, Durand HK, Mukherjee S, David LA. Measuring and Mitigating PCR Bias in Microbiome Data. bioRxiv. 2019;604025.

121. Crampton-Platt A, Yu DW, Zhou X, Vogler AP. Mitochondrial metagenomics: letting the genes out of the bottle. Gigascience. 2016 Mar 22;5:15. doi: 10.1186/s13742-016-0120-y.

122. Gómez-Rodríguez C, Crampton-Platt A, Timmermans MJTN, Baselga A, Vogler AP. Validating the power of mitochondrial metagenomics for community ecology and phylogenetics of complex assemblages. Methods Ecol Evol. 2015;6:883–94.

123. Tang M, Hardman CJ, Ji Y, Meng G, Liu S, Tan M, et al. High-throughput monitoring of wild bee diversity and abundance via mitogenomics. Methods Ecol Evol. 2015;6:1034–43.

124. Linard B, Crampton-Platt A, Moriniere J, Timmermans MJTN, Andújar C, Arribas P, et al. The contribution of mitochondrial metagenomics to large-scale data mining and phylogenetic analysis of Coleoptera. Mol Phylogenet Evol. 2018;128:1–11.

125. Papadopoulou A, Taberlet P, Zinger L. Metagenome skimming for phylogenetic community ecology: A new era in biodiversity research. Mol Ecol. 2015;24:3515–7.

126. Arribas P, Andújar C, Hopkins K, Shepherd M, Vogler AP. Metabarcoding and mitochondrial metagenomics of endogean arthropods to unveil the mesofauna of the soil. Methods Ecol Evol. 2016;7:1071–81.

127. Mamanova L, Coffey AJ, Scott CE, Kozarewa I, Turner EH, Kumar A, et al. Target-enrichment strategies for next-generation sequencing. Nat Methods. 2010;7:111–8.

128. Jones MR, Good JM. Targeted capture in evolutionary and ecological genomics. Mol Ecol. 2016;25:185–202.

129. Macher JN, Zizka VMA, Weigand AM, Leese F. A simple centrifugation protocol for metagenomic studies increases mitochondrial DNA yield by two orders of magnitude. Methods Ecol Evol. 2018;9:1070–4.

130. Dowle EJ, Pochon X, C. Banks J, Shearer K, Wood SA. Targeted gene enrichment and high-throughput sequencing for environmental biomonitoring: a case study using freshwater macroinvertebrates. Mol Ecol Resour. 2016;16:1240–54.

131. Wilcox TM, Zarn KE, Piggott MP, Young MK, McKelvey KS, Schwartz MK. Capture enrichment of aquatic environmental DNA: A first proof of concept. Mol Ecol Resour. 2018;18:1392–401.

132. Peñalba J V., Smith LL, Tonione MA, Sass C, Hykin SM, Skipwith PL, et al. Sequence capture using PCR-generated probes: A cost-effective method of targeted high-throughput sequencing for nonmodel organisms. Mol Ecol Resour. 2014;14:1000–10.

133. Liu S, Wang X, Xie L, Tan M, Li Z, Su X, et al. Mitochondrial capture enriches mito-DNA 100 fold, enabling PCR-free mitogenomics biodiversity analysis. Mol Ecol Resour. 2016;16:470–9.

134. Wilson JJ, Brandon-Mong GJ, Gan HM, Sing KW. High-throughput terrestrial biodiversity assessments: mitochondrial metabarcoding, metagenomics or metatranscriptomics? Mitochondrial DNA Part A. 2019;30:490–9.

135. Poland TM, Rassati D. Improved biosecurity surveillance of non-native forest insects: a review of current methods. J Pest Sci. 2019;92:37–49.

136. Bulman SR, McDougal RL, Hill K, Lear G. Opportunities and limitations for DNA metabarcoding in Australasian plant-pathogen biosecurity. Australas Plant Pathol. Australasian Plant Pathology; 2018;47:467–74.

137. Gardy JL, Loman NJ. Towards a genomics-informed, real-time, global pathogen

surveillance system. Nat Rev Genet. 2018;19:9–20.

138. Batovska J, Lynch SE, Rodoni BC, Sawbridge TI, Cogan NO. Metagenomic arbovirus detection using MinION nanopore sequencing. J Virol Methods. Elsevier; 2017;249:79–84.

139. Gibson J, Shokralla S, Porter TM, King I, van Konynenburg S, Janzen DH, et al. Simultaneous assessment of the macrobiome and microbiome in a bulk sample of tropical arthropods through DNA metasystematics. Proc Natl Acad Sci. 2014;111:8007–12.

140. Whitfield AE, Falk BW, Rotenberg D. Insect vector-mediated transmission of plant viruses. Virology. 2015;479–480:278–89.

141. Miller KE, Hopkins K, Inward DJG, Vogler AP. Metabarcoding of fungal communities associated with bark beetles. Ecol Evol. 2016;6:1590–600.

142. Orlovskis Z, Canale MC, Thole V, Pecher P, Lopes JRS, Hogenhout SA. Insect-borne plant pathogenic bacteria: Getting a ride goes beyond physical contact. Curr Opin Insect Sci. 2015;9:16–23.

143. Sint D, Raso L, Traugott M. Advances in multiplex PCR: Balancing primer efficiencies and improving detection success. Methods Ecol Evol. 2012;3:898–905.

144. Callahan BJ, Wong J, Heiner C, Oh S, Theriot CM, Gulati AS, McGill SK, Dougherty MK. High-throughput amplicon sequencing of the full-length 16S rRNA gene with single-nucleotide resolution. Nucleic Acids Res. 2019 Jul 3. pii: gkz569. doi: 10.1093/nar/gkz569.

145. Tedersoo L, Anslan S. Towards PacBio-based pan-eukaryote metabarcoding using full-length ITS sequences. Environ Microbiol Rep. 2019;

146. Arulandhu AJ, Staats M, Hagelaar R, Voorhuijzen MM, Prins TW, Scholtens I, et al. Development and validation of a multi-locus DNA metabarcoding method to identify endangered species in complex samples. Gigascience. 2017 Oct 1;6(10):1-18. doi: 10.1093/gigascience/gix080.

147. Swift JF, Lance RF, Guan X, Britzke ER, Lindsay DL, Edwards CE. Multifaceted DNA metabarcoding: Validation of a noninvasive, next-generation approach to studying bat populations. Evol Appl. 2018;11:1120–38.

148. Daborn PJ. A Single P450 Allele Associated with Insecticide Resistance in Drosophila. Science (80-). 2002;297:2253–6.

149. Stapley J, Santure AW, Dennis SR. Transposable elements as agents of rapid adaptation may explain the genetic paradox of invasive species. Mol Ecol. 2015;24:2241–52.

150. Ricciardi A, Blackburn TM, Carlton JT, Dick JTA, Hulme PE, Iacarella JC, et al. Invasion Science: A Horizon Scan of Emerging Challenges and Opportunities. Trends Ecol Evol. 2017;32:464–74.

151. Saitoh S, Aoyama H, Fujii S, Sunagawa H, Nagahama H, Akutsu M, et al. A quantitative protocol for DNA metabarcoding of springtails (Collembola). Genome. 2016;59:705–23.

152. Elbrecht V, Leese F. Can DNA-based ecosystem assessments quantify species abundance? Testing primer bias and biomass-sequence relationships with an innovative metabarcoding protocol. PLoS One. 2015;10:e0130324.

153. Gohl DM, Vangay P, Garbe J, MacLean A, Hauge A, Becker A, et al. Systematic improvement of amplicon marker gene methods for increased accuracy in microbiome studies. Nat Biotechnol. 2016;34:942–9.

154. Sinha R, Stanley G, Gulati GS, Ezran C, Travaglini KJ, Wei E, et al. Index Switching Causes "Spreading-Of-Signal" Among Multiplexed Samples In Illumina HiSeq 4000 DNA Sequencing. bioRxiv. 2017;125724.

155. Wick RR, Judd LM, Holt KE. Deepbinner : Demultiplexing barcoded Oxford Nanopore reads with deep convolutional neural networks. PLoS Comput Biol. 2018;14:e1006583.

156. Carlsen T, Aas AB, Lindner D, Vrålstad T, Schumacher T, Kauserud H. Don't make a mista(g)ke: Is tag switching an overlooked source of error in amplicon pyrosequencing studies? Fungal Ecol. 2012;5:747–9.

157. Kircher M, Sawyer S, Meyer M. Double indexing overcomes inaccuracies in multiplex sequencing on the Illumina platform. Nucleic Acids Res. 2012;40:1–8.

158. Tedersoo L, Tooming-Klunderud A, Anslan S. PacBio metabarcoding of Fungi and other eukaryotes: errors, biases and perspectives. New Phytol. 2018;217:1370–85.

159. Costello M, Fleharty M, Abreu J, Farjoun Y, Ferriera S, Holmes L, et al. Characterization and remediation of sample index swaps by non-redundant dual indexing on massively parallel sequencing platforms. BMC Genomics. 2018;19:1–10.

160. Li Q, Zhao X, Zhang W, Wang L, Wang J, Xu D, et al. Reliable multiplex sequencing with rare index mis-assignment on DNB-based NGS platform. BMC Genomics. 2019;20:1–13.

161. Illumina. Effects of Index Misassignment on Multiplexing and Downstream Analysis. 2017.

162. Schnell IB, Bohmann K, Gilbert MTP. Tag jumps illuminated - reducing sequence-to-sample misidentifications in metabarcoding studies. Mol Ecol Resour. 2015;15:1289–303.

163. Hanna RE, Doench JG. A case of mistaken identity. Nat Biotechnol. 2018;36:802–4.

164. Nguyen NH, Smith D, Peay K, Kennedy P. Parsing ecological signal from noise in next generation amplicon sequencing. New Phytol. 2015;205:1389–93.

165. MacConaill LE, Burns RT, Nag A, Coleman HA, Slevin MK, Giorda K, et al. Unique, dual-indexed sequencing adapters with UMIs effectively eliminate index cross-talk and significantly improve sensitivity of massively parallel sequencing. BMC Genomics. 2018;19:30.

166. Bartram J, Mountjoy E, Brooks T, Hancock J, Williamson H, Wright G, et al. Accurate Sample Assignment in a Multiplexed, Ultrasensitive, High-Throughput Sequencing Assay for Minimal Residual Disease. J Mol Diagnostics. 2016;18:494–506.

167. Faircloth BC, Glenn TC. Not all sequence tags are created equal: designing and validating sequence identification tags robust to indels. PLoS One. 2012;7:e42543.

168. Goodwin S, McPherson JD, McCombie WR. Coming of age: Ten years of next-generation sequencing technologies. Nat Rev Genet. 2016;17:333–51.

169. Bleidorn C. Third generation sequencing: Technology and its potential impact on evolutionary biodiversity research. Syst Biodivers. 2016;14:1–8.

170. Benítez-Páez A, Portune KJ, Sanz Y. Species-level resolution of 16S rRNA gene amplicons sequenced through the MinION™ portable nanopore sequencer. Gigascience. 2016;5:4.

171. van Dijk EL, Jaszczyszyn Y, Naquin D, Thermes C. The Third Revolution in Sequencing Technology. Trends Genet. 2018;34:666–81.

172. Hebert PDN, Braukmann TWA, Prosser SWJ, Ratnasingham S, DeWaard JR, Ivanova N V., et al. A Sequel to Sanger: Amplicon sequencing that scales. BMC Genomics. 2018;19:1–14.

173. Calus ST, Ijaz UZ, Pinto AJ. NanoAmpli-Seq: A workflow for amplicon sequencing for mixed microbial communities on the nanopore sequencing platform. Gigascience. 2018 Dec 1;7(12). doi: 10.1093/gigascience/giy140.

174. Volden R, Palmer T, Byrne A, Cole C, Schmitz RJ, Green RE, et al. Improving nanopore read accuracy with the R2C2 method enables the sequencing of highly multiplexed full-length single-cell cDNA. Proc Natl Acad Sci. 2018;115:9726–31.

175. Murray DC, Coghlan ML, Bunce M. From benchtop to desktop: Important considerations when designing amplicon sequencing workflows. PLoS One. 2015;10:e0124671.

176. Scott R, Zhan A, Brown EA, Chain FJJ, Cristescu ME, Gras R, et al. Optimization and performance testing of a sequence processing pipeline applied to detection of nonindigenous species. Evol Appl. 2018;891–905.

177. Palmer JM, Jusino MA, Banik MT, Lindner DL. Non-biological synthetic spike-in controls and the AMPtk software pipeline improve fungal high throughput amplicon sequencing data. PeerJ. 2017;213470.

178. Bolyen E, Rideout JR, Dillon MR, Bokulich NA, Abnet C, Al-Ghalith GA, et al. QIIME 2: Reproducible, interactive, scalable, and extensible microbiome data science. PeerJ Prepr. 2018;6:e27295v2.

179. Schloss PD, Westcott SL, Ryabin T, Hall JR, Hartmann M, Hollister EB, et al. Introducing mothur: Open-source, platform-independent, community-supported software for describing and comparing microbial communities. Appl Environ Microbiol. 2009;75:7537–41.

180. Boyer F, Mercier C, Bonin A, Le Bras Y, Taberlet P, Coissac E. obitools: A unix-inspired software package for DNA metabarcoding. Mol Ecol Resour. 2016;16:176–82.

181. Rognes T, Flouri T, Nichols B, Quince C, Mahé F. VSEARCH: a versatile open source tool for metagenomics. PeerJ. 2016;4:e2584.

182. Pauvert C, Buée M, Laval V, Edel-Hermann V, Fauchery L, Gautier A, et al. Bioinformatics matters: The accuracy of plant and soil fungal community data is highly dependent on the metabarcoding pipeline. Fungal Ecol. 2019;41:23–33.

183. Flynn JM, Brown EA, Chain FJJ, Macisaac HJ, Cristescu ME. Toward accurate molecular identification of species in complex environmental samples: Testing the performance of sequence filtering and clustering methods. Ecol Evol. 2015;5:2252–66.

184. Majaneva M, Hyytiäinen K, Varvio SL, Nagai S, Blomster J. Bioinformatic amplicon read processing strategies strongly affect eukaryotic diversity and the taxonomic composition of communities. PLoS One. 2015;10:e0130035.

185. Salk JJ, Schmitt MW, Loeb LA. Enhancing the accuracy of next-generation sequencing for detecting rare and subclonal mutations. Nat Rev Genet. 2018;19:269–85.

186. Edgar RC, Flyvbjerg H. Error filtering, pair assembly and error correction for next-generation sequencing reads. Bioinformatics. 2015;31:3476–82.

187. Ewing B, Hillier LD, Wendl MC. Base-Calling of Automated Sequencer Traces Using Phred. Genome Res. 1998;8:186–94.

188. Bokulich NA, Subramanian S, Faith JJ, Gevers D, Gordon JI, Knight R, et al. Quality-filtering vastly improves diversity estimates from Illumina amplicon sequencing. Nat Methods. 2013;10:57–9.

189. Potapov V, Ong JL. Examining sources of error in PCR by single-molecule sequencing. PLoS One. 2017;12:e0169774.

190. Elbrecht V, Hebert PDN, Steinke D. Slippage of degenerate primers can cause variation in amplicon length. Sci Rep. 2018;8:10999.

191. Schirmer M, Ijaz UZ, D'Amore R, Hall N, Sloan WT, Quince C. Insight into biases and sequencing errors for amplicon sequencing with the Illumina MiSeq platform. Nucleic Acids Res. 2015;43:e37.

192. Meyer CP, Paulay G. DNA barcoding: Error rates based on comprehensive sampling. PLoS Biol. 2005;3:1–10.

193. Hebert PDN, Ratnasingham S, de Waard JR. Barcoding animal life: cytochrome c oxidase subunit 1 divergences among closely related species. Proc R Soc B Biol Sci. 2003;270:S96–9.

194. Callahan BJ, McMurdie PJ, Holmes SP. Exact sequence variants should replace operational

taxonomic units in marker-gene data analysis. ISME J. 2017;11:2639–43.

195. Tedersoo L, Ramirez KS, Nilsson RH, Kaljuvee A, Kõljalg U, Abarenkov K. Standardizing metadata and taxonomic identification in metabarcoding studies. Gigascience. 2015;4:34.

196. Callahan BJ, McMurdie PJ, Rosen MJ, Han AW, Johnson AJA, Holmes SP. DADA2: High-resolution sample inference from Illumina amplicon data. Nat Methods. 2016;13:581–3.

197. Amir A, Daniel M, Navas-Molina J, Kopylova E, Morton J, Xu ZZ, et al. Deblur Rapidly Resolves Single-Nucleotide Community Sequence Patterns. mSystems. 2017;2:e00191-16.

198. Edgar RC. UNOISE2: improved error-correction for Illumina 16S and ITS amplicon sequencing. bioRxiv. 2016;081257.

199. Marshall NT, Stepien CA. Invasion genetics from eDNA and thousands of larvae: A targeted metabarcoding assay that distinguishes species and population variation of zebra and quagga mussels. Ecol Evol. 2019;9:3515–38.

200. Edgar RC, Haas BJ, Clemente JC, Quince C, Knight R. UCHIME improves sensitivity and speed of chimera detection. Bioinformatics. 2011;27:2194–200.

201. Haas BJ, Gevers D, Earl AM, Feldgarden M, Ward D V, Giannoukos G, et al. Chimeric 16S rRNA Sequence Formation and Detection in Sanger and 454-Pyrosequenced PCR Amplicons. Genome Res. 2011;21:494–504.

202. Brown EA, Chain FJJ, Crease TJ, Macisaac HJ, Cristescu ME. Divergence thresholds and divergent biodiversity estimates: Can metabarcoding reliably describe zooplankton communities? Ecol Evol. 2015;5:2234–51.

203. Decelle J, Romac S, Sasaki E, Not F, Mahé F. Intracellular diversity of the V4 and V9 regions of the 18S rRNA in marine protists (radiolarians) assessed by high-throughput sequencing. PLoS One. 2014;9:e104297.

204. Turon X, Antich A, Palacín C, Præbel K, Wangensteen OS. From metabarcoding to metaphylogeography: separating the wheat from the chaff. bioRxiv. 2019;629535.

205. Olds BP, Jerde CL, Renshaw MA, Li Y, Evans NT, Turner CR, et al. Estimating species richness using environmental DNA. Ecol Evol. 2016;6:4214–26.

206. Gardner PP, Watson RJ, Morgan XC, Draper JL, Finn RD, Morales SE, et al. Identifying accurate metagenome and amplicon software via a meta-analysis of sequence to taxonomy benchmarking studies. PeerJ. 2019;7:e6160.

207. Bazinet AL, Cummings MP. A comparative evaluation of sequence classification programs. BMC Bioinformatics. 2012;13:92.

208. Altschul SF, Gish W, Miller W, Myers EW, Lipman DJ. Basic local alignment search tool. J Mol Biol. 1990;215:403–10.

209. Koski LB, Golding GB. The closest BLAST hit is often not the nearest neighbor. J Mol Evol. 2001;52:540–2.

210. Virgilio M, Backeljau T, Nevado B, De Meyer M. Comparative performances of DNA barcoding across insect orders. BMC Bioinformatics. 2010;11:206.

211. Wang Q, Garrity GM, Tiedje JM, Cole JR. Naive Bayesian classifier for rapid assignment of rRNA sequences into the new bacterial taxonomy. Appl Environ Microbiol. 2007;73:5261–7.

212. Huson D, Auch A, Qi J, Schuster S. MEGAN analysis of metagenome data. Genome Res. 2007;17:377–86.

213. Wilkinson SP, Davy SK, Bunce M, Stat M. Taxonomic identification of environmental DNA with informatic sequence classification trees. PeerJ Prepr. 2018;6:e26812v1.

214. Lan Y, Wang Q, Cole JR, Rosen GL. Using the RDP classifier to predict taxonomic novelty and reduce the search space for finding novel organisms. PLoS One. 2012;7:e32491.

215. Edgar R. SINTAX: a simple non-Bayesian taxonomy classifier for 16S and ITS sequences. bioRxiv. 2016;074161.

216. Somervuo P, Yu DW, Xu CCY, Ji Y, Hultman J, Wirta H, et al. Quantifying uncertainty of taxonomic placement in DNA barcoding and metabarcoding. Methods Ecol Evol. 2017;8:398–407.

217. Burgar JM, Murray DC, Craig MD, Haile J, Houston J, Stokes V, et al. Who's for dinner? High-throughput sequencing reveals bat dietary differentiation in a biodiversity hotspot where prey taxonomy is largely undescribed. Mol Ecol. 2014;23:3605–17.

218. Secretariat of the International Plant Protection Convention (IPPC). ISPM 4 Requirements for the establishment of pest free areas. 2017. https://www.ippc.int/en/publications/requirements-establishment-pest-free-areas/

219. Champlot S, Berthelot C, Pruvost M, Andrew Bennett E, Grange T, Geigl EM. An efficient multistrategy DNA decontamination procedure of PCR reagents for hypersensitive PCR applications. PLoS One. 2010;5:e13042.

220. Miller S, Naccache SN, Samayoa E, Messacar K, Arevalo S, Federman S, et al. Laboratory validation of a clinical metagenomic sequencing assay for pathogen detection in cerebrospinal fluid. Genome Res. 2019;29:831–42.

221. European and Mediterranean Plant Protection Organization Organisation. Basic requirements for quality management in plant pest diagnosis laboratories. EPPO Bull. 2007;37:580–8.

222. Gu W, Miller S, Chiu CY. Clinical Metagenomic Sequencing for Pathogen Detection. Annu Rev Pathol Mech Dis. 2019;14:319–38.

223. Elbrecht V, Steinke D. Scaling up DNA metabarcoding for freshwater macrozoobenthos monitoring. Freshw Biol. 2019;64:380–7.

224. Ficetola GF, Taberlet P, Coissac E. How to limit false positives in environmental DNA and metabarcoding? Mol Ecol Resour. 2016;16:604–7.

225. Klymus KE, Marshall NT, Stepien CA. Environmental DNA (eDNA) metabarcoding assays to detect invasive invertebrate species in the Great Lakes. PLoS One. 2017;12:e0177643.

226. Wilson CC, Wozney KM, Smith CM. Recognizing false positives: Synthetic oligonucleotide controls for environmental DNA surveillance. Methods Ecol Evol. 2016;7:23–9.

227. Ji Y, Huotari T, Roslin T, Martin-Schmidt N, Wang J, Yu D, et al. SPIKEPIPE: A metagenomic pipeline for the accurate quantification of eukaryotic species occurrences and abundances using DNA barcodes or mitogenomes. bioRxiv. 2019;533737.

228. Wright ES, Vetsigian KH. Quality filtering of Illumina index reads mitigates sample cross-talk. BMC Genomics. 2016;17:876.

229. Zepeda-Mendoza ML, Bohmann K, Carmona Baez A, Gilbert MTP. DAMe: A toolkit for the initial processing of datasets with PCR replicates of double-tagged amplicons for DNA metabarcoding analyses. BMC Res Notes. 2016;9:255.

230. Salter SJ, Cox MJ, Turek EM, Calus ST, Cookson WO, Moffatt MF, et al. Reagent and laboratory contamination can critically impact sequence-based microbiome analyses. BMC Biol. 2014;12:87.

231. Davis NM, Proctor DM, Holmes SP, Relman DA, Callahan BJ. Simple statistical identification and removal of contaminant sequences in marker-gene and metagenomics data. Microbiome. 2018;6:226.

232. McKnight DT, Huerlimann R, Bower DS, Schwarzkopf L, Alford RA, Zenger KR. microDecon: A highly accurate read- subtraction tool for the post- sequencing removal of contamination in metabarcoding studies. Environ DNA. 2019;1:14–25.

233. Larsson AJM, Stanley G, Sinha R, Weissman IL, Sandberg R. Computational correction of index switching in multiplexed sequencing libraries. Nat Methods. 2018;15:305–7.

234. Yeh Y-C, Needham DM, Sieradzki ET, Fuhrman JA. Taxon Disappearance from Microbiome Analysis Reinforces the Value of Mock Communities as a Standard in Every Sequencing Run. mSystems. 2018;3:e00023-18.

235. Hardwick SA, Chen WY, Wong T, Kanakamedala BS, Deveson IW, Ongley SE, et al. Synthetic microbe communities provide internal reference standards for metagenome sequencing and analysis. Nat Commun. 2018;9:3096.

236. Leray M, Knowlton N. Random sampling causes the low reproducibility of rare eukaryotic OTUs in Illumina COI metabarcoding. PeerJ. 2017;5:e3006.

237. Ficetola GF, Pansu J, Bonin A, Coissac E, Giguet-Covex C, De Barba M, et al. Replication levels, false presences and the estimation of the presence/absence from eDNA metabarcoding data. Mol Ecol Resour. 2015;15:543–56.

238. Mata VA, Rebelo H, Amorim F, Mccracken GF, Jarman S, Beja P. How much is enough? Effects of technical and biological replication on metabarcoding dietary analysis. Mol Ecol. 2019;28:165–75.

239. Guillera-Arroita G. Modelling of species distributions, range dynamics and communities under imperfect detection: advances, challenges and opportunities. Ecography. 2017;40:281–95.

240. Lahoz-Monfort JJ, Guillera-Arroita G, Tingley R. Statistical approaches to account for false-positive errors in environmental DNA samples. Mol Ecol Resour. 2016;16:673–85.

241. Krehenwinkel H, Fong M, Kennedy S, Huang EG, Noriyuki S, Cayetano L, et al. The effect of DNA degradation bias in passive sampling devices on metabarcoding studies of arthropod communities and their associated microbiota. PLoS One. 2018;13:e0189188.

242. Berec L, Kean JM, Epanchin-Niell R, Liebhold AM, Haight RG. Designing efficient surveys: spatial arrangement of sample points for detection of invasive species. Biol Invasions. 2014;17:445–59.

243. European and Mediterranean Plant Protection Organization. PM 7/98 (2) Specific requirements for laboratories preparing accreditation for a plant pest diagnostic activity. EPPO Bull. 2010;44:117–47.

244. National Association of Testing Authorities. Technical Note 17 - Guidelines for the validation and verification of quantitative and qualitative test methods. 2012.

245. Blaser S, Diem H, von Felten A, Gueuning M, Andreou M, Boonham N, et al. From laboratory to point of entry: development and implementation of a loop-mediated isothermal amplification (LAMP)-based genetic identification system to prevent introduction of quarantine insect species. Pest Manag Sci. 2018;74:1504–12.

246. Schlaberg R, Chiu CY, Miller S, Procop GW, Weinstock G. Validation of metagenomic next-generation sequencing tests for universal pathogen detection. Arch Pathol Lab Med. 2017;141:776–86.

247. Adams IP, Fox A, Boonham N, Massart S, De Jonghe K. The impact of high throughput sequencing on plant health diagnostics. Eur J Plant Pathol. 2018;1–11.

248. Gargis AS, Kalman L, Lubin IM. Assuring the quality of next-generation sequencing in clinical microbiology and public health laboratories. J Clin Microbiol. 2016;54:2857–65.

249. Hatzenbuhler C, Kelly JR, Martinson J, Okum S, Pilgrim E. Sensitivity and accuracy of high-throughput metabarcoding methods for early detection of invasive fish species. Sci Rep. 2017;7:46393.

250. Bell KL, Burgess KS, Botsch JC, Dobbs EK, Read TD, Brosi BJ. Quantitative and

qualitative assessment of pollen DNA metabarcoding using constructed species mixtures. Mol Ecol. 2018;28:431–55.

251. Smith DP, Peay KG. Sequence depth, not PCR replication, improves ecological inference from next generation DNA sequencing. PLoS One. 2014;9:e90234.

252. Landolt PJ, Adams T, Davis TS, Rogg H. Spotted Wing Drosophila, Drosophila suzukii (Diptera: Drosophilidae), trapped with combinations of wines and vinegars. Florida Entomol. 2012;95:326–32.

253. Lindahl T. Instability and decay of the primary structure of DNA. Nature. 1993;362:709–15.

254. Brown EA, Chain FJJ, Zhan A, MacIsaac HJ, Cristescu ME. Early detection of aquatic invaders using metabarcoding reveals a high number of non-indigenous species in Canadian ports. Divers Distrib. 2016;22:1045–59.

255. Clarke AR, Li Z, Qin Y, Zhao Z, Liu L, Schutze MK. Bactrocera dorsalis (Hendel) (Diptera: Tephritidae) is not invasive through Asia: It's been there all along. J Appl Entomol. 2019;00:1–5.

256. Callan SK, Majer JD, Edwards K, Moro D. Documenting the terrestrial invertebrate fauna of Barrow Island, Western Australia. Aust J Entomol. 2011;50:323–43.

257. Massart S, Candresse T, Gil J, Lacomme C, Predajna L, Ravnikar M, et al. A framework for the evaluation of biosecurity, commercial, regulatory, and scientific impacts of plant viruses and viroids identified by NGS technologies. Front Microbiol. 2017;8:45.

258. Carew ME, Coleman RA, Hoffmann AA. Can non-destructive DNA extraction of bulk invertebrate samples be used for metabarcoding? PeerJ. 2018;6:e4980.

259. Ritter CD, Häggqvist S, Karlsson D, Sääksjärvi IE, Muasya AM, Nilsson RH, et al. Biodiversity assessments in the 21st century: the potential of insect traps to complement environmental samples for estimating eukaryotic and prokaryotic diversity using high-throughput DNA metabarcoding. Genome. 2019;62:147–59.

260. Nielsen M, Gilbert MTP, Pape T, Bohmann K. A simplified DNA extraction protocol for unsorted bulk arthropod samples that maintains exoskeletal integrity. Environ DNA. 2019;00:1–11.

261. Martins FMS, Galhardo M, Filipe AF, Teixeira A, Pinheiro P, Paupério J, et al. Have the cake and eat it: Optimising non- destructive DNA metabarcoding of macroinvertebrate samples for freshwater biomonitoring. Mol Ecol Resour. 2019;1755-0998.13012.

262. Zizka VMA, Leese F, Peinert B, Geiger MF. DNA metabarcoding from sample fixative as a quick and voucher-preserving biodiversity assessment method. Genome. 2018;62:122–36.

263. Hajibabaei M, Spall JL, Shokralla S, van Konynenburg S. Assessing biodiversity of a freshwater benthic macroinvertebrate community through non-destructive environmental barcoding of DNA from preservative ethanol. BMC Ecol. 2012;12:28.

264. Linard B, Arribas P, Andújar C, Crampton-Platt A, Vogler AP. Lessons from genome skimming of arthropod-preserving ethanol. Mol Ecol Resour. 2016;16:1365–77.

265. McIntyre ABR, Ounit R, Afshinnekoo E, Prill RJ, Hénaff E, Alexander N, et al. Comprehensive benchmarking and ensemble approaches for metagenomic classifiers. Genome Biol. Genome Biology; 2017;18:1–19.

266. Duncavage EJ, Abel HJ, Pfeifer JD. In Silico Proficiency Testing for Clinical Next-Generation Sequencing. J Mol Diagnostics. 2017;19:35–42.

267. Hardwick SA, Deveson IW, Mercer TR. Reference standards for next-generation sequencing. Nat Rev Genet. 2017;18:473–84.

268. Sinha R, Abu-Ali G, Vogtmann E, Fodor AA, Ren B, Amir A, et al. Assessment of variation in microbial community amplicon sequencing by the Microbiome Quality Control (MBQC) project consortium. Nat Biotechnol. 2017;35:1077–86.

269. Schrijver I, Aziz N, Jennings LJ, Richards CS, Voelkerding K V., Weck KE. Methods-based proficiency testing in molecular genetic pathology. J Mol Diagnostics. 2014;16:283–7.

270. Knight R, Vrbanac A, Taylor BC, Aksenov A, Callewaert C, Debelius J, et al. Best practices for analysing microbiomes. Nat Rev Microbiol. 2018;16:410–22.

271. Latombe G, Pyšek P, Jeschke JM, Blackburn TM, Bacher S, Capinha C, et al. A vision for global monitoring of biological invasions. Biol Conserv. 2017;213:295–308.

272. MacLeod A. The relationship between biosecurity surveillance and risk analysis. In: Jarrad F, Low-Choy S, Mengersen K, editors. Biosecurity Surveillance Quant approaches. CABI; 2015. p. 109–20.

273. Schlick-Steiner BC, Steiner FM, Seifert B, Stauffer C, Christian E, Crozier RH. Integrative Taxonomy: A Multisource Approach to Exploring Biodiversity. Annu Rev Entomol. 2010;55:421–38.

274. Stephens ZD, Lee SY, Faghri F, Campbell RH, Zhai C, Efron MJ, et al. Big data: Astronomical or genomical? PLoS Biol. 2015;13:1–11.

275. Evans DM, Kitson JJN, Lunt DH, Straw NA, Pocock MJO. Merging DNA metabarcoding and ecological network analysis to understand and build resilient terrestrial ecosystems. Funct Ecol. 2016;1904–16.

276. Lafleur JP, Jönsson A, Senkbeil S, Kutter JP. Recent advances in lab-on-a-chip for biosensing applications. Biosens Bioelectron. 2016;76:213–33.

277. Potamitis I, Eliopoulos P, Rigakis I. Automated Remote Insect Surveillance at a Global Scale and the Internet of Things. Robotics. 2017;6:19.

278. Bohan DA, Vacher C, Tamaddoni-Nezhad A, Raybould A, Dumbrell AJ, Woodward G.

Next-Generation Global Biomonitoring: Large-scale, Automated Reconstruction of Ecological Networks. Trends Ecol Evol. 2017;32:477–87.

279. Muschelli J. rscopus: Scopus Database "API" Interface 2018.: https://github.com/muschellij2/rscopus

280. Winter, David J. rentrez: An R package for the NCBI eUtils API. R J. 2019;9:520.

281. Chamberlain S. fulltext: Full Text of "Scholarly" Articles Across Many Data Sources. 2019. https://github.com/ropensci/fulltext/

282. R Core Team. R: A language and environment for statistical computing.. R Foundation for Statistical Computing, Vienna, Austria.; 2017. http://www.r-project.org/

283. Fay C. tidystringdist: String Distance Calculation with Tidy Data Principles. 2019. https://github.com/ColinFay/tidystringdist

284. Wickham H, Francois R, Henry L, Müller K, others. dplyr: A grammar of data manipulation. R Packag version 04. 2015;3.

285. Wickham H. ggplot2: Elegant Graphics for Data Analysis. Springer-Verlag New York; 2016. http://ggplot2.org

286. Plant Health Australia. The National Plant Biosecurity Status Report. 2017.

287. Chamberlain S. bold: Interface to Bold Systems API. 2017. https://cran.r-project.org/package=bold

288. Schöfl G. biofiles: An Interface for GenBank/GenPept Flat Files. https://github.com/gschofl/biofiles

289. Kahle D, Wickham H. ggmap: Spatial Visualization with ggplot2. R J. 2013;5:144–61. http://journal.r-project.org/archive/2013-1/kahle-wickham.pdf

290. Piper, Alexander M. (2019). Supplementary S2: Prospects and challenges of implementing DNA metabarcoding for high throughput insect surveillance (Version 2.0). http://doi.org/10.5281/zenodo.3252736

TABLES

Table 1: Methods employed for insect identification, with suitability assessed according to accuracy, expertise, general applicability, time and throughput criteria

Identification method	Taxonomic expertise	Identify specific taxa	Identify broad range of taxa	Throughput level	Time per identification
**Morphological**					
Microscopic examination	High	High*	High*	Low	Moderate
**Molecular**					
PCR-RFLP	Low	Moderate	Low	Moderate	Moderate
DNA barcoding	Low	High	High	Low	Moderate
qPCR/ddPCR	Low	High	Low	High	Low
LAMP	Low	High	Low	Low	Low
Metabarcoding	Low	High	High	Very High	Low

* This morphological identification score assumes a high level of taxonomic knowledge and a low human error rate.

Table 2: Comparison of sequence throughputs, error rate and associated costs between high-throughput sequencing platforms.

	**Short read platforms**							**Long read platforms**			
	Illumina MiSeq	Illumina NextSeq	Illumina HiSeq 3000/4000	Illumina NovaSeq	MGISeq-200	MGISeq-2000	MGISeq-T7	PacBio Sequel	PacBio Sequel II	ONT MinION	ONT PromethION
**Maximum throughput (Gigabases)**	15Gb	120Gb	750Gb /1500Gb (8/16 lanes)	6000Gb (8 lanes)	60Gbp	1080Gbp	6000Gbp	20Gb	160Gbp	20Gb	150Gb per flow cell (up to 48)
**Maximum Read length**	2x300bp	2x150bp	2x150bp	2x150bp	2x100bp	2x150bp	2x150bp	~100kb	~100kb	~2Mb	~2Mb
**Error rate**	Low	Low	Low	Low	Low	Low	Low	Low (consensus error)	Low (consensus error)	High	High
**Instrument cost**	Low	Medium	High	High	Low	Medium	High	High	High	Extremely Low	Low
**Setup time (labour)**	Medium	Medium	Medium	Medium	Medium	Medium	Medium	High	High	Low	Low
**Run time**	56hrs	30hrs	84hrs	40hrs	<48 hrs	<48 hrs	24 hrs	15hrs	15hrs	1-72hrs	1-72hrs
**Sequencing cost per sample†**	<$50	<$15	<$10	<$5	<$50	<$10	<$5	<$25	<$15	<$25	<$5

*Costs are presented in Australian Dollars (AUD) and consider chemistry cost, depreciation, servicing, and computational cost over the lifespan of the instrument, however total costs and read lengths will further depend on target enrichment and library preparation methods used. †Assuming pooled sequencing of many traps with 250Mb sequencing effort per sample.

Table 3. Recommended quality control checkpoints for metabarcoding based diagnostics.

	Quality control checkpoint	Consequences
**Laboratory preparedness**	Are all reagents within expiry date and stored properly?	Poor reagent storage can lead to reduced efficiency and false-negatives
	Is equipment appropriately maintained and calibrated?	Poorly calibrated equipment will generate inconstancies and inaccurate data
	Have laboratory surfaces been decontaminated?	Dirty laboratories can be a source of DNA contamination, leading to lowered sensitivity or false-positives
	Has swipe testing of laboratory surfaces been conducted?	
**Sample acceptance**	Have specimens arrived in a condition appropriate for extracting DNA?	Inappropriately stored specimens can lead to false-negative results and a reduction in sensitivity
	Are specimens traceable to origin location?	Misidentification of sample origin can complicate detection response
**Nucleic acid extraction**	Is DNA of sufficient quantity and quality?	Insufficient DNA quantity or presence of contaminants can inhibit reactions and result in false-negatives
**Marker enrichment**	Are the correct fragment sizes present for the target barcode marker?	Incorrect fragment sizes could indicate off-target amplification
	Have the positive control samples successfully amplified?	Absence of product in positive controls indicates amplification failure
	Are negative control samples free of DNA fragments?	Visible DNA fragments in negative controls indicates contamination
**Library preparation & multiplexing**	Are libraries of the appropriate size and concentration?	Libraries of significantly different sizes or concentrations will complicate multiplexing
	Have sets of unique-dual indices been used?	Unique-dual indexing is necessary to control for index-switching
	Have index sets been alternated since the previous sequencing run?	Cross-contamination of libraries between sequencing runs can cause false-positives
**High-throughput sequencing**	Has the pooled library been appropriately sized and quantified?	Inaccurate sizing and quantification can cause overloading of flow cell and failed runs, or underloading and low data output
	Has the sequencer been appropriately cleaned between runs?	Insufficient cleaning of the sequencer can result in cross-contamination between runs
**De-multiplexing &**	Has minimum sequencing depth been achieved for each sample?	Low sequencing depth can cause false-negatives

**quality trimming**	Are an appropriate number of reads passing quality filtering?	Low numbers of reads passing quality filters can indicate issues with sequencing run and result in false-negatives
**OTU clustering & denoising**	How much of the original data is explained by the final OTUs/ASVs<br>Have chimeras and sequences with disrupted ORFs been checked for? (for protein coding genes)	Lower than expected sequences can indicate overly restrictive bioinformatics parameters<br>Chimeras and pseudogenes can inflate taxonomic diversity leading to false-positives
**Taxonomic assignment**	Has the reference database been curated to remove mislabelled taxonomy and pseudogenic sequences?<br>Has the taxonomy been applied with appropriate confidence levels?	Mislabelled sequences can lead to both false-positives & false-negatives<br>Low confidence assignment indicates incomplete or issues in reference database
**Interpretation of results**	Have the taxa received an appropriate number of reads to pass detection threshold?<br>Has a minimum detection threshold been applied to remove index-switching?<br>Are there any taxa that need to be confirmed with alternative methods?	Taxa under detection threshold could represent errors that have not been sufficiently controlled for<br>Index-switching can cause spreading of taxa to other samples and result in false-positives<br>Any high-risk putative detections should be confirmed with alternative method before reporting, if possible
**Reporting & sign off**	Have any exceptions to laboratory standard operating procedure (SOP) been made?<br>Has data been stored appropriately?<br>Have results been signed off by competent individual?	Non-compliances with SOP should be highlighted and diagnostic confidence may be reduced<br>Archiving of data is important for re-analysis<br>Incorrect reporting or interpretation of significant taxa can lead to incorrect managment response

Figure Legends

Figure 1- Metabarcoding in the literature

A. Published articles obtained from Scopus, Crossref and PubMed searches on 2019-06-19 for all metabarcoding studies, and those containing keywords in title or abstract relevant to invasive

insect surveillance. **B.** Sequencing platforms used in the above metabarcoding studies displayed as a proportion for each year.

Figure 2- Overview of common metabarcoding workflows for identification of trapped insect species

Figure 3- DNA barcodes on public reference databases

A. Global distribution of all sufficiently annotated DNA barcode records from BOLD and GenBank for all barcode loci; Records for all Insecta are displayed as a density map, while those species present on international pest lists are overlaid in red. **B**. Distribution of records and unique species within major public databases for the 10 barcode markers with the most reference information for entire Insecta and for **C**. Insecta species present on international pest lists.

Figure 4- Unique dual indexing overcomes issues of cross-contamination due to index-switching

A. An amplified barcode locus with sequencing adapters attached, read locations and orientations are indicated for commonly used Illumina platforms. Read 1 and 2 are designed to overlap to facilitate assembly into a consensus sequence. Both sequencing adapters incorporate a unique oligonucleotide index sequence to allow differentiation of multiplexed samples. Strategies for indexing include; **B.** Combinatorial indexing, where indices on either end of the molecule are shared with other samples but the combination of the two is unique, and **C.** Unique dual indexing, where adapter indices at both ends of the molecule are completely unique to the sample.

Figure 1

Click here to access/download;Figure;Figure1_Resubmission.pdf

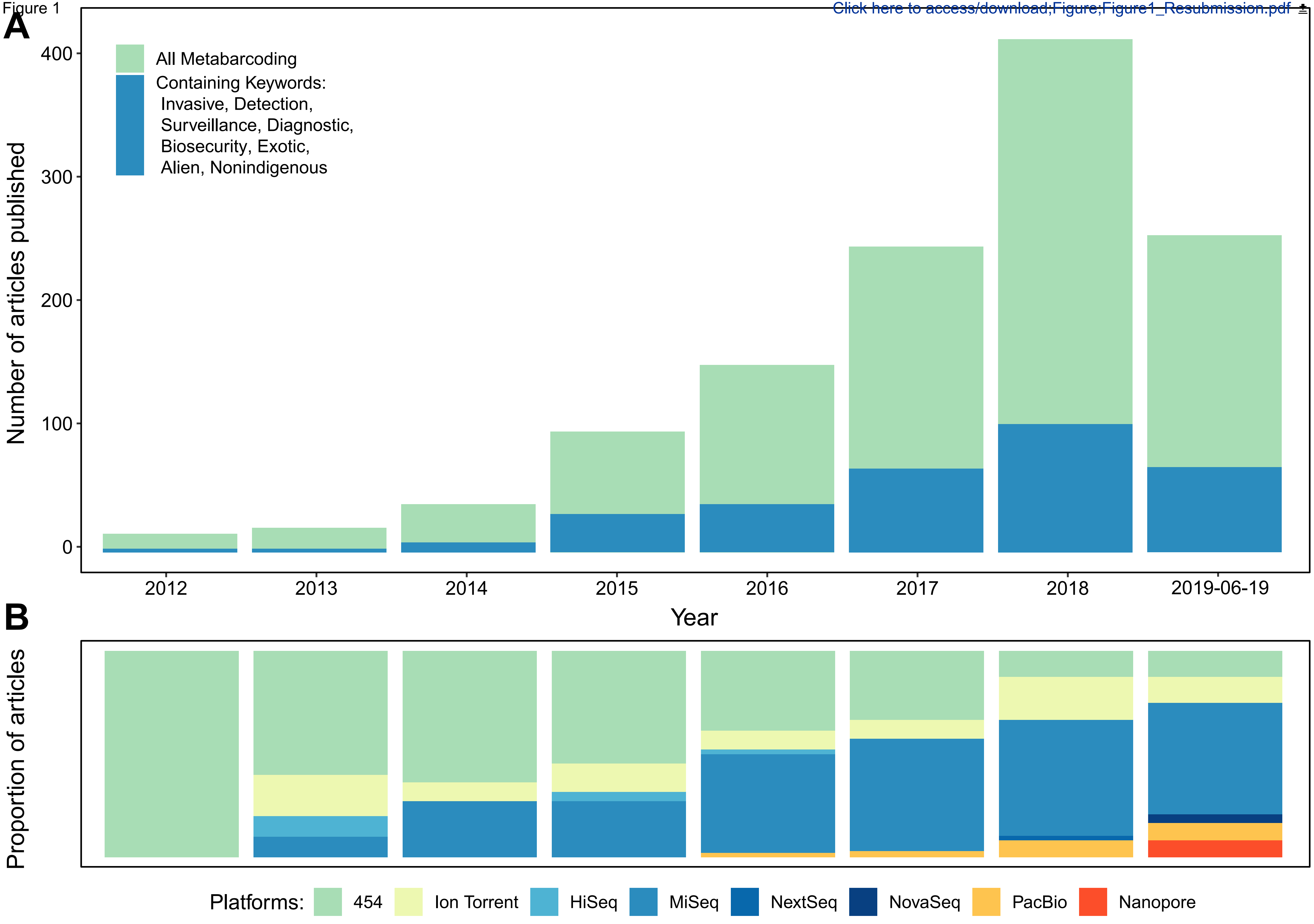

Figure 2

Click here to access/download;Figure;Figure2_Resubmisison.pdf

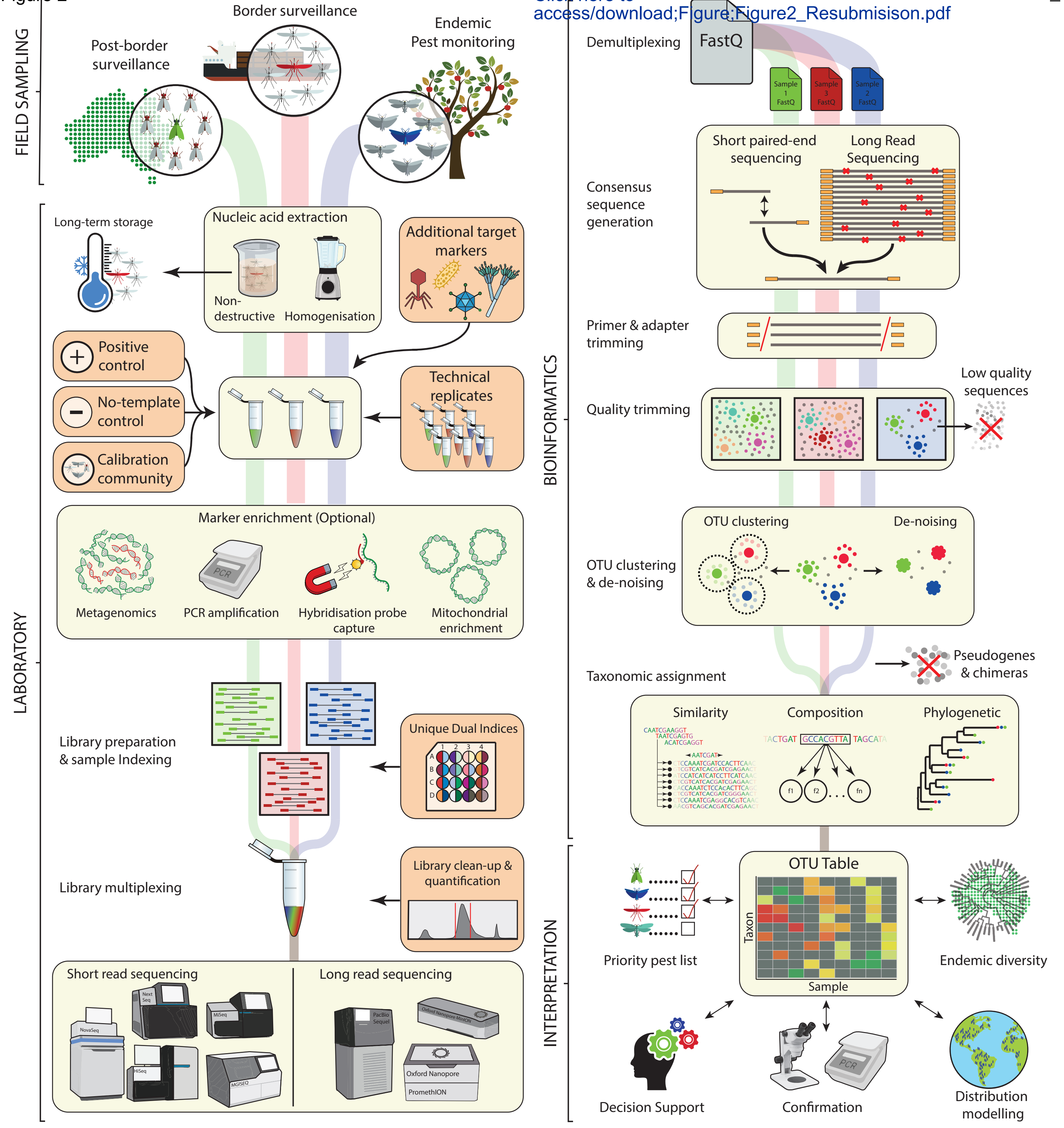

Figure 3

Click here to access/download;Figure;Figure3_Resubmission.pdf

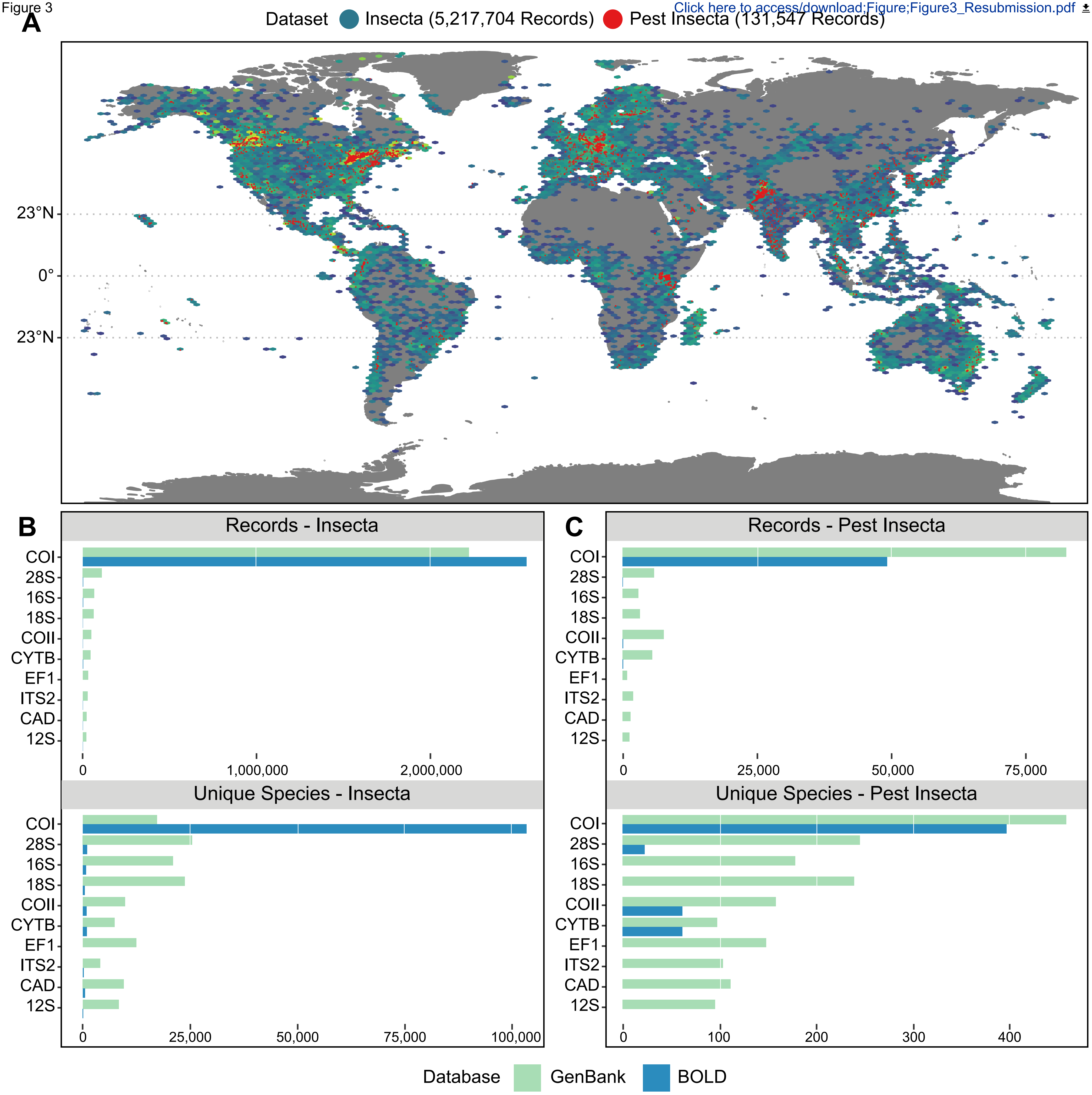

Figure 4

Click here to access/download;Figure;Figure4_Resubmission.p

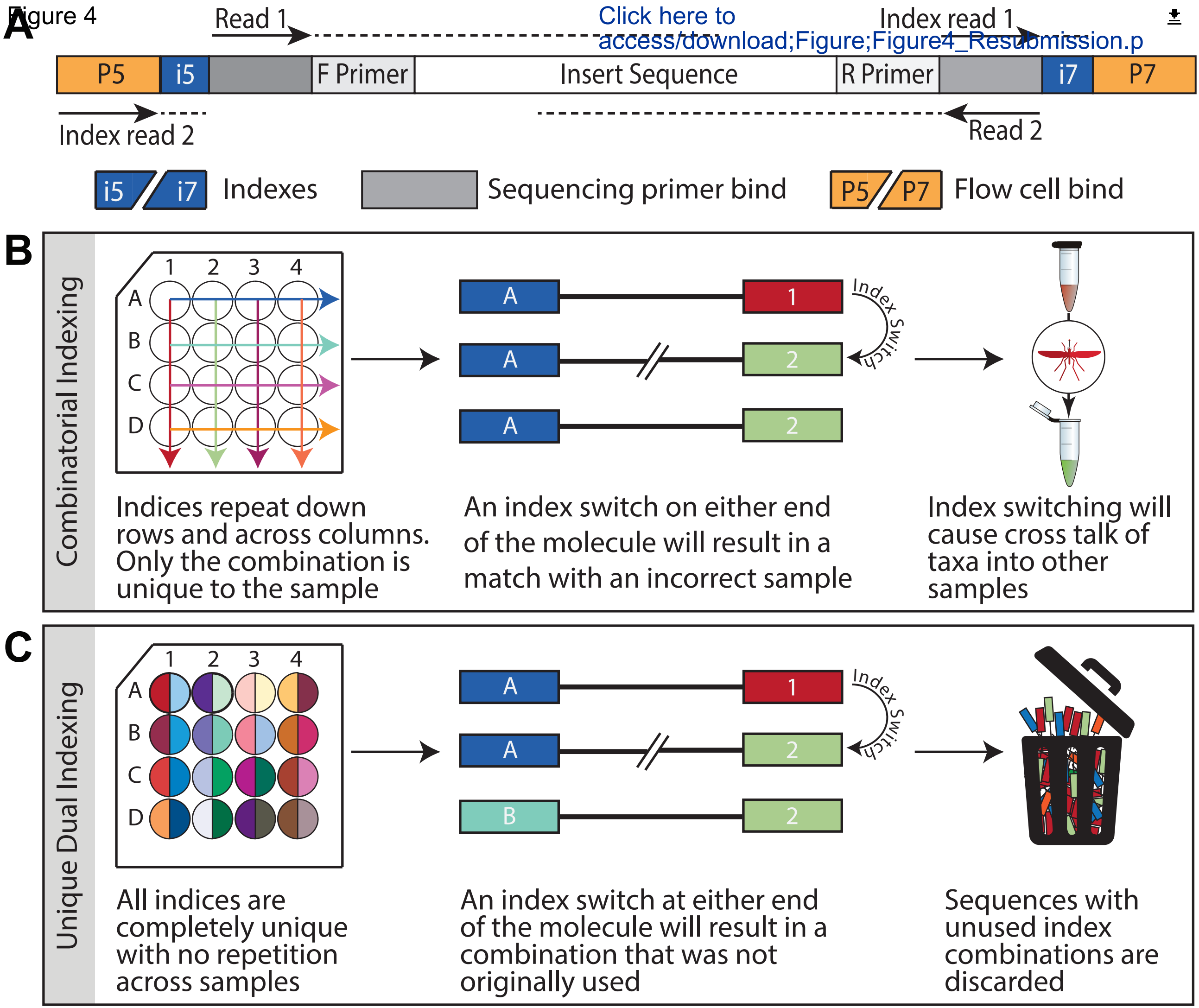

Supplementary S1

Click here to access/download
Supplementary Material
Supplementary_S1_Resubmission.pdf
