# Supplementary material for: Prospects and challenges of implementing DNA metabarcoding for high-throughput insect surveillance
Source: Gigascience. 2019 Jul 30;8(8):giz092. doi: 10.1093/gigascience/giz092 (PMC6667344; doi:10.1093/gigascience/giz092)
Supplement: giz092_Supplemental_File [file giz092_supplemental_file.pdf]

Prospects and challenges of implementing DNA metabarcoding for high-throughput insect surveillance

Supplementary Information 1

A.M. Piper, J. Batovska, N.O.I. Cogan, J. Weiss, J.P Cunningham, B.C. Rodoni, M.J. Blacket

2019/06/19

Introduction

This RMarkdown document contains the reproducible workflow that performed the analyses presented for the manuscript *Prospects and Challenges of implementing DNA metabarcoding for High-Throughput Surveillance of Trapped insects* by Alexander M. Piper, Jana Batovska, Noel O.I. Cogan, John Weiss, John Paul Cunningham, Brendan C. Rodoni and Mark J. Blacket

Setup workspace and load required packages

```
##       rentrez          bold        seqinr       taxize      biofiles
##          TRUE          TRUE          TRUE         TRUE          TRUE
##     tidyverse        scales     gridExtra         grid         readr
##          TRUE          TRUE          TRUE         TRUE          TRUE
##      fulltext    data.table        ggpubr      rscopus tidystringdist
##         FALSE          TRUE          TRUE         TRUE          TRUE
```

Literature search for all metabarcoding studies contained within the Scopus, PubMed and Crossref databases

Here we use the rscopus, rentrez and fulltext packages to retrieve all metabarcoding articles. These searches require the use of relevant databse APIs, which will require registration. Once registered, these APIs can be saved into your .Renviron by running usethis::edit_r_environ() and restarting R

- ENTREZ_KEY=" - Register at https://www.ncbi.nlm.nih.gov/account/
- ELSEVIER_SCOPUS_KEY=" - Register at https://dev.elsevier.com/index.html
- SPRINGER_KEY=" - Register at https://dev.springer.com/
- crossref_email=" - Add an email adress for faster queries

```
#Scopus search
Scopus_search <- rscopus::scopus_search("TITLE-ABS-KEY ( Metabarcod* ) ",
                                        count = 25, verbose = FALSE,
  max_count = 20000,view="COMPLETE")
scopus <- rscopus::gen_entries_to_df(Scopus_search$entries)
scopus <- scopus$df %>%
  dplyr::filter(`prism:aggregationType` == "Journal")%>%
  dplyr::mutate(YearPub = lubridate::year(readr::parse_date(`prism:coverDate`,
                                                    format="%Y-%m-%d")))
colnames(scopus) <- str_replace_all(colnames(scopus),
                                    pattern="prism:|dc:",replacement="")
write_csv(scopus, path=paste0("data/fig1/scopus_",Sys.Date(),".csv"))

#Pubmed Search
pubmed_search <- entrez_search(db="pubmed", term="Metabarcod*",use_history = TRUE)
```

```r
pubmed_fetch <-  entrez_fetch(db="pubmed", web_history=pubmed_search$web_history,
                         rettype="null", retmode="xml",retmax=10000)
xml <- parse_pubmed_xml(pubmed_fetch)

data <- list()
for (i in 1:length(xml)){
  if(!identical(xml[[i]][["abstract"]], list()) &&
     !identical(xml[[i]][["year"]], list())  ){
  row <- tibble(title=xml[[i]][["title"]],
                YearPub=xml[[i]][["year"]],
                pmid=xml[[i]][["pmid"]],
                abstract=xml[[i]][["abstract"]])
  data[[i]] <- row
  } else NULL
}
entrez <- bind_rows(data) %>%
  mutate(YearPub = as.numeric(YearPub))
write_csv(entrez, path=paste0("data/fig1/entrez_",Sys.Date(),".csv"))

#Crossref Search
crossref <- ft_search(query="Metabarcoding", from="crossref",limit=1000)

cross <- search$crossref$data %>%
  dplyr::mutate(YearPub = lubridate::year(readr::parse_date(created,
                                                            format="%Y-%m-%d"))) %>%
  mutate(author = vapply(test$author, paste, collapse = ", ", character(1L))) %>%
  mutate(funder = vapply(test$funder, paste, collapse = ", ", character(1L))) %>%
  mutate(reference = vapply(test$reference, paste, collapse = ", ", character(1L))) %>%
  select(-c(license,link,assertion))
write_csv(cross, path=paste0("data/fig1/crossref_",Sys.Date(),".csv"))


#Merge all citations & Filter

crossref <- read_csv(file=paste0("data/fig1/crossref_",Sys.Date(),".csv"))
entrez <- read_csv(file=paste0("data/fig1/entrez_",Sys.Date(),".csv"))
scopus <- read_csv(file=paste0("data/fig1/scopus_",Sys.Date(),".csv")) %>%
  rename(abstract = description)

df_all <- dplyr::bind_rows(crossref,entrez,scopus) %>%
  dplyr::mutate(title = stringr::str_to_lower(title) %>%
                  stringr::str_replace(pattern="\\.",replacement="")%>%
                  stringr::str_replace(pattern="  ",replacement=" ") %>%
                  stringr::str_replace(pattern="<inf>",replacement="") %>%
                  stringr::str_replace(pattern="</inf>",replacement="") %>%
                  stringr::str_replace(pattern="<sup>",replacement= "") %>%
                  stringr::str_replace(pattern="</sup>",replacement= "") %>%
                  stringr::str_replace_all("[^[:alnum:]]", " ") %>% # Remove special char
                  stringr::str_replace(pattern="  ",replacement= " ") %>%
                  stringr::str_trim(side="both") %>%
                  stringr::str_squish()
                ) %>%
  dplyr::filter(!str_detect(title, pattern="erratum")) %>%
```

```
  dplyr::select(doi,title,abstract,YearPub) %>%
  filter(!is.na(abstract)) %>% # remove all without abstracts - duplicated records
  dplyr::distinct(title, .keep_all=TRUE)

#Filter any further duplicates using fuzzy string matching
fuzzy <- expand.grid(df_all$title, df_all$title)%>%
  rename(V1 = Var1, V2 = Var2) %>%
  tidy_stringdist(.,method="lv") %>%
  filter(lv > 0) %>%
  filter(lv < 10)

df_all <- df_all %>%
  dplyr::filter(!title %in% as.character(fuzzy$V1)) %>%
  mutate(abstract = str_to_lower(abstract))

#Save filtered citations
write_csv(df_all, path=paste0("data/fig1/merged_citations_",Sys.Date(),".csv"))
```

Keyword processing of retrieved articles

The keywords used are:

Figure 1A - Invasive species keywords + invasive + detection + surveillance + diagnostic + alien + nonindigenous + non indigenous + biosecurity + exotic

Figure 1B - Sequencing platform keywords + 454 OR pyroseq + hiseq + miseq + nextseq + novaseq + pacbio OR pacific biosciences + mgiseq + ion torrent + nanopore OR minion OR promethion

```
#Fig 1A
#Load filtered citations
df_all <- read_csv(file=paste0("data/fig1/merged_citations_2019-06-19.csv"))

#Define count keywords function
count_keywords <- function(x,keywords){
  title <- x %>%   dplyr::filter(str_detect(x$title,
                         pattern=paste(!!keywords,collapse="|")))
  abs <- x %>%   dplyr::filter(str_detect(x$abstract,
                               pattern=paste(!!keywords,collapse="|")))
  out <- bind_rows(title,abs) %>%
            dplyr::group_by(YearPub) %>%
              dplyr::count()
return(out)
}

#Count all Metabarcoding articles
all_count <- df_all %>% group_by(YearPub) %>%
              dplyr::count()%>%
  rename(Metabarcod = n)

#Count invasive species related Metabarcoding articles
inv_count <- count_keywords(df_all,keywords=c("invasive","detection",
                                    "surveillance","diagnostic","alien",
                                    "nonindigenous","non indigenous",
                                    "biosecurity","exotic")) %>%
  rename(invasive = n)
```

```
#Fig 1B

#Count all articles using 454 sequencing
search_454 <- count_keywords(df_all,keywords=c("454","pyroseq")) %>%
        rename(`454` = n)

#Count all articles using HiSeq sequencing
search_hiseq <- count_keywords(df_all,keywords="hiseq") %>%
        rename(HiSeq = n)

#Count all articles using MiSeq sequencing
search_miseq <-  count_keywords(df_all,keywords="miseq") %>%
        rename(MiSeq = n)

#Count all articles using NextSeq sequencing
search_nextseq <-  count_keywords(df_all,keywords="nextseq")%>%
        rename(NextSeq = n)

#Count all articles using NovaSeq sequencing
search_novaseq <- count_keywords(df_all,keywords="novaseq") %>%
        rename(NovaSeq = n)

#Count all articles using PacBio sequencing
search_pacbio <- count_keywords(df_all,keywords=c("pacbio","pacific biosciences")) %>%
        rename(PacBio = n)

#Count all articles using MGI sequencing

search_mgi <- count_keywords(df_all,keywords="mgiseq") %>%
        rename(MGI = n)
#NOTE: No articles were found

#Count all articles using Ion Torrent sequencing
search_ion <- count_keywords(df_all,keywords="ion torrent") %>%
        rename(`Ion Torrent` = n)

#Count all articles using Nanopore sequencing
search_nano <- count_keywords(df_all,keywords=c("nanopore","minion","promethion")) %>%
        rename(Nanopore = n)

#Merge together citations for figure 1a and write out CSV
df_1a <- full_join(all_count,inv_count,by="YearPub") %>%
  dplyr::filter(YearPub > 2011) %>% # filter those prior to 2012
  gather(key="Search",value="Papers",-YearPub)
write_csv(df_1a, path="data/fig1/litsearch_1a.csv")

#Merge together citations for figure 1b and write out CSV
df_1b <- left_join(search_454, search_hiseq, by="YearPub") %>%
                left_join(., search_miseq, by="YearPub") %>%
                left_join(., search_nextseq, by="YearPub") %>%
                left_join(., search_novaseq, by="YearPub") %>%
                left_join(., search_pacbio, by="YearPub") %>%
                left_join(., search_ion, by="YearPub") %>%
```

```r
                left_join(., search_nano, by="YearPub") %>%
  dplyr::filter(YearPub > 2011) %>% # filter those prior to 2012
  gather(key="Search",value="Papers",-YearPub)
write_csv(df_1b, path="data/fig1/litsearch_1b.csv")
```

Figure 1 plotting

- Figure 1a - Published for for all metabarcoding studies, and those containing keywords in title or abstract relevant to invasive insect surveillance
- Figure 1b - Sequencing platforms used in the above metabarcoding studies displayed as a proportion for each year.

The csv files for fig 1a and fig1b are read back into R and plotted using ggplot. ggarrange from the package ggpubr is then used to produce the Fig 1 multiplot

```r
#Read in data
df_1a <- read_csv("data/fig1/litsearch_1a.csv") %>%
  mutate(Search = str_replace(Search, "invasive", "Containing Keywords: \n
                              Invasive, Detection, Surveillance
                              \n Diagnostic, Alien, Nonindigenous,
                              \n Biosecurity Exotic") %>%
         str_replace("Metabarcod", "All Metabarcoding"))
df_1b <- read_csv("data/fig1/litsearch_1b.csv") %>%
  mutate(Search = forcats::fct_relevel(Search, levels = c("454","Ion Torrent",
                                                          "HiSeq", "MiSeq",
                                                          "NextSeq","NovaSeq",
                                                          "PacBio","Nanopore")))

#Make Figure 1a
p1 <-ggplot(data=df_1a, aes(x=YearPub, y=Papers, fill=Search)) +
  geom_bar(stat="identity",position ="identity") +
  scale_x_discrete(limits=(2012:2019),breaks=(2012:2019)) +
  theme_pubr() +
  scale_fill_manual(values=c("#a8ddb5","#2b8cbe"),
  guide=guide_legend(nrow=2,title="Papers:")) +
  theme(legend.position = c(0.2,0.85), legend.direction = "vertical") +
  labs(x = "Year", y="Number of articles published")

#Make Figure 1b

#colours
p2 <- ggplot(df_1b, aes(fill=Search, y=Papers, x=YearPub)) +
        geom_bar( stat="identity", position="fill") +
        theme_pubr() +
        scale_fill_manual(values=c("#a8ddb5","#edf8b1","#4eb3d3",
                                   "#2b8cbe","#0868ac","#084081",
                                   "#fec44f","#fc4e2a","#9e9ac8"),
                          guide=guide_legend(nrow=1,title="Platform")) +
        theme(legend.position = "bottom")+
        theme(axis.text.y=element_blank(),
            axis.title.x=element_blank(),
            axis.text.x=element_blank(),
            axis.ticks.x=element_blank(),
            axis.ticks.y=element_blank())+
```

```
       ylab("Proportion of articles")

#Make multiplot

Fig1 <- ggarrange(p1, p2,
          ncol = 1, nrow = 2, labels = c("A","B"),
          heights = c(4, 2))
```

Figure 2 - Overview of common metabarcoding workflows for identification of trapped insect species

Figure 2 is an overview of common metabarcoding workflows revealed by a literature search. The figure itself was made entirely in Adobe Illustrator.

Figure 3- DNA barcodes on public reference databases

This figure compares the taxanomic, geographic, and dna barcode loci coverage of all insecta, and priority pest insects over NCBI GenBank and BOLD. While GenBank hosts greater overall sequence data, BOLD represents a curated database of loci used for DNA barcoding. Therefore, for direct comparison we will only use the loci contained on bold to query both databases.

Fetch bold data for Insecta

As the BOLD r package does not accept batch queries, instead the taxonomic names of all insect families present on bold were curated into a file called bold_insecta_families.txt and this file was then used to query the bold api for public records with sequences.

These files were then merged and simplified to only contain species_name,lattitude, longitude, collection country and marker

```
#Read in taxa list
taxon <- readLines(con = "bold_insecta_families.txt")

dir.create("data/fig3/bold")
possibleError <- 1 #create error object in advance

#Loop over taxa
for (k in 1:length(taxon)){
  time <- Sys.time() # get time

  #Download specimen and sequence data
  data <- tryCatch(bold_seqspec(taxon =taxon[k]),
    error=function(e)
      if(inherits(possibleError, "error")) next
  )

  possibleError <- tryCatch( if(length(data)!=0){
        # delete old file
    cat(file=paste0("output/bold/",taxon[k], "_", date,"_BOLD.csv"))
    #Write out header
    write.table(data[1,], file=paste0("output/bold/",taxon[k], "_", date,"_BOLD.csv"),
                append=T, sep="," , row.names = FALSE)
    #Write out data
    for (i in 1:nrow(data)){
      write.table(data[i,], file=paste0("output/bold/",taxon[k], "_", date,"_BOLD.csv"),
```

```r
                  append=T, sep=",", row.names = FALSE, col.names = FALSE)
    }
  } ,
  error=function(e)
    if(inherits(possibleError, "error")) next

  )
  time <- Sys.time() - time
  message(paste("Downloaded ", nrow(data)," sequences and specimen information for ",
                taxon[k], " in ", format(time, digits=2), " from BOLD.", sep=""))
}

##Read in all BOLD files, merge and subset them

bold_path <- "data/fig3/bold"
bold_dl <- sort(list.files(bold_path, pattern=".csv", full.names = TRUE))
length(bold_dl)

l = 1
possibleError <- 1 ##create error object in advance
datalist <- list()

#Create progress bar
pb <- txtProgressBar(min = 0, max = length(bold_dl), style = 3)

#loop over downloaded CSV's
for (l in 1:length(bold_dl)){
  time <- Sys.time()
  possibleError <- tryCatch( if (file.size(bold_dl[l]) > 0){

    #Read in bold_specimen CSV
    data <- read.csv(bold_dl[l], na.strings = c("","NA"))
    prefilt <- nrow(data)
    name <- bold_dl[l] %>%
      str_split_fixed("_", n=2)
    name <- name[[1]] %>%
      str_split_fixed("/", n=2)

    #Subset to necessary rows & filter incomplete genus species binomials
    data <- subset(data, dplyr::select=c("species_name",
                  "lat","lon","country","markercode")) %>%
     dplyr::filter(!grepl("sp.", species_name))
    datalist[[l]] <- data

    time <- Sys.time() - time
    },
    error=function(e) {warning(paste("Error, in file :", bold_dl[l]))},
    if(inherits(possibleError, "Error - Empty file")) next)


  # update progress bar
  setTxtProgressBar(pb, l)
  Sys.sleep(0.1)
```

```
}
close(pb)

# Collapse features into table
big_data <- rbindlist(datalist)

#write out csv
write_csv(big_data, path="data/fig3/bold/Insecta_bold_subset.csv")
```

Produce gene list

The bold data for insecta was was then summarised to produce a list of barcode contained on bold and gene names were then manually curated to be match gene names on with genbank

```
big_data <- read.csv(file="Arthropoda_bold_subset.csv")
bold_sum <- as.tibble(summary(big_data$markercode))


genelist <- as.character(unique(big_data$markercode))
writeLines(text=genelist, con = "genelist.txt", sep = "\n", useBytes = FALSE)
```

Fetch genbank data for Insecta

The curated list of all genes contaned on bold was then used to query genbank. As the rentrez package accepts batch queries, 'Insecta' was used instead of seperate families

These files were then processed to extract species names, GenBank accession, collection country, lattitude and logitude, and collection date

```
taxon <- "Insecta"
loci <- readLines(con = "curated_genelist.txt")
maxlength <- 2000
dir.create("data/fig3/genbank")

#Loop over loci
for (l in 1:length(loci)){
  dir.create(paste0("data/fig3/genbank/",loci[l]))

  #Loop over taxa
    for (k in 1:length(taxon)){
      searchQ <- paste("(",taxon[k], " [ORGN])", " AND (",
                        paste(c(loci[l]), collapse=" OR "), ") AND 1:",
                        maxlength ," [Sequence Length]", sep="")

        #Conduct entrez search
      search_results <- entrez_search(db   = "nuccore", term = searchQ,
                                       retmax=9999999, use_history=TRUE)

      if (search_results$count > 0){
        message(paste(search_results$count, taxon[k]," Sequences to be downloaded"))

        i <- 1
        start <- 0
        time <- Sys.time() # get time
```

```r
        #Split query into chunks
        chunks <- length(search_results$ids)/10000
        if (!is.integer(chunks)){chunks <- as.integer(length(search_results$ids)/10000)+1}

        #Loop over chunks
        for(i in i:chunks){

          destfile <- paste0("data/fig3/genbank/",loci[l],"/",taxon[k],"_",i,".gb")
          cat(file = destfile, sep="") # delete old file

          dl <- entrez_fetch(db="nuccore", web_history= search_results$web_history,
                             rettype="xml", retmode="gb", retmax=10000, retstart= start)

          cat(dl, file= destfile, sep=" ", append=T)
          message("Chunk", i, " of ",chunks, " downloaded\r")
          start <- start + 10000
          Sys.sleep(2.5)

          #Check if all chunks are downloaded
          if (i >= chunks){
            time <- Sys.time() - time
            message(paste("Download complete for: ", search_results$count,
                          " Sequences in ",  format(time, digits=2),
                          "From Genbank"))
          }
        }
      }  else {message(paste0("There are no ", loci[l],
                        " sequences available for ", taxon[k]))
        next
        }
    }
}

#Extract relevant data from genbank flat files using biofiles package

#Loop over loci
for (l in 1:length(loci)){

  # Read .gb filenames
  gb_path <- paste0("data/fig3/genbank/",loci[l])
  gb_files <- sort(list.files(gb_path, pattern=".gb", full.names = TRUE))
  message(paste(length(gb_files),"files to read for: ", loci[l]))

  #loop over files
  for (f in 1:length(gb_files)){
    message(paste("reading ", gb_files[f]))
      gb <- gbRecord(gb_files[f], progress = TRUE)

      #Extract features from record
      feat <- getFeatures(gb)
      featlist <- list()

      #Extract qualifiers (contains collection info) from features
```

```
      for (i in 1:length(feat)){
        line <- feat[[i]][[1]]@qualifiers
        line <- as.tibble(t(line))
        line$loci <- paste(loci[l], collapse="_")
        featlist[[i]] <- line
      }

      #Collase list
      featdata <- dplyr::bind_rows(featlist)

    #Add missing columns to incomplete files

      if (is.null(featdata$country)){
        featdata$country <- NA
      }
      if (is.null(featdata$lat_lon)){
        featdata$lat_lon <- NA
      }
      if (is.null(featdata$collection_date)){
        featdata$collection_date <- NA
      }

      #Subset features to only those necessary for figures
      sub_feat <- featdata %>%
                  dplyr::select(organism,loci,country,lat_lon,collection_date)

      #Write out table, appending as it goes

        write.table(sub_feat, file=paste0(gb_path,
                                          "/", paste(loci[l], collapse="_"),
                                          "_gb_apended_subset.csv"),
                                          append=T, sep="," , row.names = FALSE)

      message(paste0(nrow(sub_feat)," of ",length(gb),
                     " for ", gb_files[f], " processed"))
  }
}
```

Fetch genbank data for pest insects

A list of global priority insect pests list of global insect pests was assembled using the list from *Ashfaq M, Hebert PDN, Naaum A. DNA barcodes for bio-surveillance: Regulated and economically important arthropod plant pests. Genome. 2016;59:933–45* and combining it with additional pests of concern for Australia listed in *Plant Health Australia. The National Plant Biosecurity Status Report. 2017* This list was then filtered to retain only unique insect species with full Genus Species biniomials, leaving 558 taxa remaining.

This species list was then used alongside the previously curated gene list to download specimen data for pest insects from BOLD

```
#Read in pest species list
taxon <- readLines(con = "pest_list_insecta_only.txt")

dir.create("data/fig3/bold_pest")
possibleError <- 1 #create error object in advance
```

```
#Loop over taxa
for (k in 1:length(taxon)){
  time <- Sys.time() # get start time

  #Conduct search
  data <- tryCatch(bold_seqspec(taxon =taxon[k]),
    error=function(e)
      if(inherits(possibleError, "error")) next
  )

  possibleError <- tryCatch( if(length(data)!=0){

    # delete old file
    cat(file=paste0("data/fig3/bold/",taxon[k], "_", date,"_BOLD.csv"))
    # Write out header
    write.table(data[1,], file=paste0("data/fig3/bold_pest/",taxon[k], "_",
                                      date,"_BOLD.csv"), append=T,
                                      sep="," , row.names = FALSE)

    # Write out data
    for (i in 1:nrow(data)){
      write.table(data[i,], file=paste0("data/fig3/bold_pest/",taxon[k],
                                        "_", date,"_BOLD.csv"), append=T,
                                        sep=",", row.names = FALSE, col.names = FALSE)
    }
  } ,
  error=function(e)
    if(inherits(possibleError, "error")) next

  )
  time <- Sys.time() - time
  message(paste("Downloaded ", nrow(data),
                " sequences and specimen information for ", taxon[k],
                " in ", format(time, digits=2), " from BOLD.", sep=""))
}

#Read in all BOLD csv's, merge and subset them

bold_path <- "data/fig3/bold_pest"
bold_dl <- sort(list.files(bold_path, pattern=".csv", full.names = TRUE))
length(bold_dl)

l = 1
possibleError <- 1 #Create error object in advance
datalist <- list()

#Create progress bar
pb <- txtProgressBar(min = 0, max = length(bold_dl), style = 3)

#Loop over files
for (l in 1:length(bold_dl)){
  time <- Sys.time() # get time
  possibleError <- tryCatch( if (file.size(bold_dl[l]) > 0){
```

```
    #Read in bold_specimen CSV
    data <- read.csv(bold_dl[l], na.strings = c("","NA"))
    prefilt <- nrow(data)
    name <- bold_dl[l] %>%
      str_split_fixed("_", n=2)
    name <- name[[1]] %>%
      str_split_fixed("/", n=2)

    #Subset to necessary rows & filter incomplete genus species binomials
    data <- data %>%
      subset(select=c("species_name","lat","lon","country","markercode")) %>%
     dplyr::filter(!grepl("sp.", species_name))
    datalist[[l]] <- data

    time <- Sys.time() - time
    },
    error=function(e) {warning(paste("Error, in file :", bold_dl[l]))},
    if(inherits(possibleError, "Error - Empty file")) next)


  # update progress bar
  setTxtProgressBar(pb, l)
  Sys.sleep(0.1)
}
close(pb)

#Collapse list into table
big_data <- rbindlist(datalist)

#Write out csv
write.csv(big_data, file="data/fig3/bold_pest/Pest_bold_subset.csv")
```

Fetch genbank data for pests

The species list and the curated gene list were then used to download genbank data for pest insects

Genbank flat files were then parsed to obtain organism names,loci name, collection country, lattitude, longitude and collection date

```
#Read in taxon and loci lists
taxon <- readLines(con = "pest_list_insecta_only.txt")
loci <- readLines(con = "curated_genelist.txt")
maxlength <- 2000
dir.create("data/fig3/genbank_pest")

#fetch gb flatfiles from genbank

#Loop over loci
for (l in 1:length(loci)){
  dir.create(paste0("data/fig3/genbank_pest/",loci[l]))
    for (k in 1:length(taxon)){
      searchQ <- paste("(",taxon[k], " [ORGN])", " AND (",
                       paste(c(loci[l]), collapse=" OR "),") AND 1:",
                       maxlength ," [Sequence Length]", sep="")
```

```r
        #Conduct entrez search
      search_results <- entrez_search(db   = "nuccore", term = searchQ,
                                      retmax=9999999, use_history=TRUE)

      if (search_results$count > 0){
        message(paste(search_results$count, taxon[k]," Sequences to be downloaded"))

        i <- 1
        start <- 0
        time <- Sys.time() # get time

        #Split query into chunks
        chunks <- length(search_results$ids)/10000
        if (!is.integer(chunks)){chunks <- as.integer(length(search_results$ids)/10000)+1}

        #Loop over chunks
        for(i in i:chunks){
          destfile <- paste0("data/fig3/genbank_pest/",loci[l],"/",taxon[k],"_",i,".gb")
          cat(file = destfile, sep="") # delete old file

          #Downlaod specimen information
          dl <- entrez_fetch(db="nuccore", web_history= search_results$web_history,
                             rettype="xml", retmode="gb", retmax=10000, retstart= start)

          #Append out data
          cat(dl, file= destfile, sep=" ", append=T)
          message("Chunk", i, " of ",chunks, " downloaded\r")
          start <- start + 10000
          Sys.sleep(2.5)

          #Check if all chunks complete
          if (i >= chunks){
            time <- Sys.time() - time
            message(paste("Download complete for: ", search_results$count,
                          " Sequences in ",  format(time, digits=2), "From Genbank"))
          }
        }
      }  else {message(paste0("There are no ", loci[l],
              " sequences available for ", taxon[k]))
        next
        }
    }
}

#Extract relevant data using biofiles package

#Loop over loci
for (l in 1:length(loci)){
  gb_path <- paste0("data/fig3/genbank_pest/",loci[l])
  gb_files <- sort(list.files(gb_path, pattern=".gb", full.names = TRUE))

  message(paste(length(gb_files),"files to read for: ", loci[l]))
```

```
    gb <- gbRecord(gb_files, progress = TRUE)

    #Extract features from record
    feat <- getFeatures(gb)
    featlist <- list()

    #Extract qualifiers (contains collection info) from features
    for (i in 1:length(feat)){
      line <- feat[[i]][[1]]@qualifiers
      line <- as.tibble(t(line))
      line$loci <- paste(loci[l], collapse="_")
      featlist[[i]] <- line
    }

    #Collase list
    featdata <- dplyr::bind_rows(featlist)

  #Add missing columns to incomplete files

    if (is.null(featdata$country)){
      featdata$country <- NA
    }
    if (is.null(featdata$lat_lon)){
      featdata$lat_lon <- NA
    }
    if (is.null(featdata$collection_date)){
      featdata$collection_date <- NA
    }

    #Subset features to only those necessary for plotting
    sub_feat <- featdata %>%
                dplyr::select(organism,loci,country,lat_lon,collection_date)

    #Write out table, appending as we go
      write.table(sub_feat, file=paste0(gb_path,"/", paste(loci[l], collapse="_"),
                                        "_gb_apended_subset.csv"), append=T, sep="," ,
                                        row.names = FALSE)

    message(paste0(nrow(sub_feat)," of ",length(gb), " for ", loci[l], " processed"))
}
```

Merge datasets together and curate

BOLD and GenBank data for both the Insecta and Pest insect datasets were then merged, and gene names were curated where necessary to be compatible between datasets

```
#Read in GB Insecta and merge genes together
loci <- readLines(con = "curated_genelist.txt")
gblist <- list()

#Loop over loci
for (l in 1:length(loci)){
  gb_path <- paste0("data/fig3/genbank/",loci[l])
  gb_files <- sort(list.files(gb_path, pattern=".csv", full.names = TRUE))
```

```r
  gb <- read_csv(gb_files, na = c("","NA"))
  gb <- mutate_all(gb,as.character)
  gblist[[l]] <- gb
}

##Merge all rows & Write to file
gb_df <- dplyr::bind_rows(gblist)
write.csv(gb_df, file="data/fig3/genbank/all_genes_merged.csv")

#Read in GB Pest and merge genes together
loci <- readLines(con = "curated_genelist.txt")
gblist <- list()

#Loop over loci
for (l in 1:length(loci)){
  gb_path <- paste0("data/fig3/genbank_pest/",loci[l])
  gb_files <- sort(list.files(gb_path, pattern=".csv", full.names = TRUE))
  gb <- read_csv(gb_files, na = c("","NA"))
  gb <- mutate_all(gb,as.character)
  gblist[[l]] <- gb

}

##Merge all rows & Write to file
gb_pest <- dplyr::bind_rows(gblist)
write.csv(big_data, file="data/fig3/genbank_pest/all_genes_merged.csv")

#Merge both Pest and Insecta GB datasets together

gb_df <- read_csv("data/fig3/genbank/all_genes_merged.csv", na = c("","NA"))
gb_pest <- read_csv("data/fig3/genbank_pest/all_genes_merged.csv", na = c("","NA"))

gb_df$dataset <- "insecta"
gb_pest$dataset <- "pest"

gb_all <- dplyr::bind_rows(gb_df,gb_pest)

#Genbanks lattitude and longitude comes in NMEA format (ie N S E W).
#Needs to be converted to decimal ie (- +) and split into 2 collumnns

gb_all <- gb_all %>%
  separate(col=lat_lon, into=c("lat","latdir","lon","londir"),sep=" ")
gb_all$latdir <-  str_replace(gb_all$latdir, pattern="N",replacement="") %>%
  str_replace(pattern="S",replacement="-")
gb_all$londir <-  str_replace(gb_all$londir, pattern="E",replacement="") %>%
  str_replace(pattern="W",replacement="-")
gb_all$lat <- paste0(gb_all$latdir, gb_all$lat)
gb_all$lon <- paste0(gb_all$londir, gb_all$lon)
gb_all$lat <-  str_replace(gb_all$lat, pattern="NANA",replacement="")
gb_all$lon <-  str_replace(gb_all$lon, pattern="NANA",replacement="")

gb_all <- gb_all %>% dplyr::select(organism,lat,lon,country,loci,dataset)
gb_all$db <- "genbank"
```

```r
colnames(gb_all) <- c("species_name","lat","lon","country","loci","dataset","db")


#Read in and merge BOLD datasets for Pests and Insecta

bold_insecta <- read.csv("data/fig3/bold/Insecta_bold_subset.csv",
                         na.strings = c("","NA"))
bold_pest <- read.csv("data/fig3/bold_pest/Pest_bold_subset.csv",
                      na.strings = c("","NA"))

bold_insecta$dataset <- "insecta"
bold_pest$dataset <- "pest"

bold_all <- dplyr::bind_rows(bold_insecta,bold_pest)

bold_all <- bold_all %>%
  dplyr::select(species_name,lat,lon,country, markercode,dataset)
bold_all$db <- "bold"
colnames(bold_all) <- c("species_name","lat","lon","country","loci","dataset","db")

merged <- rbind(bold_all,gb_all)

#Rename genes to match between datasets and simplify closely related duplicates

merged$loci <-  merged$loci %>%
  str_replace(pattern="28S-D1-D2", replacement="28S") %>%
  str_replace(pattern="28S-D2", replacement="28S") %>%
  str_replace(pattern="28S-D2-D3", replacement="28S") %>%
  str_replace(pattern="28S-D3-D5", replacement="28S") %>%
  str_replace(pattern="COI-5P", replacement="COI") %>%
  str_replace(pattern="COI-3P", replacement="COI") %>%
  str_replace(pattern="COI OR COI OR COX1 OR COXI", replacement="COI") %>%
  str_replace(pattern="COXIII", replacement="COIII") %>%
  str_replace(pattern="COXIII OR COIII", replacement="COIII") %>%
  str_replace(pattern="COII OR COXII", replacement="COII") %>%
  str_replace(pattern="COXIII", replacement="COIII") %>%
  str_replace(pattern="COXIII", replacement="COIII")

#Remove all records with NA loci
merged <- merged[!is.na(merged$loci), ]

#Write out final dataset for plotting
write.csv(merged, file="data/fig3/merged_insecta_pest_bold_gb.csv")
```

Figure 3a - Global distribution of all sufficiently annotated DNA barcode records from BOLD and GenBank

All barcode records for every loci that contained lattitude and longitude information were then plotted on a world map

```r
#Read in data
merged <- read_csv("data/fig3/merged_insecta_pest_bold_gb.csv", na = c("","NA")) %>%
  mutate(lat = as.numeric(lat)) %>%
  mutate(lon = as.numeric(lon))
```

```
## Warning: Missing column names filled in: 'X1' [1]

## Warning: 235 parsing failures.
##     row col               expected actual                                      file
## 2762446 lat no trailing characters    '36 'data/fig3/merged_insecta_pest_bold_gb.csv'
## 2762446 lon no trailing characters    N,  'data/fig3/merged_insecta_pest_bold_gb.csv'
## 2762447 lat no trailing characters    '39 'data/fig3/merged_insecta_pest_bold_gb.csv'
## 2762447 lon no trailing characters    N,  'data/fig3/merged_insecta_pest_bold_gb.csv'
## 2936743 lat no trailing characters    ';3 'data/fig3/merged_insecta_pest_bold_gb.csv'
## ....... ... ...................... ...... ..........................................
## See problems(...) for more details.
```

```r
#Filter to only those records within possible lat lon range
map <- merged %>%
  dplyr::select(lat,lon,dataset,db) %>%
  na.omit() %>%
  dplyr::filter(!lat > 90) %>%
  dplyr::filter(!lat < -90) %>%
  dplyr::filter(!lon > 180) %>%
  dplyr::filter(!lat < -180)

#Count number of records post NA removal and latlon filter

print(paste0(nrow(merged)-nrow(map)," records containing NA's and",
             " outside real lat & Lon values removed"))
```

```
## [1] "985581 records containing NA's and outside real lat & Lon values removed"
```

```r
#Get world map polygons
wm <- map_data("world")

#Draw map and hexbin
p3 <- ggplot() +
  geom_polygon(data=wm, aes(x=long, y=lat, group=group),
               color="grey50", fill="grey50") +
      scale_y_discrete(limits=c(-23,0,23)) +
      geom_hex(data= map, bins=150, mapping=aes(x=lon, y=lat),
              inherit.aes = FALSE) + guides(shape=FALSE) +
  scale_fill_viridis_c(trans='log10',begin=0.2) +
        coord_equal() +
      theme_pubclean() +
      theme(axis.title.y=element_blank(),
            axis.text.y=element_blank(),
            axis.title.x=element_blank(),
            axis.text.x=element_blank(),
            axis.ticks.x=element_blank(),
            legend.position = "none")
plot(p3)
```

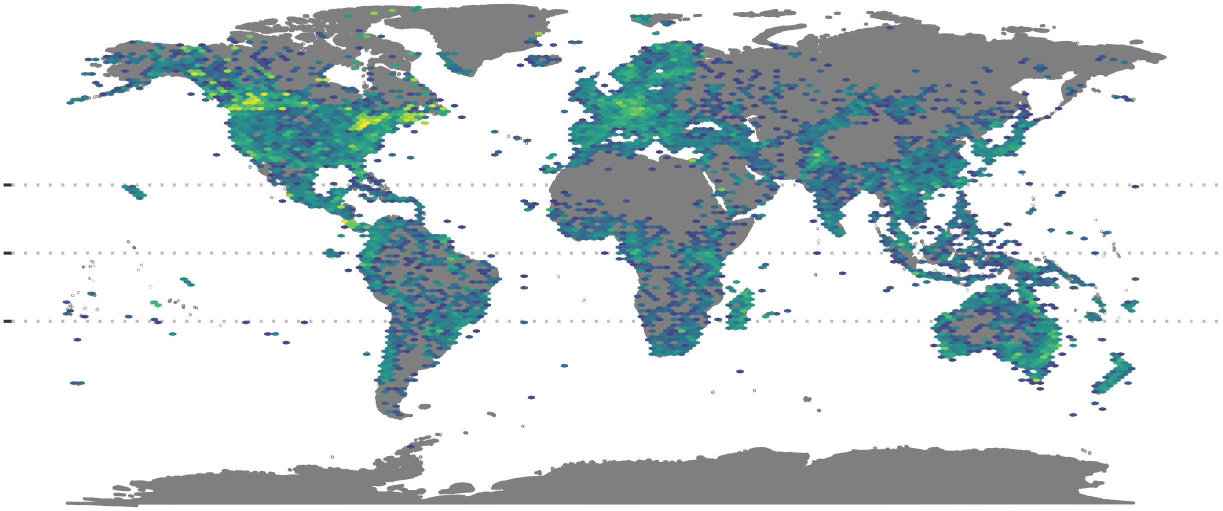

```
# Add invasive insect records to plot

p3 <- p3 + geom_point(data=map %>% dplyr::filter(dataset == 'pest'),
                 mapping=aes(x=lon, y=lat, shape="."),
                 color="#e31a1c", size=0.2,alpha=0.5) + guides(shape=FALSE)
plot(p3)
```

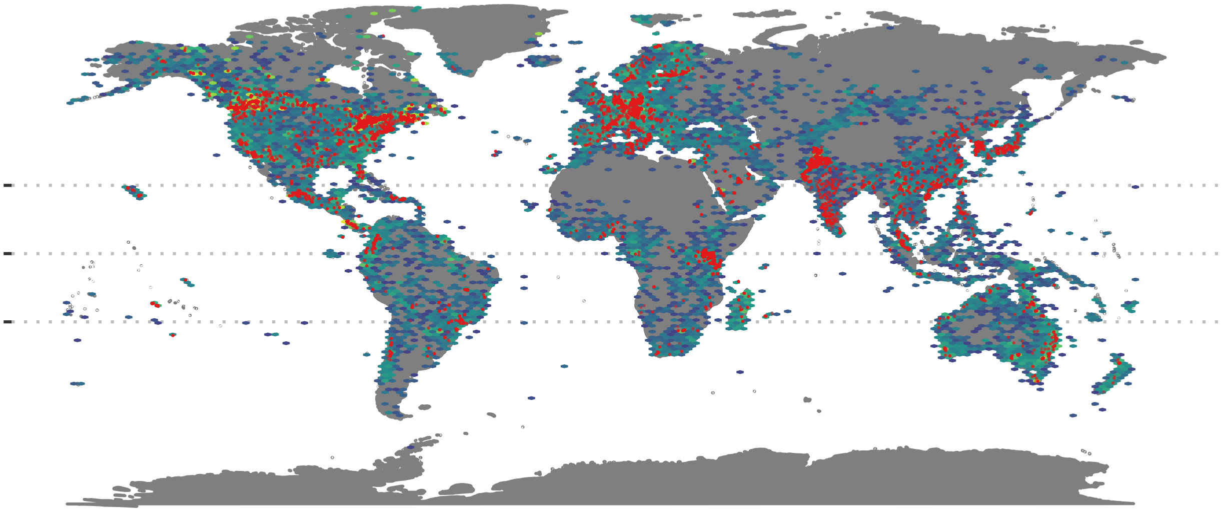

Figure 3B Distribution of records and 3C species within major public databases

The occurance of records and genes for the 10 barcode markers with the most reference information in both datasets were then plotted. Despite the PER gene appearing in this top 10, closer inspection revealed that this was due to many records studies on clock genes not DNA barcoding and therefore this gene was excluded from the plot.

```
#Read in data & remove PER from dataset
merged <- read_csv(file="data/fig3/merged_insecta_pest_bold_gb.csv") %>%
  dplyr::filter(!str_detect("PER",loci))

##Grab top 10 occuring genes ony
top10 <- merged %>%
  dplyr::count(loci) %>%
  top_n(10) %>%
```

```r
  arrange(n, loci) %>%
  mutate(loci = factor(loci, levels = unique(loci)))

top10 <- merged %>%
  dplyr::filter(loci %in% top10$loci) %>%
  mutate(loci = factor(loci, levels = levels(top10$loci)))

##Count unique species

spp_count <- top10 %>%
      dplyr::filter(!grepl("sp.", species_name, ignore.case=TRUE)) %>%
      dplyr::filter(!grepl("cf.", species_name, ignore.case=TRUE)) %>%
      dplyr::filter(!grepl("nr.", species_name, ignore.case=TRUE)) %>%
      group_by(loci, db, dataset) %>%
      summarise(count = n_distinct(species_name))
colnames(spp_count) <- c("loci","db","dataset","species")

##Count records
rec_count <- merged %>%
  group_by(loci, db,dataset) %>%
      dplyr::count(loci)
colnames(rec_count) <- c("loci","db","dataset","records")

#Merge Species and records count data, create column to Facet plots by

all_count <- merge(spp_count, rec_count) %>%
    gather(type, count, -loci, -dataset, -db) %>%
  mutate(set = factor(paste0(dataset,"_",type),
                      levels=c('insecta_records','pest_records',
                               'insecta_species','pest_species')))

#Plot figure 3B
p4 <- ggplot(data=all_count,
             aes(x=loci, y=count,group=db,fill=db)) +
    geom_bar(position="dodge",stat="identity",alpha=1) +
    theme_pubr() +
    scale_fill_manual(values=c("#2b8cbe","#a8ddb5"))+
    coord_flip() + facet_wrap(~set, scales="free") +
    theme(legend.position = "bottom")

#All records
print(paste0(sum((all_count %>% dplyr::filter(set == "insecta_records"))$count),
             " total records for Insecta"))
```

```
## [1] "5217704 total records for Insecta"
```

```r
#Pest records
print(paste0(sum((all_count %>% dplyr::filter(set == "pest_records"))$count),
             " total records for Pest Insecta"))
```

```
## [1] "131547 total records for Pest Insecta"
```

```r
plot(p4)
```

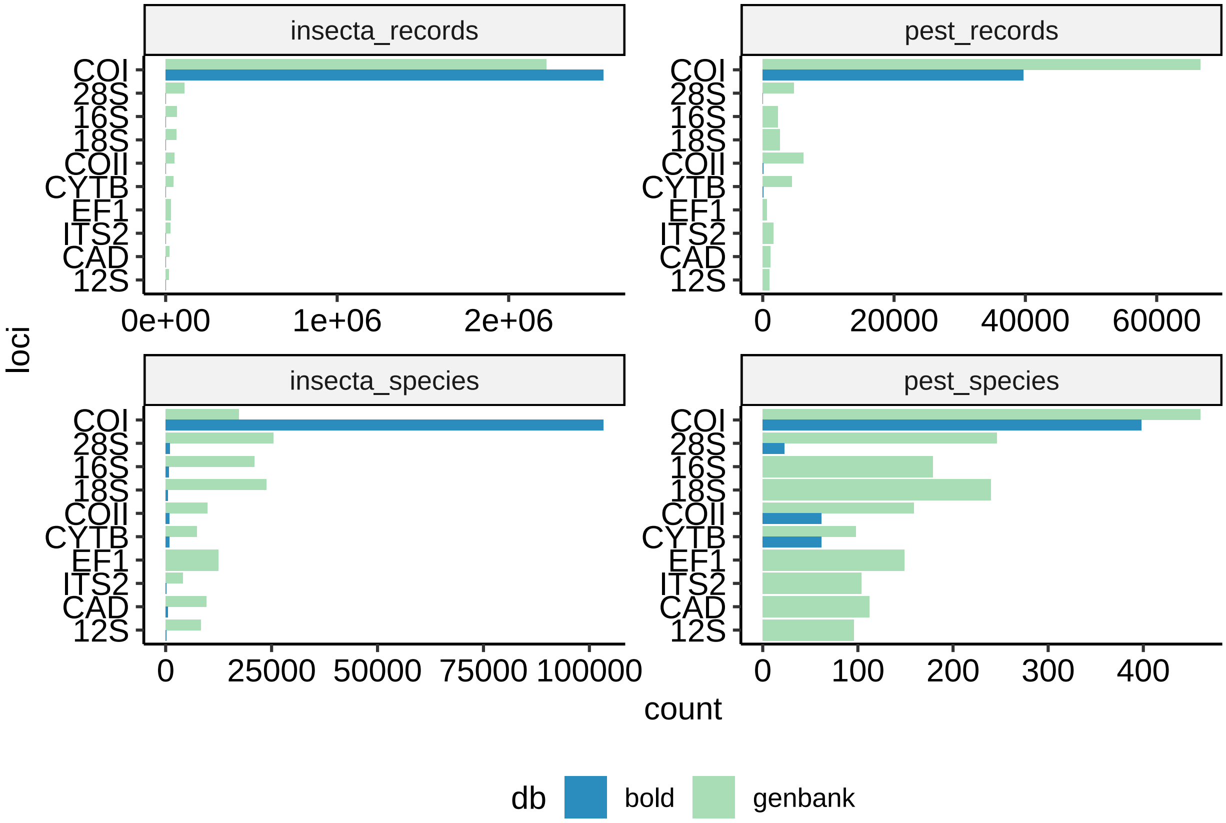

Plot final figure 3

The two sub figures were combined, then further edited in Adobe Illustrator

```r
Fig3 <- ggarrange(p3, p4,
          ncol = 1, nrow = 2, labels = c("A","B"),
          heights = c(7, 4), widths= c(2,1))
```

Figure 4 - Unique dual indexing overcomes issues of cross-contamination due to index switching

Figure 4 is an overview of common metabarcoding workflows revealed by a literature search. The figure itself was made entirely in Adobe Illustrator.
