# Supplementary material for: Prospects and challenges of implementing DNA metabarcoding for high-throughput insect surveillance
Source: Gigascience. 2019 Jul 30;8(8):giz092. doi: 10.1093/gigascience/giz092 (PMC6667344; doi:10.1093/gigascience/giz092)
Supplement: giz092_Response_to_Reviewer_Comments_Original_Submission [file giz092_response_to_reviewer_comments_original_submission.pdf]

Author's Response To Reviewer Comments

Close

Dear Editor,
We thank the reviewers for their constructive and helpful comments. Since our previous submission our manuscript has been updated to incorporate the reviewers' suggestions, the particulars of which we have listed below:

• We have expanded the breadth of our literature search, including further references uncovered through this process and those suggested by the reviewers.
• We have updated Figure 1 to reflect this expanded search, and it now includes citations from Crossref and Pubmed in addition to Scopus.
• We have condensed the discussion of alternative loci, as well as PCR free approaches and instead refer to in-depth reviews on the topics.
• We have added discussion of the limitations of multi-locus approaches in relation to assigning taxonomy and added an additional box that covers the use of multi-locus approaches for capturing markers beyond taxonomic inference, for example the parallel identification of vectored pathogens in modular metabarcoding assays.
• We have further covered the MGI/BGI sequencers in our discussion of sequencing platforms and Table 2 as per your suggestion and also have covered the recently released PacBio Sequel II.
• We have added additional detail in the Background and throughout the manuscript on the implications of barcoding for biosecurity law
• We have revised the taxonomic assignment and technical replicates sections to address important advances highlighted by the reviewers.
• Finally, we have updated Figure 2 to summarise our vision for how metabarcoding can be applied to insect pest surveillance and to include further aspects discussed in this manuscript such as technical replicates, controls, long read sequencing, additional loci, and interpretation of results.
We believe these changes address the primary concerns of the reviewers. Please find detailed responses to the specific comments of the reviewers below.

Reviewer #1: Piper et al. present a thorough review on DNA metabarcoding applied to high throughput insect surveillance. The text is well written and it does not contain any major flaw, so I think it is a good contribution to the literature. The authors should nevertheless be aware that similar reviews -although not dedicated to insect surveillance- already exist in the bibliography (two recent examples that the authors omitted: Deiner et al. 2017 Mol Ecol; Alberdi et al. 2019 Mol Ecol Res). These reviews cover some redundant issues, which could enable reducing the length of this article in some sections, but also contain some contents and ideas that have not been added to this article and might be worth referencing in the context of high throughput insect surveillance (e.g. using replicated restrictively to gain confidence on the identified taxa and reduce the risk of false positives).

Response: We have highlighted other reviews of the wider metabarcoding literature in the background, including the suggested references. In the quality assurance section of the manuscript we have further expanded on some of the ideas suggested in these references, including the benefits of additive or restrictive processing of replicates.

Reviewer #2: The manuscript is a review on the usage metabarcoding in the context of insect control, biosurveillance, and with a special emphasis on pests. It is an important contribution for control agencies around the world who require more throughput in their analysis of invasive species that can be carried in imports and movement of goods. It is also a good contribution to entomologists and molecular biologists looking to incorporate metabarcoding of these taxa into their research. The review includes mostly all steps involved in metabarcoding for surveillance purposes, from selecting a marker to quality assurance. In my opinion, the paper is presented in a coherent and organized manner. However, there are a few points that I consider will make the manuscript stronger, and that it would widen the reach of the paper. I would like to see a wider scope in their searching method (there are a few references missing that I

think will improve the manuscript). I also will like to see thorough discussion on the current state of affairs in international law about biosecurity and pest management , since this directly affect the applicability of barcodes in this context. Finally, the manuscript will benefit to have a figure perspectives and conclusions that summarize the author's vision on how to apply barcodes for bio-surveillance. Below more specific comments:

Response: We have expanded our literature search beyond the Scopus database and added further relevant references that were previously missed. We have added an additional paragraph to the introduction to address the current state of affairs in international biosecurity law since the introduction of the WTO agreement on Sanitary and Phytosanitary measures and provided additional detail throughout the manuscript where a particular aspect of the metabarcoding approach may be impacted by current regulatory frameworks. We agree that summarising our vision of how metabarcoding should be applied in a figure would benefit the manuscript. However, rather than adding an extra figure we have updated figure 2 to better summarise our vision for how samples a range of insect surveillance activities could be fed into the same core metabarcoding diagnostic assay, with the results informing relevant management actions. We have further updated this figure to include diagrams relating to quality control and long read sequencing.

* Typo in line 73
* Adjust Table2 column width
* In line 152 you make reference to figure 2 when you meant figure 3
* As before but in lines 167 and 168

Response: We thank the reviewer for bringing these mistakes to our attention and they have been corrected.

* It is not clear how can you obtain minimal amplification bias. This is not even clear in the cited articles, and one of them (ref. 83) even explicitly says that it requires in vitro validation and "as many primers might not be suitable for DNA metabardocing due to low base degeneracy, potentially high primer bias or critical design flaws". Therefore the sentences of lines 194-198 is a bit misleading and should be reworded, despite the following sentence introducing other sources of bias.

Response: We have reworded this to state: This bias is thought to mainly arise from primer-template mismatches, particularly at the 3' end of the primer where extension takes place (Piñol et al 2015, 2019) and therefore comprehensive in-silico evaluation should be conducted at the beginning of a project to ensure primer sequences are appropriate for the underlying target community (Rennstam Rubbmark et al 2018, Bylemans et al 2018 Ficetola et al 2010). Where mismatches with certain taxa are predicted to occur, inclusion of degenerate bases can overcome taxonomic bias inherent to a specific primer sequence (Elbrecht et al 2017), however high levels of degeneracy can also lead to undesirable off-target amplification or formation of dimers (Mioduchowska et al 2018, Marquina et al 2018) which will require further laboratory validation to detect (Clarke et al 2014, Elbrecht et al 2017)

* Despite the argument about avoiding PCR is compelling, I believe that the author's "over-endorsement" of the micro-array chips is a big leap. It is especially difficult to deliver the appropriate probes when good references are not present, as is one of the arguments of the paper.

Response: We have condensed this section and removed some of the discussion on microarrays and hybrid capture, instead referring to the in-depth reviews of Mamanova et al 2010 and Jones et al 2015 on the topic of PCR free targeted enrichment approaches. We have also raised a further consideration that implementation of PCR-free sequence enrichment may require overcoming further regulatory hurdles as opposed to the already wide acceptance of PCR amplification within diagnostic protocols.

* For an interesting discussion on index-switching (discussed around line 257), you can include https://doi.org/10.1111/1755-0998.12928

Response: We thank the reviewer for highlighting this paper, and we have added the reference to the manuscript

* Despite that the authors mentioned different sequencing platforms, they seem to restrict their discussion of "demultiplexing and sequence quality trimming" to Illumina pair end reads. They should include at least a paragraph regarding the rest of the platforms discussed

Response: In the interest of article length, rather than adding an additional paragraph we have adapted the current paragraph to be more generic to all the sequencers discussed. Overall the process is reasonably similar between Illumina and other modern platforms, with the exception being the need to assemble consensus sequences prior to quality trimming if using PacBio CCS or another third-party consensus method for Nanopore. We have also highlighted that quality trimming using error profiles is a coarse filtering process where parameters should be carefully considered, particularly for higher error rate nanopore reads. We have further added diagrams in figure 2 to help highlight the differences between analysis of metabarcoding data from short and long read platforms.

* I think that referencing 150 in line 374 sounds like Elbrecht et al. 2018 used zero-radius OTUs (ZOTU) to investigate intraspecific variation. It is a bit misleading since they used a 3% threshold after the ZOTU inference, essentially making it not an ESV. Some clarification would be needed.

Response: We have clarified that use of denoising algorithms (rather than ESVs per se) provide the ability to investigate intra-specific variation as they don't impose the arbitrary similarity threshold which define OTUs. This single nucleotide resolution enables binning sequences into 'amplicon sequence variants' that retain precise haplotype information that can be necessary for diagnostics of closely related taxa or tracking an invasion and act as a consistent label between analyses. We now reference Marshal & Stephien 2019 who use the fine-scale resolution provided by ASV's to infer population histories of an invasive species.

* Fix reference 167

Response: We thank the reviewer for noticing the error in reference 167 and it has been corrected.

* The authors should be careful with the statement " makes the classification process more robust to pervasive issues of missing and mis-annotated data in reference database". While is true that some ML implementations give some sort of confidence levels in the taxonomic hierarchy, it is not true that it would be less influenced by misannoations and missing references. In fact, most implementations of the Naive bayes will return NA when no significant match with the training set is found. Also, mislabels in the training set will lead to erroneous prediction (https://doi.org/10.1613/jair.606) and is an ongoing challenge in ML to detect mislabels (https://doi.org/10.1007/978-3-319-58628-1_43)

* The statement "In cases where there may be ambiguity due to imperfect reference data and multiple taxonomic outcomes obtain similar probabilities, the sequence may still be robustly assigned to a higher taxonomic rank (e.g. family) [76], providing important information about sample composition and possible presence of novel taxa" is also problematic in my view. The text suggest that Naive bayes by itself has this property, which is not true. Only specific implementations and variations of the algorithm have it. The paper would benefit from exploring this further.

Response to both of the above: We agree with the reviewer that this section of the manuscript unintentionally conflates the specifics of the RDP implementation of naïve Bayes algorithm with the broader concept of probabilistic approaches to sequence classification. We have now revised the taxonomic assignment section so that Paragraph 1 introduces the general approaches to sequence classification (Sequence similarity, sequence composition, phylogenetic, or a hybrid of these) and then refers to other reviews and comparisons of these methods. The remainder of paragraph 1 focuses on the problem of over-classification using the popular BLAST best-hit assignment method. Paragraph 2 now focuses more broadly on the importance of using methods of classification that return a confidence score, which is particularly important for metabarcoding applications where management decisions are based on appropriate assignment of a taxonomic name to sequence reads.

* Some important development is missing in the taxonomic assignment section. The probabilistic method Protax (DOI: 10.1093/bioinformatics/btw346,DOI: 10.1111/2041-210X.12721). Also Axtner et al. (doi: https://doi.org/10.1101/345082) preprint contains a very robust workflow for eDNA, and I think you can extract important information from their discussion. Protax do not require a complete database,

gives hierarchical probability of assignment and has a very elegant mathematical framework. It would be a shame if it is not included in your review (or at least mention it to the public).

Response: We agree with the reviewer that we have overlooked the importance of PROTAX method for sequence classification. In paragraph 2 of the taxonomic assignment section we now introduce the PROTAX method and discuss its advantages, in particular the ability to model probability that a sequence belongs to species that exist in Linnaean taxonomy but not in the sequence reference database, as well as modelling the probability that a sequence comes from a taxa novel to both the reference sequence database and reference taxonomy. In box 1 we further highlight the ability of PROTAX and other methods to set a prior weighting for certain sequences, which could be useful when combining high confidence in-house sequences with more variable quality public sequences, or when the endemic diversity for the target region is well characterized.

*I disagree with the authors when they say "While the importance of technical replication for increasing detection probability is generally agreed upon [31,178,179], replicates reduce throughput without providing further independent data points [26]. Instead, with metabarcoding removing one of the major roadblocks to large-scale surveillance, more biological replicates from more frequent and intensive trapping could be used. Considering biological replication is particularly important as regardless of the effectiveness of the metabarcoding diagnostic assay if an insect is not caught in a trap it does not necessarily mean absence in the area". While I agree on the importance of biological replication (and my argument is never against that), technical replicates are of utmost important, especially when dealing with rare species and invasive species. Even if the entire labwork is done in pristine condition, with close to 0 bias, the resulting sequences vary dramatically between replicates. Also, technical replicates inform you of the overall quality of your survey, and allows you to model or correct for biases that might have occur. I think it would be a disservice to the community to let a statement that disregards technical replicates pass. Please consider revising this point.

Response: We agree with the reviewer that we have discounted the importance of technical replication too far. This section has been revised and we have emphasised the importance of technical replication to counter stochasticity during PCR and library preparation and identify laboratory cross-contamination in the case that replicates show significant dissimilarities in taxonomic composition. We then contrast additive and restrictive processing of replicates, and suggest it may be best to include a minimum number of technical replicates to allow a majority rules approach (i.e. 2/3 replicates count as a detection) to balance the positives of both approaches. We then suggest the use of site occupancy models to determine the appropriate number of both biological and technical replicates required to reach the desired statistical power for the survey.

* In line 541, there is something missing after "similar"

Response: We have corrected this to read: “similar standardisation of metabarcoding protocols”

* While I liked and agree with the idea exposed between the lines 541 and 550, the references cited proposed in silico mock communities. Despite this is necessary, I think the authors should also push for the usage of a standard and diverse mock community. The analyses of in silico mock communities and the real mock communities have shown important differences between the two.

Response: We have suggested the use of standard and diverse mock communities, alongside computational datasets for benchmarking, and have given the example of the ZymoBIOMICS microbial mock community standards, which are used in the microbiome sequencing field to allow more realistic benchmarking of both laboratory and bioinformatics methods than can be provided by in-silico mock communities alone.

* Although Scopus is a large database, it missing a significant amount of citations. I would encourage the authors to widen their scope to other sources. R has a very good package called fulltext that encompasses multiple sources.

Response: The literature search has been expanded beyond Scopus and figure 1 has been recreated to also include citations listed on Crossref, and Pubmed, resulting in an addition of 16 citations for years prior to 2019. We have also included all 2019 publications to date, adding an additional 257 citations to

this figure and resulting in the use of Nanopore sequencing and Illumina NovaSeq now being represented in Fig1b. The methods section and Supplementary 1 have been updated to include the new methods involved in the creation of this figure.

We thank the reviewers for their insightful and constructive comments, which have helped us improve the manuscript.

Close
