# Supplementary material for: Prospects and challenges of implementing DNA metabarcoding for high-throughput insect surveillance
Source: Gigascience. 2019 Jul 30;8(8):giz092. doi: 10.1093/gigascience/giz092 (PMC6667344; doi:10.1093/gigascience/giz092)
Supplement: giz092_Reviewer_2_Report_Original_Submission — Jose Hleap -- 4/8/2019 Reviewed [file giz092_reviewer_2_report_original_submission.pdf]

Reviewer Report

Title: Prospects and challenges of implementing DNA metabarcoding for high throughput insect surveillance

Version: Original Submission **Date:** 4/8/2019

Reviewer name: Jose Hleap

Reviewer Comments to Author:

The manuscript is a review on the usage metabarcoding in the context of insect control, biosurveillance, and with a special emphasis on pests. It is an important contribution for control agencies around the world who require more throughput in their analysis of invasive species that can be carried in imports and movement of goods. It is also a good contribution to entomologists and molecular biologists looking to incorporate metabarcoding of these taxa into their research. The review includes mostly all steps involved in metabarcoding for surveillance purposes, from selecting a marker to quality assurance. In my opinion, the paper is presented in a coherent and organized manner. However, there are a few points that I consider will make the manuscript stronger, and that it would widen the reach o the paper. I would like to see a wider scope in their searching method (there are a few references missing that I think will improve the manuscript). I also will like to see thorough discussion on the current state of affairs in international law about biosecurity and pest management , since this directly affect the applicability of barcodes in this context. Finally, the manuscript will benefit to have a figure perspectives and conclusions that summarize the author's vision on how to apply barcodes for bio-surveillance.
Below more specific comments:
* Typo in line 73
* Adjust Table2 column width
* In line 152 you make reference to figure 2 when you meant figure 3
* As before but in lines 167 and 168
* It is not clear how can you obtain minimal amplification bias. This is not even clear in the cited articles, and one of them (ref. 83) even explicitly says that it requires in vitro validation and "as many primers might not be suitable for DNA metabardocing due to low base degeneracy, potentially high primer bias or critical design flaws". Therefore the sentences of lines 194-198 is a bit misleading and should be reworded, despite the following sentence introducing other sources of bias.
* Despite the argument about avoiding PCR is compelling, I believe that the author's "over-endorsement" of the micro-array chips is a big leap. It is especially difficult to deliver the appropriate probes when good references are not present, as is one of the arguments of the paper.
* For an interesting discussion on index-switching (discussed around line 257), you can include https://doi.org/10.1111/1755-0998.12928
* Despite that the authors mentioned different sequencing platforms, they seem to restrict their discussion of "demultiplexing and sequence quality trimming" to Illumina pair end reads. They should include at least a paragraph regarding the rest of the platforms discussed

* I think that referencing 150 in line 374 sounds like Elbrecht et al. 2018 used zero-radius OTUs (ZOTU) to investigate intraspecific variation. It is a bit misleading since they used a 3% threshold after the ZOTU inference, essentially making it not an ESV. Some clarification would be needed.
* Fix reference 167
* The authors should be careful with the statement " makes the classification process more robust to pervasive issues of missing and mis-annotated data in reference database". While is true that some ML implementations give some sort of confidence levels in the taxonomic hierarchy, it is not true that it would be less influenced by misannoations and missing references. In fact, most implementations of the Naive bayes will return NA when no significant match with the training set is found. Also, mislabels in the training set will lead to erroneous prediction (https://doi.org/10.1613/jair.606) and is an ongoing challenge in ML to detect mislabels (https://doi.org/10.1007/978-3-319-58628-1_43)
* The statement "In cases where there may be ambiguity due to imperfect reference data and multiple taxonomic outcomes obtain similar probabilities, the sequence may still be robustly assigned to a higher taxonomic rank (e.g. family) [76], providing important information about sample composition and possible presence of novel taxa" is also problematic in my view. The text suggest that Naive bayes by itself has this property, which is not true. Only specific implementations and variations of the algorithm have it. The paper would benefit from exploring this further.
* Some important development is missing in the taxonomic assignment section. The probabilistic method Protax (DOI: 10.1093/bioinformatics/btw346,DOI: 10.1111/2041-210X.12721). Also Axtner et al. (doi: https://doi.org/10.1101/345082) preprint contains a very robust workflow for eDNA, and I think you can extract important information from their discussion. Protax do not require a complete database, gives hierarchical probability of assignment and has a very elegant mathematical framework. It would be a shame if it is not included in your review (or at least mention it to the public).
*I disagree with the authors when they say "While the importance of technical replication for increasing detection probability is generally agreed upon [31,178,179], replicates reduce throughput without providing further independent data points [26]. Instead, with metabarcoding removing one of the major roadblocks to large-scale surveillance, more biological replicates from more frequent and intensive trapping could be used. Considering biological replication is particularly important as regardless of the effectiveness of the metabarcoding diagnostic assay if an insect is not caught in a trap it does not necessarily mean absence in the area". While I agree on the importance of biological replication (and my argument is never against that), technical replicates are of utmost important, especially when dealing with rare species and invasive species. Even if the entire labwork is done in pristine condition, with close to 0 bias, the resulting sequences vary dramatically between replicates. Also, technical replicates inform you of the overall quality of your survey, and allows you to model or correct for biases that might have occur. I think it would be a disservice to the community to let a statement that disregards technical replicates pass. Please consider revising this point.
* In line 541, there is something missing after "similar"
* While I liked and agree with the idea exposed between the lines 541 and 550, the references cited proposed in silico mock communities. Despite this is necessary, I think the authors should also push for the usage of a standard and diverse mock community. The analyses of in silico mock communities and the real mock communities have shown important differences between the two.
* Although Scopus is a large database, it missing a significant amount of citations. I would encourage the

authors to widen their scope to other sources. R has a very good package called fulltext that encompasses multiple sources.

Methods

Are the methods appropriate to the aims of the study, are they well described, and are necessary controls included? Choose an item.

Conclusions

Are the conclusions adequately supported by the data shown? Choose an item.

Reporting Standards

Does the manuscript adhere to the journal's guidelines on minimum standards of reporting? Choose an item.

Choose an item.

Statistics

Are you able to assess all statistics in the manuscript, including the appropriateness of statistical tests used? Choose an item.

Quality of Written English

Please indicate the quality of language in the manuscript: Choose an item.

Declaration of Competing Interests

Please complete a declaration of competing interests, considering the following questions:

- Have you in the past five years received reimbursements, fees, funding, or salary from an organisation that may in any way gain or lose financially from the publication of this manuscript, either now or in the future?
- Do you hold any stocks or shares in an organisation that may in any way gain or lose financially from the publication of this manuscript, either now or in the future?
- Do you hold or are you currently applying for any patents relating to the content of the manuscript?
- Have you received reimbursements, fees, funding, or salary from an organization that holds or has applied for patents relating to the content of the manuscript?
- Do you have any other financial competing interests?
- Do you have any non-financial competing interests in relation to this paper?

If you can answer no to all of the above, write 'I declare that I have no competing interests' below. If your reply is yes to any, please give details below.

I declare that I have no competing interests'

I agree to the open peer review policy of the journal. I understand that my name will be included on my report to the authors and, if the manuscript is accepted for publication, my named report including any attachments I upload will be posted on the website along with the authors' responses. I agree for my report to be made available under an Open Access Creative Commons CC-BY license (http://creativecommons.org/licenses/by/4.0/). I understand that any comments which I do not wish to be included in my named report can be included as confidential comments to the editors, which will not be published.

I agree to the open peer review policy of the journal

To further support our reviewers, we have joined with Publons, where you can gain additional credit to further highlight your hard work (see: https://publons.com/journal/530/gigascience). On publication of this paper, your review will be automatically added to Publons, you can then choose whether or not to claim your Publons credit. I understand this statement.

Yes Choose an item.
